# Common xenobiotics modulate gut microbial responses to low‑calorie sweeteners in vitro

**DOI:** 10.1038/s44320-026-00225-6

**Published:** 2026-06-25

**Authors:** Sonja Blasche, Vinita Periwal, Nonantzin Beristain Covarrubias, Anna Lindell, Indra Roux, Stephan Kamrad, Simone Mozzachiodi, Rob Bradley, Hilal Ozgur, Bini Ramachandran, Vladimir Benes, Kiran Raosaheb Patil

**Affiliations:** 1 Medical Research Council Toxicology Unit, University of Cambridge, Cambridge, UK; 2 European Molecular Biology Laboratory (EMBL), Heidelberg, Germany

**Keywords:** Chromatin, Transcription & Genomics, Microbiology, Virology & Host Pathogen Interaction

## Abstract

The gut microbiota is implicated in adverse effects associated with low-calorie sweeteners. Yet, the direct impact of sweeteners on gut bacteria remains largely uncharacterized. Here, we report interactions between 25 phylogenetically diverse gut bacterial strains and 39 commercially used sweeteners. We tested these sweeteners individually and in combination with four commonly co-consumed compounds, viz., advantame, caffeine, vanillin, and duloxetine. Three-quarters of the tested sweeteners individually impacted the growth of at least one tested bacterial strain. Further, over 100 interactions were found between sweeteners and the four co-consumed compounds. Isosteviol, a commonly used sweetener-component, and duloxetine, an antidepressant, synergistically inhibited *Roseburia intestinalis*, a bacterium previously linked to glucose homeostasis, and *Parabacteroides merdae*, a prevalent commensal linked to healthy microbiota. Proteomic, metabolomic, and genetic analyses indicate altered small molecule transport underpinning this sweetener–drug synergy. The isosteviol-duloxetine combination also modulated metabolism of a synthetic gut bacterial community, leading to increased toxicity to HeLa cells and altered secretion of inflammation-modulatory cytokines IL-6 and IL-8 by Caco-2 cells. Our data warrant further studies on interactions between low-calorie sweeteners and common xenobiotics.

## Synopsis

Low-calorie sweeteners and their combinations with co-consumed xenobiotics were tested in vitro for direct effects on gut bacteria.

- 30 interactions discovered between sweeteners and gut bacteria.
- Sweeteners show synergistic and antagonistic interactions with xenobiotics like antidepressant duloxetine.
- Multi-omics analyses reveal key role of transporters.
- Altered bacterial secretome impact cytokine response of human cells.

## Abstract (web summary)

Low-calorie sweeteners and their combinations with co-consumed xenobiotics were tested in vitro for direct effects on gut bacteria.

## Graphical abstract

**Figure:**
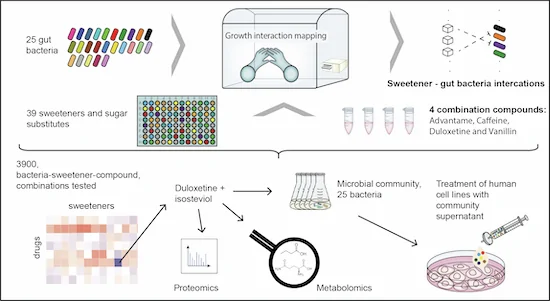

**Figure:**
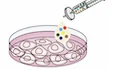

## Introduction

Utilization of low-calorie and non-nutritive sweeteners was anticipated to enhance population health (Russell et al, 2021). However, commonly used sweeteners have now been associated with type II diabetes, obesity, cardiovascular disease, increased cancer risk, and infection (Bian et al, 2017b; Debras et al, 2022; Nobs and Elinav, 2023; Shaher et al, 2023; Witkowski et al, 2023). Further, studies are linking the consumption of artificial sweeteners with pathogenicity (Shil and Chichger, 2021), sucralose with genotoxicity (Schiffman et al, 2023), and aspartame with neurotoxicity and cancer (Shaher et al, 2023). Some effects of sweeteners on human health are likely to be mediated through their interaction with the gut microbiota (Van den Abbeele et al, 2023; Suez et al, 2014). Although several studies have reported interactions between sweeteners and gut microbiota (Bian et al, 2017a, 2017b; Gerasimidis et al, 2020; Gonza et al, 2024; Li et al, 2021; Naim et al, 1985; Nettleton et al, 2020; Palmnäs et al, 2014; Sünderhauf et al, 2020; Vamanu et al, 2019; Wang et al, 2018; Castañeda-Monsalve et al, 2024; Nobs and Elinav, 2023; Sun et al, 2022), systematic studies testing for direct interactions between phylogenetically diverse gut bacteria and sweeteners are scarce. Further, gut bacteria are likely to encounter sweeteners in conjunction with other xenobiotics, such as medications or food additives. Recent studies have highlighted the importance of studying the effects of co-consumed food compounds on gut bacteria (Culp et al, 2024). However, mechanistic insight into the interactions among sweeteners, xenobiotics, and gut bacteria remains limited, highlighting the need for accessible in vitro approaches to complement correlative analyses of fecal microbial communities and in vivo models.

Commercial sweeteners are widely used in processed foods like soft drinks, candy, chewing gum, and cereal, and are frequently co-formulated with other sweeteners, the flavor compound vanillin, and the neurostimulator caffeine. Sweeteners also function as excipients in various pharmaceuticals (Rowe et al, 2009), such as antihypertensives, and are co-ingested with long-term medications like antidepressants that are consumed by millions of people daily (World Population Review, 2022). The exposure of a significant portion of the population to sweeteners in conjunction with other food compounds and drugs raises questions about the interactive effects on gut bacteria. To our knowledge, sweeteners and common xenobiotics have not been tested systematically for combinatory effects on human gut bacteria, which can be highly relevant as exemplified by multi-antibiotic-resistance phenotype in *E. coli* resulting from the combination of antimicrobial compounds with vanillin (Brochado et al, 2018).

Previous studies examining the impact of sweeteners on the gut microbiota and gut bacteria (Van den Abbeele et al, 2023; Sun et al, 2022; Jia et al, 2024) suggest that these effects are widespread across different bacterial phylogenetic groups. This includes compound-specific effects, such as a product-specific increase in SCFA production for six human samples (Van den Abbeele et al, 2023). Moreover, it has been demonstrated that the consumption of select sweeteners, when combined with a high-fat diet, can alter the composition of the gut microbiota in mice and rats, thereby affecting both microbiota dynamics and host metabolic interactions (Palmnäs et al, 2014; Chi et al, 2018; Sánchez-Tapia et al, 2020; Nettleton et al, 2020). In addition, exposure to sweeteners has been shown to alter fermentation profiles and metabolism in vitro, indicating a connection between sweetener exposure and modifications in both microbiota composition and microbial metabolic processes (Vamanu et al, 2019). Dataset EV1 summarizes a variety of prior in vivo and in vitro studies. These studies may imply either direct effects or indirect, emergent effects that may arise in a community context as observed in the case of therapeutic drugs (Garcia-Santamarina et al, 2024), or from mixture effects involving co-consumed foods or compounds. Analysis of the direct effects of sweeteners on bacteria are needed to understand the mechanisms through which these compounds impact microbiota changes.

In this study, we conducted a comprehensive screening of all commercially available sweeteners and sugar substitutes, both natural and synthetic (totaling 39), to assess their effects on the growth of 25 strains of human gut bacteria (Datasets EV2 and EV3). The 25 bacteria were selected to cover a broad phylogenetic and metabolic diversity representative of the healthy human gut microbiota (Tramontano et al, 2018), and to span commensals, probiotic species, and opportunistic pathogens. The 39 sweeteners cover all commercially used sweeteners that could be purchased as pure compounds. Additionally, we examined the combined effects of sweeteners with four commonly co-consumed compounds: advantame, caffeine, vanillin, and duloxetine. The four combination compounds were chosen due to their widespread usage. Advantame is a non-caloric artificial sweetener that is increasingly being used and is known to reach the gut (EFSA, 2013; Reports Global, 2024). Caffeine and vanillin are found in, among many other products, beverages, cakes, candies, and in the case of vanillin even in baby formula (Arthur Nelson, 2019). Duloxetine is a commonly used antidepressant drug (Alvarez-Mon et al, 2023), which has been shown previously to have significant effects on metabolite secretion in gut microbes (Klünemann et al, 2021). In the years 2022 and 2023, it was the 31st most prescribed drug with more than 18 million prescriptions and more than 4 million patients in the United States alone (https://clincalc.com/DrugStats/Drugs/Duloxetine). Thus, these compounds are commonly consumed by millions of individuals (Karatoprak, 2022; Reyes and Cornelis, 2018). In addition, we tested combinations of sweeteners with commonly used tablet-formulated drugs—acetaminophen, aripiprazole, cetirizine, ethinylestradiol, ibuprofen, montelukast, ranitidine, and risperidone—which are typically co-formulated with sweeteners to mask bitterness. This, together with the high usage of sweeteners, indicates that a substantial number of people are co-consuming sweeteners and the xenobiotics chosen in our study.

## Results

### Impact of sweeteners on bacterial growth

**Figure 1:**
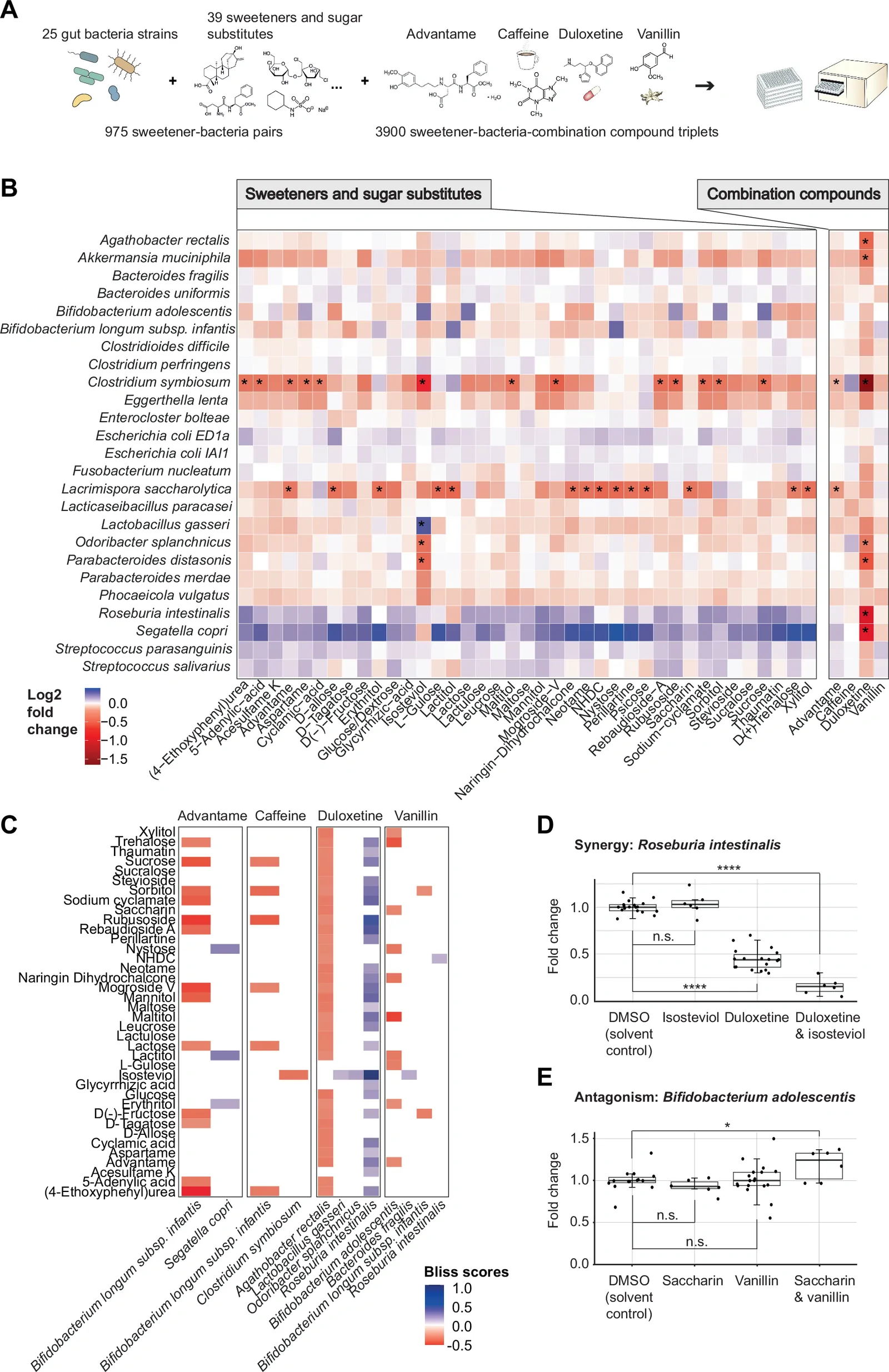
Interactions between sweeteners and gut bacteria. (**A**) Schematic overview of the sweetener-bacteria screening set-up. (**B**) Identified interactions between sweeteners and human gut bacteria. All compounds were tested at 50 µM concentration. Interactions were deemed significant (marked with *) when, (1) bh-corrected (Benjamini and Hochberg, 1995) *P* value < 0.05 (Welch’s *t* test), (2) > 20% change in growth (area under the growth curve, AUC) compared to the solvent control, and (3) confirmed in an independent experiment including additional concentrations as described in the main text. *N* = 6 (3 biological times 2 technical replicates). (**C**) Significant sweeteners-xenobiotic interactions impacting gut bacterial growth. Bliss score for the significant interactions (bh-corrected *P* value < 0.05, and absolute (log2 fold change) >0.32) is shown in the colormap, see Dataset EV5 for full screening results. *N* = 6 (3 biological times 2 technical replicates). (**D**) Example of synergistic interaction between isosteviol and duloxetine affecting growth of *R. intestinalis* (DMSO solvent control vs duloxetine and isosteviol: *P* value = 9.10218E-40, Welch’s *t* test, *N* ≥ 6, box plot statistics: Q1 (25%): 0.55, median: 0.98, Q3 (75%): 1.02, IQR: 0.47, lower whiskers: 0.05, upper whiskers: 1.24, minimum: 0.05, maximum: 1.24. (**E**) Example of an antagonistic interaction between saccharin and vanillin affecting the growth of *B. adolescentis*, *P value = 0.020376, *N* ≥ 6. box plot statistics: Q1 (25%): 0.94, median: 1, Q3 (75%): 1.085, IQR: 0.145, lower whiskers: 0.7225, upper whiskers: 1.3025, minimum: 0.55, maximum: 1.43. If not stated otherwise, significance was determined using Welch’s *t* test. *P value<0.05, **P value <0.01, ***P value <0.001, ****P value <0.0001. Error bars represent s.d. Source data are available online for this figure.

We first investigated the growth effects of 39 individual sweetener molecules on the 25 gut bacterial strains (975 sweetener-bacteria pairs in total, Fig. 1A, “Methods”). To ensure reliable growth of the selected phylogenetically diverse species, we chose mGAM (modified Gifu anaerobic medium broth, HyServe) as growth medium. This medium supports the growth of all selected species in monocultures and has been used in previous studies (Tramontano et al, 2018; Van Den Berg et al, 2024; Maier et al, 2021), enabling comparative analysis. Furthermore, mGAM medium closely aligns with the in vivo relative abundance in healthy subjects compared to monoculture growth (Tramontano et al, 2018). To enable comparison across all sweetener compounds, we used a concentration of 50 µM which we estimated within the range of concentrations relevant for the colon (Dataset EV4).

The sweetener–bacteria interactions were tested in two independent experiments, whereby the interactions meeting significance thresholds (*P* < 0.05 and ≥20% growth change) in the first screen were retested across five concentrations (25–400 µM). True positive interactions were assessed as those with significance (Welch’s *t* test, *P* < 0.05) in both experiments and with consistent effects at ≥2 concentrations (Dataset EV8). We observe high overlap between the two experiments, i.e., the initial screen and the second experiment at multiple concentrations (> 90% overlap, 30 out of 33 interactions, Appendix Figs. S1 andS2; Datasets EV5–10). Thus, in total we identified 30 interactions between 5 bacterial strains and 26 sweeteners and sugar substitutes (marked with * in Fig. 1B).

While some direct sweetener-bacteria interactions, such as between stevia glycosides and lactobacilli, have been described (Van Hul and Cani, 2023) (overview in Dataset EV1), the 30 direct interactions identified in this study have not been previously reported to our knowledge. *Clostridium symbiosum* or *Lacrimispora saccharolytica* were the most sensitive species being affected by many compounds. On the sweetener side, isosteviol was the most potent compound, inhibiting the growth of three bacteria (*C. symbiosum, Odoribacter splanchnicus*, and *Parabacteroides distasonis*) while promoting the growth of one, *Lactobacillus gasseri*.

### Antagonistic and synergistic effects of sweetener–xenobiotic combinations

To assess whether other consumed compounds can alter the effect of sweeteners on gut bacteria, we screened 156 sweetener–xenobiotic combinations (39 sweeteners × 4 xenobiotics) against 25 bacteria (3900 interactions in total). We also tested 12 sweetener combinations with 8 commonly used drugs, namely acetaminophen, aripirazole, cetrizine, ethinylestradiol, ibuprofen, montelukast, ranitidine, and risperidone (300 interactions in total), reflecting typical tablet formulations (see Dataset EV11 for details). For example, acesulfame potassium is frequently combined with ibuprofen, acetaminophen, and cetirizine. Inter-compound interactions were evaluated using the Bliss model of independence (Bliss, 1939; Demidenko and Miller, 2019) (Appendix Fig. S3) with significance determined by a *P* value threshold of 0.05 and a 20% growth change relative to each compound alone (“Methods”, Datasets EV11–12; Appendix Fig. S4). This model allowed us to classify compound–compound interactions as antagonistic if the observed combined effect is less than the product of the individual compound effects, or synergistic if it exceeds this product.

The compound combination screen revealed 102 interactions involving nine gut bacteria (Fig. 1C, *P* value < 0.05 and log2 fold change (L2FC) <−0.32 and >0.32), 68 of which were antagonistic and 34 synergistic. To our knowledge, none of these interactions has been reported before. The higher prevalence of antagonistic interactions is consistent with those observed for drug combinations (Brochado et al, 2018), and with the expectation that the distinct compounds generally target distinct pathways. The sweetener isosteviol was notable in our results, exhibiting combinatory effects with three of the four tested xenobiotics and affecting five bacterial species. The strongest synergy was observed between isosteviol and duloxetine against *R. intestinalis* (Fig. 1D), a bacterium positively associated with health and the prevention of intestinal inflammation (Nie et al, 2021). Combinations showing antagonistic interactions included vanillin and saccharin, which are frequently co-used in processed food products (Fig. 1E).

### Duloxetine-isosteviol combination reduces community diversity

**Figure 2:**
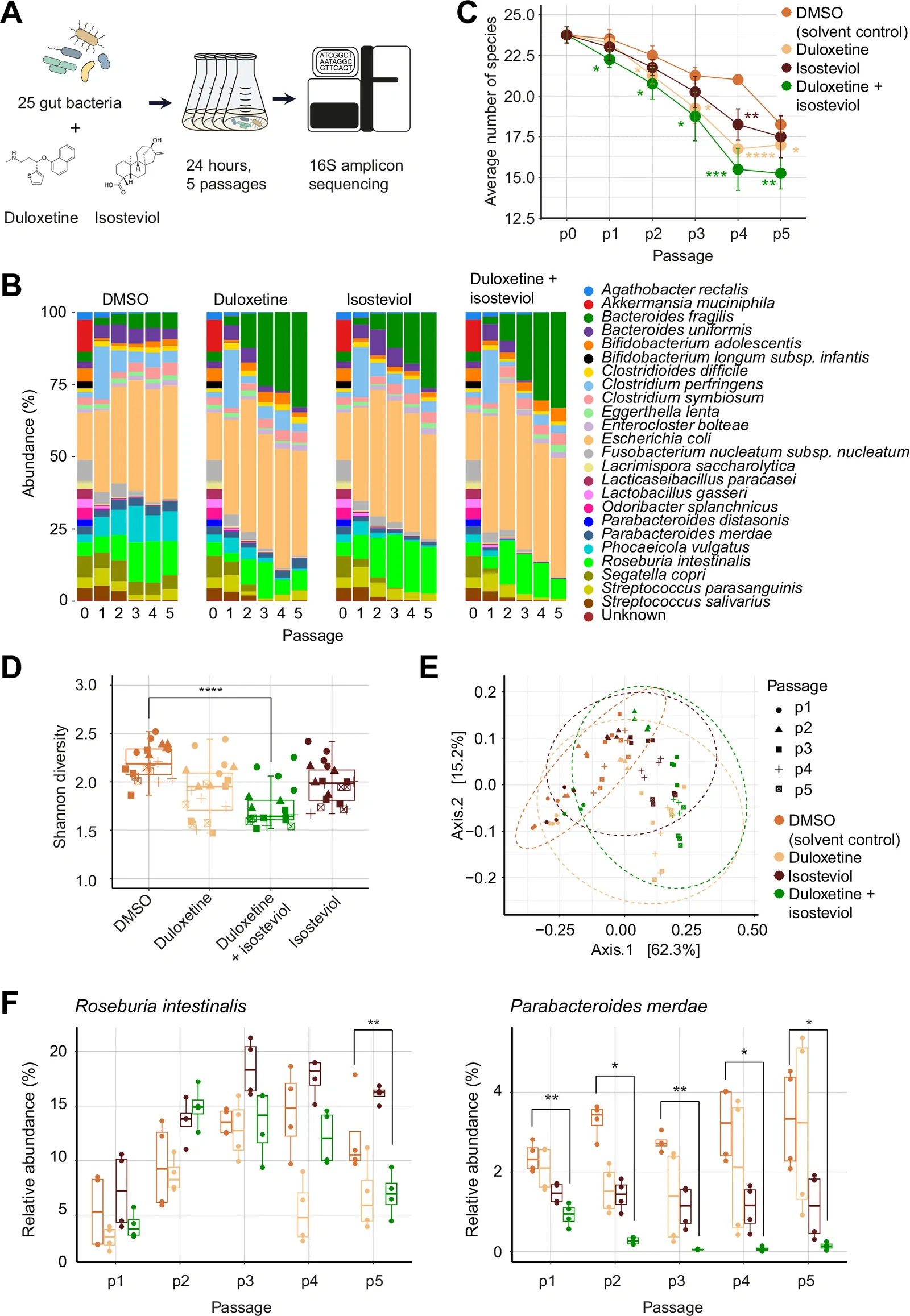
Impact of duloxetine-isosteviol combination on a synthetic gut bacterial community. (**A**) Experimental approach. (**B**) Community compositional dynamics in compound-treated communities assessed using 16S rRNA analysis, *N* = 4. (**C**) Number of surviving species during serial transfer in the control and the compound-treated communities. Lost species cutoff, <10 reads. *N* = 4, Student’s *t* test: *P* value: duloxetine: p2 * = 0.016964736, p3 * = 0.010049242, p4 **** = 2.6496E-06, p5 * = 0.040057803, isosteviol: p4 ** = 0.001210357, duloxetine and isosteviol p1 * = 0.016964736, p2 * = 0.020311818, p3 *=0.019508636, p4 *** = 0.000143179, p5 * = 0.001439526, see Dataset EV16 for complete list of *P* values. (**D**) Changes in alpha diversity (Shannon) between compound-treated samples. ****P value = 1.31E-08 (ANOVA, see Dataset EV15 for Shannon diversity per replicate and significance for all group comparisons), *N *= 4 (5 passages). Box plot statistics: Q1 (25%): 1.76, median: 2.02, Q3 (75%): 2.33, IQR: 0.58, lower whiskers: 1.47, upper whiskers: 2.95, minimum: 1.47, maximum: 2.95. (**E**) Results of principal coordinate analysis showing Bray–Curtis beta-diversity across all passages and treatment groups. *N* = 4. (**F**) Changes in the relative abundance of *R. intestinalis* and *P. merdae* during community passaging. *N *= 4, Student’s *t* test, *R. intestinalis*: p5 ** = 0.004263188, *P. merdae*: p1 ** =  0.004090449, p2 * = 0.016099707, p3 ** = 0.005177468, p4 * = 0.012859709, p5 * = 0.026312174. Box plot statistics: Q1 (25%): 1.55, median: 3.58, Q3 (75%): 10.83, IQR: 9.28, lower whiskers: 0, upper whiskers: 21.23, minimum: 0, maximum: 21.23. Source data are available online for this figure.

To assess how the impact on individual species can manifest at the community level, we tested the effect of the duloxetine-isosteviol combination in a synthetic community assembled from the 25 gut bacteria used in the screen. The assembled community was passaged five times in the presence of DMSO (solvent control), isosteviol, duloxetine, and the isosteviol-duloxetine combination (50 µM each, “Methods”). Community composition was assessed directly after community assembly (p0) and after each passage (24 h growth, p1-p5), (Fig. 2A; Datasets EV13 and 14). All assemblies exhibited early-transfer compositional dynamics that stabilized around passage p3–p4 using the F*st* statistic from the FAVA framework (Morrison et al, 2025), albeit with some variability in species counts of low-abundance species whose frequency fluctuates around our limit of detection (Fig. 2B; Appendix Fig. S5; Dataset EV15). This assembly dynamics is consistent with previous experiments with in vitro assembled gut bacteria communities (Aranda-Díaz et al, 2022; Goldford et al, 2018).

A significant decrease in overall species diversity was observed for all compound-treated samples (Fig. 2C; Dataset EV16) and most markedly in the case of the isosteviol-duloxetine combination (Fig. 2D,E). Samples challenged with the combination showed decreased relative abundance (as compared to the DMSO control) for several species, among them *R. intestinalis*, *P. merdae*, *Segatella copri*, *B. uniformis*, and *P. vulgatus* (Appendix Fig. S6 and Dataset EV17). The relative abundance of *R. intestinalis* in the combination-treated communities was significantly altered in p5, whereas that of *P. merdae* was impacted in all passages (Fig. 2F).

In the case of *P. merdae*, its relative abundance in the combination-treated community was significantly lower in many passages compared to treatments with either compound alone (Dataset EV18), indicating a strong combination effect. Correspondingly, ANOVA revealed a significant change in *P. merdae* abundance, while no such effect was observed for *R. intestinalis* (Dataset EV19). Notably, *R. intestinalis* exhibited a synergistic effect in monoculture, but not in community, whereas the reverse was true for *P. merdae*. This contrast suggests emergent effects within the community that extend beyond the direct impacts of isosteviol and duloxetine on isolated gut bacteria. In addition, of the 25 bacteria screened, 24 showed reduced growth in monoculture after treatment with duloxetine and isosteviol, while 7 benefited in community settings. These community effects may involve altered metabolic activity of community members as previously observed with human-targeted drugs (Klünemann et al, 2021).

### Proteomic basis of the isosteviol–duloxetine synergy

**Figure 3:**
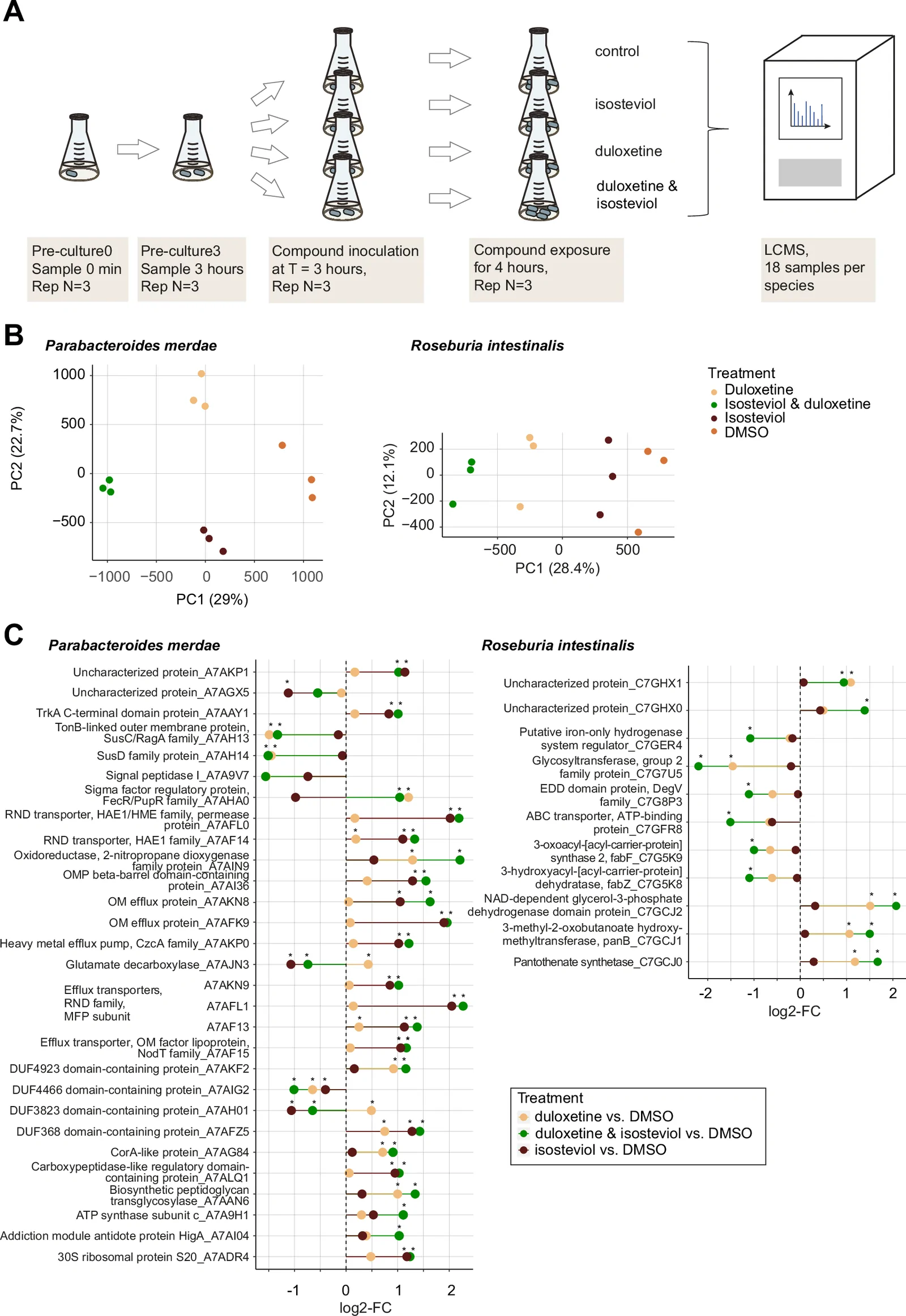
Proteomics analysis provides insights into the synergistic effects of the isosteviol-duloxetine combination. (**A**) Experimental design. To align cultures, bacteria were pre-grown until the logarithmic phase (OD of ~0.6) was reached, then split into 4 aliquots, and compound treatment was started and performed for 4 h, before cultures were harvested for proteomics. (**B**) Differences in overall protein expression between compound-treated bacteria and control (DMSO). (**C**) Differently expressed proteins in treated samples normalized to DMSO-treated samples (control), *N* = 3, cut-off: log2-FC = 1 and −1. OM outer membrane, OMP outer membrane protein. Conditions where proteins are significantly differentially expressed are marked with a star, ANOVA, bh-corrected *P* value < 0.05, exact *P* values are provided in Dataset EV43-44. Source data are available online for this figure.

To investigate the molecular basis of the isosteviol–duloxetine interaction, we analyzed proteomic changes in *R. intestinalis* and *P. merdae* following exposure to each compound individually and in combination. *R. intestinalis* was selected as it exhibited a strong synergistic response in monoculture. While *P. merdae* showed a weaker effect that did not meet the criteria for a Bliss interaction in our screen (BH-corrected *P*  <  0.05 and (log₂ fold change) > 0.32; Appendix Fig. S7), it displayed a significant interaction effect in the synthetic community (Fig. 2F). To control for proteomic changes due to growth-phase differences, both species were pre-cultured to reach logarithmic growth before 4 h of compound exposure (Fig. 3A, “Methods”). This approach minimized growth-related variability, which can exceed that induced by compound treatment (Appendix Fig. S8) (Mori et al, 2021; Muthusamy et al, 2017).

Principal component analysis of proteomic changes showed good concordance with the observed synergistic growth effects (Fig. 3B). Overall, the protein abundance changes in the combination-treated cells differed from the null expectation of a purely additive effect of single compound treatments (Appendix Fig. S9). Eleven proteins in *R. intestinalis* and 29 in *P. merdae* exhibited differential abundance upon co-exposure (Fig. 3C; Datasets EV20–23). There was no overlap between the responding proteins from the two species, suggesting distinct underlying mechanisms. The response of *P. merdae* involved 16 cell envelope-associated proteins, while *R. intestinalis* exhibited many changes in the vitamin B5 pathway, i.e., increased abundance of PanB, PanC, and C7GCJ2—a putative PanG based on gene location and similarity in the pantothenate operon (Miller et al, 2013)—and fatty acid metabolism (Fig. 3C).

To test whether vitamin B5 metabolism could modulate the synergy between isosteviol and duloxetine, we tested 20 different metabolic supplements and mixes for both *R. intestinalis* and *P. merdae*. The tested supplements included calcium pantothenate, naftidrofuryl-oxalate (an inhibitor of vitamin B5 synthesis), a mix of essential vitamins and minerals, a mixture of amino acids, sodium chloride, proteinase K, beta-alanine, butyrate, succinate, acetate, coenzyme A, ATP, additional glucose, folate, trimethoprim (an inhibitor of folate biosynthesis), all in different concentrations and combinations. However, none of these factors influenced the sensitivity of *R. intestinalis* or *P. merdae* to isosteviol, duloxetine, or their combination (see Appendix Fig. S10). Vitamin B pathway proteins are thus not likely to be the primary targets of duloxetine or isosteviol.

In the case of *P. merdae*, we found 29 proteins uniquely responding to the compound combination, albeit with an imbalance between the up- (21) and the down- (5) regulated proteins (Fig. 3C). Three were upregulated by one compound and downregulated by the other (sigma factor regulatory protein FecR/PupR family, glutamate decarboxylase, and DUF3823 domain−containing protein). Among the upregulated proteins, twelve are subunits of cell envelope proteins, transporters, or efflux pumps. Among the five downregulated proteins are the SusC and SusD subunits of the saccharide uptake system, a transporter for the uptake of large nutrients through the outer membrane (Gray et al, 2021). CorA-like protein, which is a putative magnesium transporter, had increased abundance, albeit just below the twofold cut-off, upon combined duloxetine-isosteviol exposure. Collectively, these changes suggest *P. merdae* uses metabolite transport to counter the inhibitory combination of isosteviol and duloxetine.

### A forward genetic screen reinforces the role of transporters in sweetener toxicity

To identify genetic determinants underlying bacterial response to single and co-compound treatment, we used an in-house barcoded transposon mutant library of *P. merdae* (Tn-Bar-Seq) spanning all non-essential genes (circa 3000) (Voogdt et al, 2025). The pooled library includes over 50 unique barcoded insertion strains per gene, which are quantified using barcode next-generation sequencing to assess gene fitness changes across perturbations (“Methods”).

**Figure 4:**
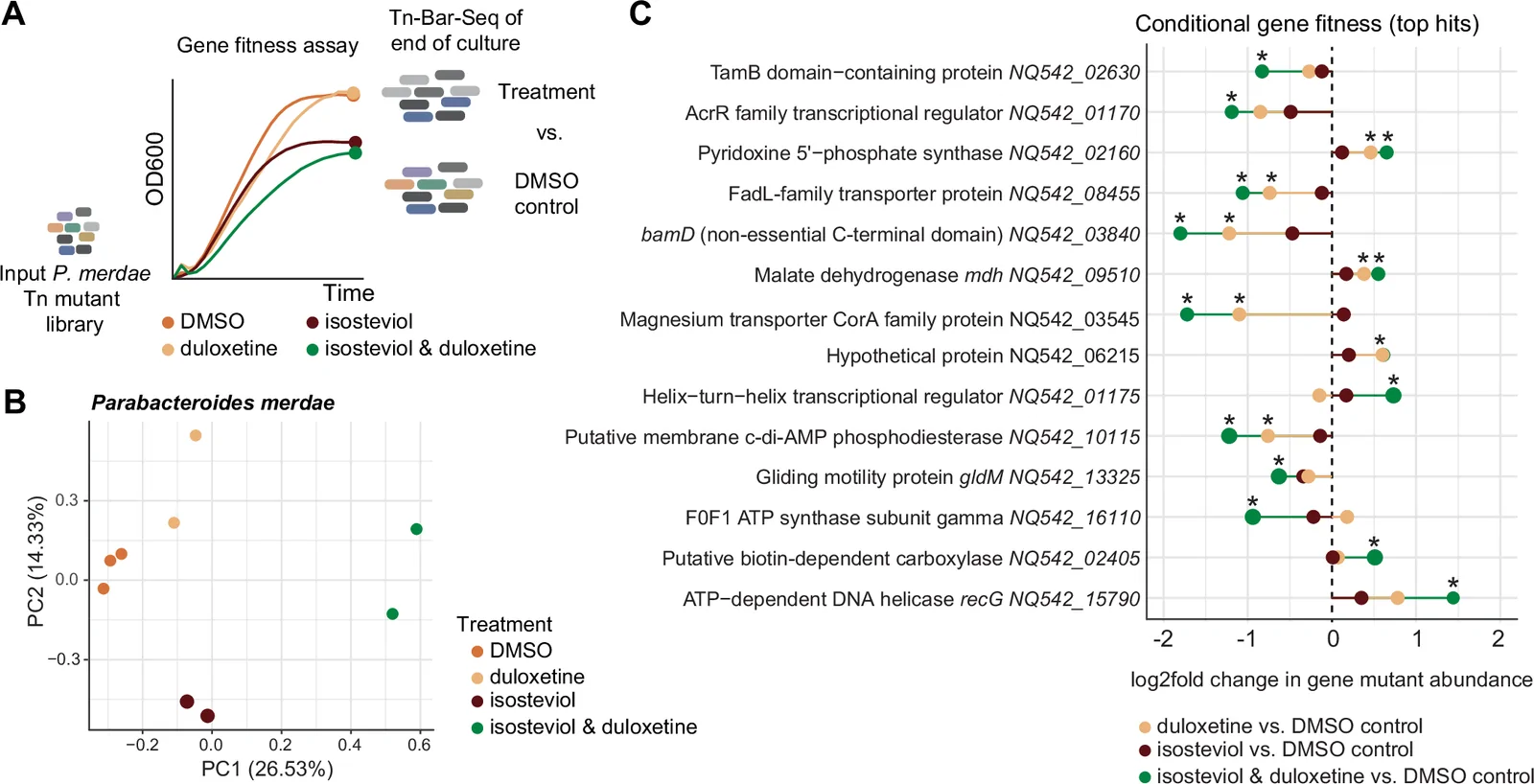
Genetic elements underlying isosteviol-duloxetine synergy. (**A**) Experimental design and representative Tn library growth curves as measured from a 100 µl sub-sample. A pooled genome-wide mutant library of *P. merdae* was used to inoculate mGAM with the different chemical treatments or a DMSO control, following a similar growth trajectory to the WT strain. The changes in mutant abundance at the end of culture were analyzed by deep sequencing of the barcoded transposons (Tn-Bar-Seq, “Methods”). (**B**) Principal component analysis of mutant gene abundance across treatments shows a synergistic effect in the combination. (**C**) Conditional gene fitness by TnBarSeq for each condition, relative to the DMSO control. *N* = 2 treatment, *N* = 3 DMSO, *P* adj. <0.05 and absolute log2fold > 0.5 in at least one condition. Conditions with significant gene fitness are marked with * (TRANSIT resampling, bh-corrected *P* value < 0.05), exact *P* values are provided in Dataset EV45. Source data are available online for this figure.

The *P. merdae* mutant library was grown under isosteviol, duloxetine, or co-exposure, and the resulting compositional frequency was compared to that treated with the vehicle (DMSO) (Fig. 4A). In line with the enhanced inhibitory effect on growth, the co-exposure to isosteviol and duloxetine showed the largest variation in the endpoint mutant library composition (Fig. 4B).

To define gene hits, we set an absolute log2 fold change cutoff of 0.5 for conditional fitness, which is the proportion of gene mutants at the endpoint of chemical perturbation compared to the DMSO control. Thirteen mutants responded to co-exposure, and five to duloxetine (Fig. 4C; Dataset EV24). The strongest conditional negative fitness (loss of mutants) maps to genes encoding membrane proteins, suggesting their key role in survival during co-exposure. These include a putative magnesium transporter of the CorA family (NQ542_03545), a putative hydrophobic compound transporter of the Fadl family (NQ542_08455), a non-essential domain of the outer membrane protein assembly factor BamD (NQ542_03840), and a putative c-di-AMP phosphodiesterase (NQ542_10115) (Appendix Fig. S11). NQ542_10115 is an ortholog of the Pde c-di-AMP regulator implicated in cell membrane homeostasis in *Porphyromonas gingivalis* (50% identity, 94% coverage) (Moradali et al, 2022). When comparing Tn-Bar-Seq gene fitness score with the proteomics data, *corA*-family transporter NQ542_03545 stands out as a candidate with strong negative fitness and increased protein abundance both for duloxetine and co-exposure (Appendix Fig. S11). However, magnesium supplementation did not subvert compound exposure phenotype in *P. merdae* (Appendix Fig. S12), suggesting this transporter has an alternate, yet unknown, role in cellular homeostasis.

The mutant for putative transcriptional regulator *acrR* (NQ542_01170), which is an ortholog of an efflux regulator in *Phocaeicola vulgatus*(Hibberd et al, 2017*)*, exhibited negative fitness in accord to chemical transport changes (Appendix Fig. S11). While the above-mentioned genes were also significant hits for duloxetine alone, their negative fitness was further exacerbated when isosteviol was present. Among the other top gene hits are genes from central metabolism and vitamin synthesis, which are often observed for xenobiotics having a mode of action different from canonical antibiotics (Noto Guillen et al, 2024). Overall, both approaches confirm that membrane integrity and transport are crucial for survival under the duloxetine-isosteviol combination.

### Isosteviol and duloxetine alter bioaccumulation

Previously, we observed that duloxetine bioaccumulation can alter metabolism in gut bacteria (Klünemann et al, 2021). We therefore assessed whether the synergistic effects of duloxetine and isosteviol are reflected in, or contributed to, by the differences in isosteviol and duloxetine bioaccumulation by *P. merdae* and *R. intestinalis*. For comparison, we also included *S. copri*, a bacterium that shows significant inhibition by duloxetine in monoculture and a marked decrease in the community when treated with either duloxetine alone or co-treated with isosteviol. While *S. copri* bioaccumulated both compounds to a similar extent under all tested conditions, *R. intestinalis* bioaccumulated isosteviol only when duloxetine was present (Appendix Fig. S13; Datasets EV25–26). On the other hand, *P. merdae* bioaccumulated isosteviol in the absence of duloxetine. The altered bioaccumulation is consistent with the altered metabolite transport suggested by the proteomic and genetic analyses and likely contributes to the synergy between isosteviol and duloxetine.

### Duloxetine and isosteviol exposure affect secreted metabolites

Genetic analysis and altered bioaccumulation both indicated changes to cross-membrane transport of small molecules. We therefore hypothesized that co-exposure of duloxetine and isosteviol alters the metabolite secretome. To test this, we used untargeted liquid chromatography mass spectrometry (LC-MS) to characterize secreted metabolites as well as whole culture metabolic profile (cells and supernatant) of *S. copri*, *R. intestinalis*, and *P. merdae* (“Methods”, Datasets EV27–32).

**Figure 5:**
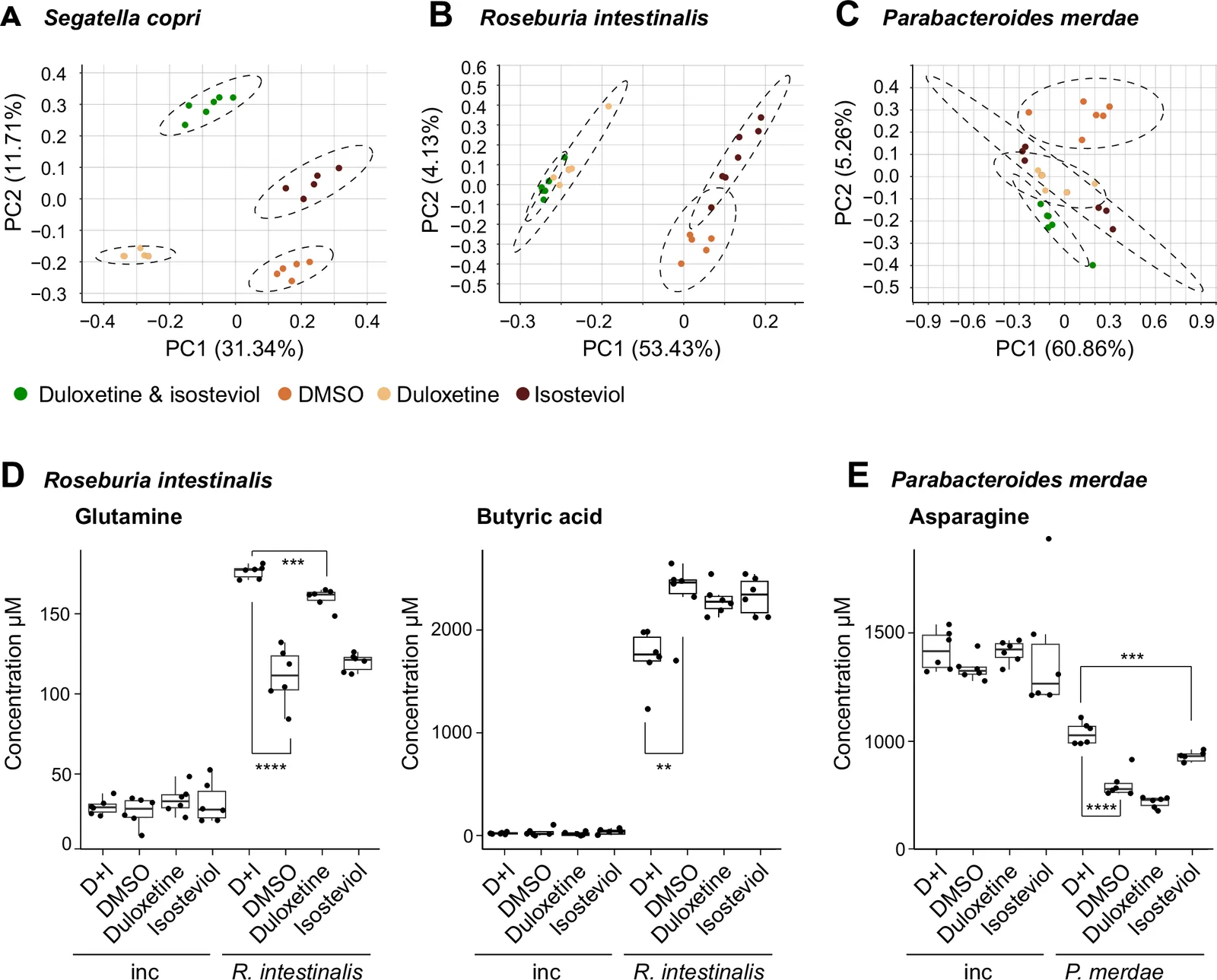
Isosteviol and duloxetine alter metabolic physiology. Altered metabolite production in culture supernatant of (**A**) *S. copri*, (**B**) *R. intestinalis*, and (**C**) *P. merdae* upon exposure to isosteviol, duloxetine, and a combination of both. Metabolites (captured in negative mode, using LC-MS, qTOF) cluster in a treatment-dependent way. Biological replicates: *N* ≥ 5, DMSO = mock, solvent control. (**D**) Changes of primary metabolites glutamine (****P value: 4.91407E-06 and ***P value: 0.000249633, box plot statistics: Q1 (25%): 29.32, median: 68.42, Q3 (75%): 136.24, IQR: 106.91, lower whiskers: 11.51, upper whiskers: 181.42, minimum: 11.51, maximum: 181.42), butyric acid (**P value: 0.00589848, box plot statistics: Q1 (25%): 18.64075, median: 668.10, Q3 (75%): 2266.96, IQR: 2248.32, lower whiskers: 0, upper whiskers: 2647.52, minimum: 0, maximum: 2647.52) in *R. intestinalis* and (**E**) asparagine (****P value: 2.43146E-05 and ***P value: 0.000866275, box plot statistics: Q1 (25%): 902.49, median: 1161.54, Q3 (75%): 1349.88, IQR: 447.39, lower whiskers: 679.63, upper whiskers: 1934.11, minimum: 679.63, maximum: 1934.11) in *P. merdae* upon 24 h co-exposure to duloxetine and isosteviol. D + I, duloxetine + isosteviol; inc, incubation control (medium plus compounds). Significance was determined using Student’s *t* test (**D**, **E**): *N* = 6. Source data are available online for this figure.

The three tested species showed different responses to the compound exposures (Fig. 5A–C; Appendix Fig. S14). For *S. copri*, both the whole culture and supernatant metabolites clustered by treatment, indicating a co-treatment effect on the metabolome (Fig. 5A). In monoculture, no combinatory effect was observed; however, community growth was reduced by both single compounds and co-exposure compared to DMSO. Thus, the altered metabolism may contribute to the decreased competitiveness of *S. copri* in the microbial community (Fig. 2E; Appendix Fig. S6).

The metabolites of *R. intestinalis* (both, whole culture and supernatant) clustered by duloxetine treatment (Fig. 5B). The impact of isosteviol was minor and similar to that of the DMSO control, reflecting the changes observed in protein abundances wherein the effect of duloxetine was dominant compared to that of isosteviol (Fig. 3C). The combinatory growth effects in *R. intestinalis* may thus be mainly due to duloxetine-dependent alterations in isosteviol bioaccumulation (Appendix Fig. S13).

In the case of *P. merdae*, whole culture metabolites clustered based on the corresponding treatments. Yet, for the supernatant, all treatments cluster together, separate from the vehicle (DMSO) (Fig. 5C; Appendix Fig. S14c), albeit with a slight separation of co-treated samples from DMSO. The similar secretion profiles for both mono- and co-exposure suggest the key role of altered abundance of membrane proteins and transporters as revealed by proteomics analysis (Fig. 3C).

To gain indications on the nature of the impacted metabolites, we selected 19 peaks from the untargeted analysis that showed significantly different abundance in the three microbes treated with duloxetine and isosteviol as compared to the vehicle treatment. Of the 19 peaks, sixteen could be annotated as di- and tripeptides based on matching of MS2 spectra to an online database using the PEP search tool (Tang et al, 2014) (Appendix Fig. S15; Dataset EV33, “Methods”). For all three species, both increases and decreases in di- and tripeptides as a response to the combined treatment were observed. Microbial di- and tripeptides have been shown to have antihypertensive properties (Vukic et al, 2017) and to be involved in *Listeria monocytogenes* infection (Reniere et al, 2015) and intestinal inflammation (Charrier et al, 2006; Dalmasso et al, 2010). In conclusion, the identified di- and tripeptides, which showed altered abundance in response to duloxetine and isosteviol treatment, may influence various biological processes and modulate the host’s response to microbial presence.

### Co-exposure impacts primary metabolism in *R. intestinalis* and *P. merdae*

To further pinpoint the metabolic effects of isosteviol and duloxetine, we used targeted LC-MS analysis to quantify 46 metabolites (32 amino acids and derivatives, 10 organic acids, and 4 short-chain fatty acids) in *S. copri*, *R. intestinalis*, and *P. merdae* (Datasets EV34–36). For *S. copri*, three metabolites showed significant (*P* < 0.05) changes in response to the duloxetine-isosteviol combination: cadaverine, nicotinic acid, and pyridoxal (Appendix Figs. S16, S17, and S18). *R. intestinalis* showed changes in glutamine, butyric acid, and isovaleric acid. Notably, glutamine production increased by circa 50%, and butyric acid decreased by more than 25% upon co-exposure to duloxetine and isosteviol (Fig. 5D; Appendix Figs. S17, S18, and S19).

In absolute terms, the concentrations of glutamine and butyric acid changed substantially, by circa 65 µM and 0.6 mM, respectively, thus implying relevance for microbe–host interaction. Although it remains unclear to what extent increased glutamine production by *R. intestinalis* contributes to total glutamine levels in the colon, it has previously been shown that increased gut glutamine levels can be beneficial (anti-inflammatory) and detrimental (among others, increased risk of digestive system cancers) (Xie et al, 2024; Kim and Kim, 2017; Nan et al, 2025). Butyric acid has previously been associated with anti-inflammatory properties, increased insulin sensitivity, and protection against diet-induced obesity (Gao et al, 2009; Lin et al, 2012; Mahalak et al, 2020), and these effects have also been linked to *R. intestinalis (*Gurung et al, 2020*;* La Rosa et al, 2019*;* Xu et al, 2022*)*. In contrast, increased isovaleric acid concentrations in feces have been shown to correlate with depression (Szczesniak et al, 2016). In *P. merdae*, compound co-exposure led to significant changes in agmatine, asparagine, and pyridoxamine concentrations. While total concentrations of agmatine and pyridoxamine are in the lower µM range, the concentration of asparagine changed by >200 µM, indicating substantial changes in amino acid metabolism (Fig. 5E; Appendix Figs. S20, S17, and S18). The altered peptide concentration uncovered by the untargeted analysis further highlights the impact on amino acid metabolism. Together, synergistic effects of isosteviol and duloxetine on gut bacteria alter the secretion of metabolites linked to host health.

### Altered microbial metabolism modulates toxicity and cytokine secretion by human cells

Gut bacterial metabolites are key mediators of microbiota-host interaction (Zhang et al, 2023). In the context of artificial sweeteners, longitudinal studies have shown altered serum levels of gut microbial metabolites (Jia et al, 2024). Therefore, we next examined whether metabolic changes caused by the isosteviol-sweetener exposure impact host cells. We first tested the toxicity of supernatants from the 25-member gut bacterial community (Fig. 2) exposed to isosteviol, duloxetine, and their combination on HeLa cells. HeLa cells are a robust model commonly used in general toxicity screens, and here serve as a reference point to assess potential effects of an altered secretome.

**Figure 6:**
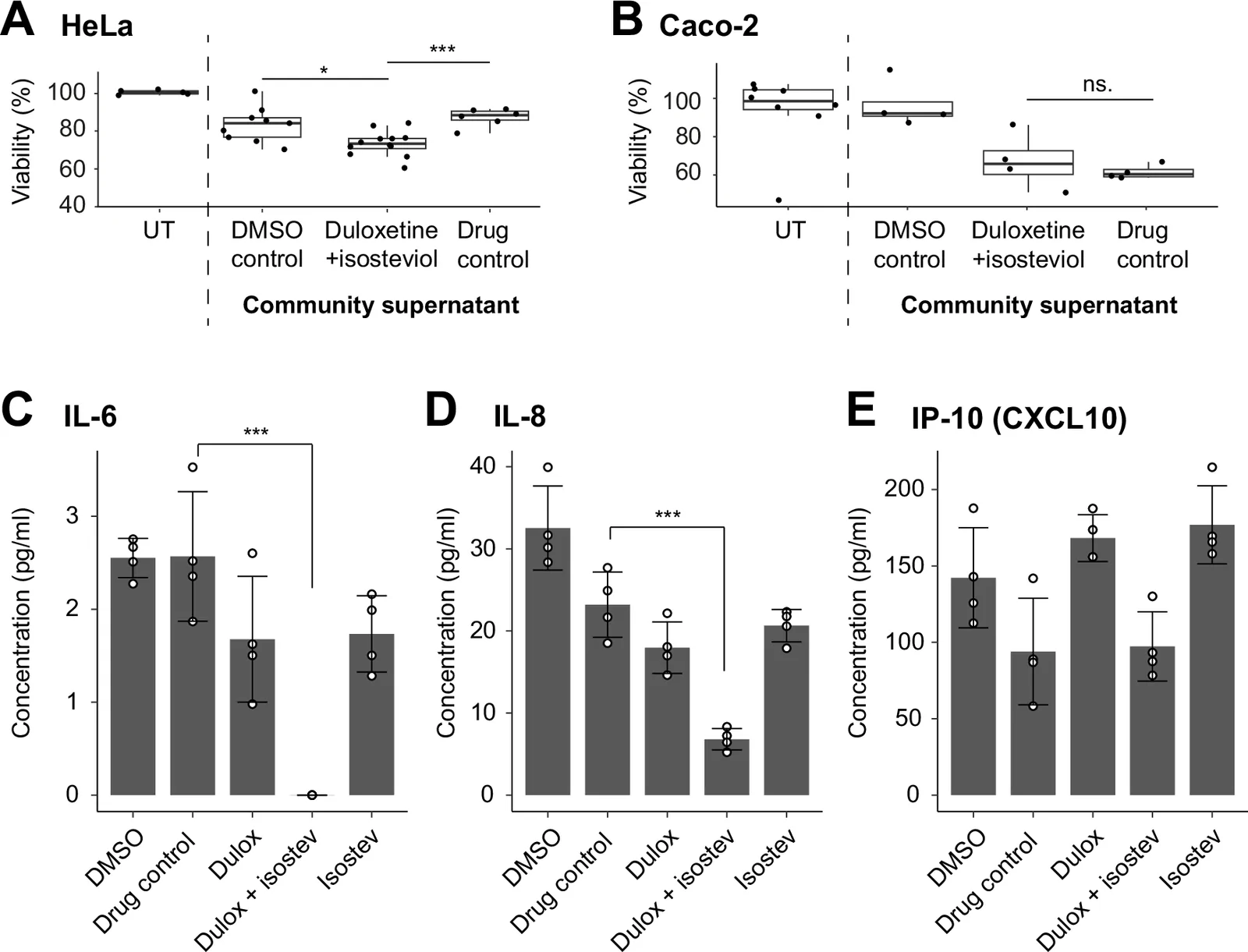
Metabolic response of gut bacterial community to isosteviol-duloxetine combination impacts cytotoxicity and cytokine secretion. (**A**) Spent medium of duloxetine and isosteviol co-treated communities show increased toxicity in HeLa cells (***P value: 0.000171281 and *P value: 0.01551908, box plot statistics: Q1 (25%): 75.1575, median: 84.165, Q3 (75%): 91.6075, IQR: 16.45, lower whiskers: 61.19, upper whiskers: 102.63, minimum: 61.19, maximum: 102.63), but not in (**B**) Caco-2 cells (*P* value: 0.482625095, box plot statistics: Q1 (25%): 65.140578675, median: 90.90481546, Q3 (75%): 98.57, IQR: 33.43, lower whiskers: 51.05, upper whiskers: 114.95, minimum: 51.05, maximum: 114.95). And secretion of the cytokines (**C**) interleukin-6 (IL-6, ***P value: 0.000321626), (**D**) interleukin-8 (IL-8, ***P value: 0.000228536), and (**E**) the interferon-γ inducible protein 10 kDa (CXCL10 or IP-10) by Caco-2 cells as a response to contact with spent medium. IL-6 and IL-8 secretion is significantly reduced in the supernatant of duloxetine and isosteviol-co-treated communities, as compared to the DMSO and drug control. The 25-strain bacterial community was treated with duloxetine (Dulox), isosteviol (Isostev), or their combination. Supernatants were harvested and applied to human cell lines. Controls included: (1) a DMSO-treated community (0.2% solvent control), and (2) supernatant from the DMSO-treated community supplemented post-harvest with 50 µM duloxetine and isosteviol (drug control). *N* > 6, significance was determined by Welch’s *t* test. *P value <0.05, ***P value <0.001, UT untreated cells. If not otherwise stated, error bars represent s.d. Source data are available online for this figure.

Following a 24-h incubation of HeLa cells with DMEM supplemented with 20% of community supernatant, we observed a significant increase in cytotoxicity in cells exposed to the supernatant derived from microbial communities treated with a combination of isosteviol and duloxetine, compared to the DMSO-only control (Fig. 6A; Datasets EV37 and EV38). To exclude direct effects of the compounds on HeLa cells, we included an additional control: supernatant from the DMSO-treated bacterial community supplemented with 50 µM isosteviol and duloxetine after harvesting and prior to application to human cell lines. Both the DMSO and the DMSO plus compounds control (drug control) were significantly less toxic than the supernatant of the community treated with the isosteviol-duloxetine combination (Fig. 6A).

To assess the impact of the altered bacterial secretome on gastrointestinal epithelial cells, we used colon carcinoma-derived Caco-2 cells. In addition to cytotoxicity, we also assessed the secretion of 13 cytokines. Cytokines are soluble proteins secreted by immune cells and intestinal epithelial cells (IEC) to mediate intercellular communication in homeostasis, but also play a relevant role during inflammation (Peterson and Artis, 2014; Andrews et al, 2018). IEC-derived cytokines such as interleukin-6 (IL-6), interleukin-8 (IL-8), and C-X-C motif chemokine ligand 10 (CXCL10, also known as IP-10), are key indicators of inflammation-associated damage in the gut in response to bacterial infection and Inflammatory Bowel Disease (Grimm et al, 1996; Korsten et al, 2022; Marion et al, 2004; Fusunyan et al, 1998). A reduction in these cytokines might indicate an anti-inflammatory response; however, it could also indicate the gut’s impaired ability to combat pathogens effectively (Shahini and Shahini, 2023; Østvik et al, 2013; Marchelletta et al, 2015).

While in Caco-2 cells no excess cytotoxicity was observed compared to the drug control (Fig. 6B; Appendix Fig. S21), the secretion of three cytokines was substantially modulated: IL-6, IL-8, and CXCL10. All three cytokines were stimulated by the supplementation of 20% community supernatant to the culture medium. The supernatant of a community co-treated with isosteviol and duloxetine (50 µM each) inhibited over 75% the secretion of IL-6 and IL-8 compared to the Community supernatant but did not affect that of IP-10 (Fig. 6C–E). IL-6 and IL-8 are key regulators of neutrophil biology: IL-6 promotes neutrophil production in the bone marrow, while IL-8 acts as a potent chemoattractant for neutrophils. Both cytokines exhibit both pro- and anti-inflammatory effects, with their roles depending on the specific biological context (Aliyu et al, 2022; Matsushima et al, 2022). They were previously shown to be stimulated by the presence of butyrate, LPS, IL-1beta, and TNFalpha (Fusunyan et al, 1998; Gasaly et al, 2021; Vitkus et al, 1998). The community supernatant of the 25-bacteria community contains ~3 mM butyric acid and 2 mM propionic acid. Both short-chained fatty acids (SCFA) are significantly reduced in cells treated with the supernatant of duloxetine and isosteviol-treated communities (Appendix Fig. S22). Although Caco-2 did not produce IL-1beta and TNFalpha (Dataset EV39), changes in SCFA production, in combination with microbial molecules such as LPS and flagellin, may contribute to IL-6 and IL-8 secretion by these cells. In support, we observed that the addition of LPS and a combination of propionate and butyrate, respectively, was sufficient to induce secretion of IL-8 and IP-10 (Appendix Fig. S23). These findings highlight a potential link between microbial metabolite modulation by sweetener–drug combinations and altered cellular inflammatory responses.

## Discussion

Previous cohort, animal, and in vitro microbiome studies have linked sweetener consumption with microbiome-level changes (Suez et al, 2014, 2022; Sun et al, 2022). Yet the roles of co-consumed compounds and mechanistic links to microbial activity remain largely unresolved. Our systematic mapping of xenobiotic–sweetener interactions provides mechanistic insights into sweetener–xenobiotic–microbe–host cell dynamics by revealing their effects on bacterial growth and metabolite secretion. As the latter is one of the main mediators of microbiome-host interactions (Shtossel et al, 2024; Zhang et al, 2023), it is likely that some of the reported effects of sweeteners and sweetener–xenobiotic combinations on the host are through changes in bacterial metabolites.

In the case of duloxetine and isosteviol, our study shows that their co-administration significantly alters the growth of several beneficial gut bacteria compared to either compound alone. This combination also impacts microbial community composition, metabolite secretion, cytotoxicity of microbial metabolites, and cytokine secretion by Caco-2 cells. Notably, it affects key microbiota-derived metabolites involved in host–microbiome interactions, such as butyrate and propionate. Within the human gastrointestinal tract, SCFAs generated by the gut microbiome are in situ utilized by colonocytes, with a minimal amount reaching the liver and even fewer entering the systemic circulation. Butyrate represents 15% of the gut bacteria-derived SCFAs and is used as a main source of energy by colonocytes (Hays et al, 2024). Meanwhile, propionate contributes to gluconeogenesis and lipid metabolism of the colonic cells (Liu et al, 2025), strengthening the gut barrier integrity. The decrease observed in these two microbial metabolites after treating the bacterial community with duloxetine and isosteviol may be linked to the suppressive effect on IL-8 and IL-6 secretion by Caco-2 cells.

While our data indicate that a combination of butyrate and propionate can stimulate IL-8 secretion, this alone does not fully account for the effects observed with the community supernatant (Appendix Fig. S23d). However, bacterial-derived butyrate influences the differentiation and inflammatory profile of mononuclear cells and colonic mucosal cells. In the absence of tissue damage, butyrate helps maintain the appropriate number of “patrolling monocytes” and inflammatory molecules, notably IL-6, IL-8, IL-1beta, and TNF, which are necessary to limit pathogen proliferation and invasion, thereby supporting intestinal integrity and repair in homeostasis (Hays et al, 2024). Conversely, propionate regulates the secretion of antimicrobial peptides by intestinal epithelial cells and the expression of cell receptors associated with IL-8 and CXCL10 secretion. IL-8 is a potent neutrophil chemoattractant that also mediates oxidative burst, both of which are crucial in immune responses against pathogens (Hays et al, 2024). Thus, the reduction of IL-6 and IL-8 induced by supernatants from communities treated with duloxetine and isosteviol indicates a potential dysregulation of inflammatory signaling that could impair host immune responses to pathogens. The gut functions as a sentinel organ under constant “alert” in basal conditions, requiring finely tuned regulation of complex interactions among intestinal epithelial cells, microbiota, metabolite production, cell receptor expression, and cytokine secretion (Mendes et al, 2019). Given the complex and dynamic interplay between the host inflammatory response and the gut microbiota, predicting the effects of these alterations remains challenging and requires validation in future in vivo studies. Nonetheless, since IL-6 and IL-8 play dual roles by exhibiting both pro- and anti-inflammatory effects depending on the context, the consequences of their modulation for host immunity will depend on the individual’s health status.

Emergent effects due to a mixture of compounds, the so-called ‘mixture effects’ or “mixture model”, is commonly considered in the context of drug efficacy and toxicity (Tyers and Wright, 2019). In the context of gut microbiota, antagonistic interactions have been linked to antimicrobial resistance (Brochado et al, 2018). However, emergent effects of chemical mixtures beyond antibiotic resistance remain sparsely studied and underappreciated. Our results provide a potential mechanism for some of the effects on inflammatory response previously described in pre-clinical studies of non-caloric sweeteners (Rosales-Gómez et al, 2018; Wang et al, 2014); and emphasize the need to study mixture effects more broadly, especially for food ingredients like sweeteners that are consumed daily by millions. Our multi-omics approach spanning genetic, proteomic, and metabolomic analyses underscores the need to study physiological effects beyond growth and provide a starting point to systematically unravel the complex sweetener–xenobiotic-microbiome-host interactions.

Multiple studies have examined the effects of long-term sweetener consumption across different stages of human life, reporting a broad range of impacts on both the host and gut microbiome (e.g., references (Meenatchi and Vellapandian, 2024; Pang et al, 2021a; Tsai et al, 2024; Laforest-Lapointe et al, 2021; Chi et al, 2018; Wu et al, 2025); cross-sectional studies and clinical trials reviewed in (Gauthier et al, 2024)). Some found significant links between sweetener consumption and obesity and cardiovascular disease (Santos et al, 2022; Chia et al, 2018; Laverty et al, 2015; Steffen et al, 2023; Azad et al, 2017). Future investigations will be necessary to elucidate how concurrently ingested food components, pharmaceuticals, and other bioactive compounds modulate the impact of sweeteners on the gut microbiome and host physiology.

Both of our experimental screens—one examining bacterium–sweetener interactions and the other assessing interactions among bacteria, sweeteners, and co-consumed compounds—were performed at a single concentration (50 µM for both sweeteners and co-consumed compounds) in a single growth medium (mGAM), using four co-consumed compounds. This controlled design allowed systematic interrogation of thousands of interactions but captures only a limited portion of the broader spectrum of dietary, pharmaceutical, and environmental exposures, as well as the considerable inter-individual variability in gut microbiota composition. Moreover, the choice of growth medium can influence microbial growth dynamics and interaction outcomes, potentially limiting the generalizability and sensitivity of in vitro findings (Castañeda-Monsalve et al, 2024). The species composition of the synthetic community further restricts interactions to those among the included taxa. Trophic interactions, such as cross-feeding, can modify species responses in communities relative to monocultures. Despite these limitations, synthetic communities provide mechanistic insights that are challenging to obtain in vivo, and previous work suggests that ~60% of monoculture effects are preserved in community contexts (Garcia-Santamarina et al, 2024). While human-derived microbiota are far more complex, their resolution is inherently limited by the methods used to characterize them (e.g., 16S rDNA vs. metagenomics sequencing), which can obscure finer-scale taxonomic and functional variation (Sun et al, 2022; Suez et al, 2022; Laforest-Lapointe et al, 2021). Thus, our screen represents only a limited subset of the possible compound–compound–microbe interactions. Nevertheless, it provides a valuable starting point for identifying higher-order interactions—potentially beneficial or deleterious—that warrant deeper investigation and should not be overlooked.

This study also highlights important directions for future research with potential public health relevance. Given the substantial inter-individual variability in gut microbiota composition at the strain level (Greenblum et al, 2015), it will be critical to determine whether the metabolic effects observed here are strain-specific. Such insights could inform personalized approaches to assess health risks originating from compound co-consumption as well as for microbiome-targeted diets and therapies. Furthermore, comprehensive in vivo profiling of the concentrations, kinetics, and dynamics of relevant compounds is essential to bridge the gap between in vitro findings and real-world exposures in human populations. These efforts will be key to evaluating the broader risks of microbiome–compound combination interactions for population health and consumer safety. Mechanistic insights from our study provide a starting point to unravel compound combination effects in the complex microbiome context.

## Methods

**Table:** Reagents and tools table

Reagent/resource	Reference or source	Identifier or catalog number
**Experimental models**		
*Agathobacter rectalis*	DSMZ	DSM 17629
*Akkermansia muciniphila*	DSMZ	DSM 22959
*Bacteroides fragilis (nontoxigenic)*	DSMZ	DSM 2151
*Bacteroides uniformis*	DSMZ	DSM 6597
*Bifidobacterium adolescentis*	DSMZ	DSM 20083
*Bifidobacterium longum subsp. infantis*	DSMZ	DSM 20088
*Clostridioides difficile*	DSMZ	DSM 27543
*Clostridium perfringens*	DSMZ	DSM 756
*Clostridium symbiosum*	BEI Resources	HM-309 (HMP ID 9474)
*Eggerthella lenta*	DSMZ	DSM 2243
*Enterocloster bolteae*	DSMZ	DSM 15670
*Escherichia coli ED1a*	INSERM	Denamur Lab, INSERM
*Escherichia coli IAI1*	INSERM	Denamur Lab, INSERM
*Fusobacterium nucleatum*	DSMZ	DSM 15643
*Lacrimispora saccharolytica*	DSMZ	DSM 2544
*Lacticaseibacillus paracasei*	Dupont Health and Nutrition	
*Lactobacillus gasseri*	DSMZ	DSM 20243
*Odoribacter splanchnicus*	DSMZ	DSM 20712
*Parabacteroides distasonis*	DSMZ	DSM 20701
*Parabacteroides merdae*	DSMZ	DSM 19495
*Phocaeicola vulgatus*	DSMZ	DSM 1447
*Roseburia intestinalis*	DSMZ	DSM 14610
*Segatella copri*	DSMZ	DSM 18205
*Streptococcus parasanguinis*	DSMZ	DSM 6778
*Streptococcus salivarius*	DSMZ	DSM 20560
**Recombinant DNA**		
**Antibodies**		
**Oligonucleotides and other sequence-based reagents**		
**Chemicals, enzymes, and other reagents**		**CAS number**
(4-Ethoxyphenyl)urea	TCI	150-69-6
5’-Adenylic acid	Selleck	61-19-8
Acesulfame potassium	Selleck	55589-62-3
Advantame	Sigma	714229-20-6
Allitol	Selleck	488-44-8
Aspartame	Selleck	22839-47-0
Caffeine	Sigma	58-08-2
Cyclamic acid	Selleck	100-88-9
D-allose	Sigma	2595-97-3
D-Tagatose	Selleck	87-81-0
D(-) Fructose	Sigma	57-48-7
DMSO	Sigma	67-68-5
Duloxetine	TCI	116539-59-4
Erythritol	Selleck	149-32-6
Glucose/Dextrose	Sigma	50-99-7
Glycyrrhizic acid	Sigma	1405-86-3
Iso-Steviol	Selleck	27975-19-5
L-Arabinitol	Selleck	7643-75-6
L-Gulose	Selleck	6027-89-0
Lactitol	Selleck	585-86-4
Lactose	Sigma	5989-81-1
Lactulose	Sigma	4618-18-2
Leucrose	Sigma	7158-70-5
Maltitol	Selleck	585-88-6
Maltose	Sigma	6363-53-7
Mannitol	Sigma	69-65-8
Mogroside V	Selleck	88901-36-4
Naringin Dihydrochalcone	Selleck	18916-17-1
Neohesperidin Dihydrochalcone (Nhdc)	Selleck	20702-77-6
Neotame	Sigma	165450-17-9
Nystose	Selleck	13133-07-8
Perillartine	Selleck	30950-27-7
Psicose	Sigma	551-68-8
Rebaudioside A	Selleck	58543-16-1
Rubusoside	Selleck	64849-39-4
Saccharin	Selleck	81-07-2
Sodium cyclamate	TCI	139-05-9
Sorbitol	Selleck	50-70-4
Stevioside	Selleck	57817-89-7
Sucralose	Selleck	56038-13-2
Sucrose	Sigma	57-50-1
Thaumatin	Sigma	53850-34-3
Trehalose	Sigma	6138-23-4
Vanillin	Sigma	121-33-5
Xylitol	Selleck	87-99-0
**Software**		
R		v4.4.1
GrowthCurver package	https://doi.org/10.1186/s12859-016-1016-7	
PEAR	https://doi.org/10.1093/bioinformatics/btt593	v0.9.11
DADA2	https://doi.org/10.1038/nmeth.3869	
QIIME 2	https://doi.org/10.1038/s41587-019-0209-9	v2018.04
CUTADAPT	https://doi.org/10.14806/ej.17.1.200	v1.10
MassHunter Workstation	Agilent	v10.1
**Other**		
Microplate Spectrophotometer	Agilent	BioTek Epoch 2
Microplate Stacker	Agilent	BioTek BioStack4
Biomek Automated Workstation	Beckman Coulter	i7
Anaerobic chamber	COY	Type B, vinyl
Illumina MiSeq	Illumina	

### Microbe selection and growth conditions

The 25 human gut microbial bacteria, 24 species and two different strains of *E. coli*, were selected based on abundance in the human gut and to cover a broad phylogenetic and metabolic diversity representative of the healthy microbiota, including also potential pathogens and probiotics (Dataset EV2) (Klünemann et al, 2021; Tramontano et al, 2018). Unless otherwise indicated, all bacteria were grown as liquid cultures in Gifu Anaerobic Medium broth, modified (mGAM, HyServe, Germany), an undefined rich medium that has been successfully used in cultivating gut microbial communities and individual species in previous screening studies (Maier et al, 2018; Klünemann et al, 2021). It has further been demonstrated to support the growth of most gut bacteria strains in monoculture (Tramontano et al, 2018). Culturing was carried out in a vinyl anaerobic chamber (COY) at 37 °C with 12% carbon dioxide, 86% nitrogen, and 2% hydrogen. All experimental cultures were started from the second passage culture after inoculation from a glycerol stock. All media, buffer, glass, and plasticware used in the study were exposed to the anaerobic conditions for at least 12 h before use.

### Plate preparation and screening of sweeteners and combinatory compounds

Screening was done as previously described in Müller et al and Maier et al (Müller et al, 2023; Maier et al, 2018). All tested sweeteners and compounds were dissolved in dimethyl sulfoxide (DMSO). The layout of screening plates can be found in Dataset EV3. Final screening concentration for all compounds was 50 µM, an amount estimated to reach the gut on average (Dataset EV4), in mGAM. The total screening volume used was 100 µL per well and the total exposure time was 24 h. The final concentration of the DMSO solvent was kept constant at 1% in both, single compound and combination screens. The starting OD_600_ was adjusted to 0.05 for all strains tested. Plates were sealed with breathable membranes (BreatheEasy®) to allow gas exchange. Optical density readings were taken at 600 nm, using the BioTek Epoch 2 Microplate Spectrophotometer and BioStack 4 Microplate Stacker (both from Agilent) fitted in a custom-made incubator system (EMBL mechanical workshop) inside the anaerobic chamber. The screen was carried out under anaerobic conditions in 96-well plates, and OD_600_ was measured every hour for 24 h. Each plate had six negative controls (bacteria with an equivalent amount of DMSO). Positive and negative effects of sweeteners on bacterial growth were assessed using respective negative controls on each plate. Each strain was screened at least three times (biological replicates) with two replicates per plate (technical replicates), adding up to 6 replicates in total.

Retesting and dose response were done as described above, in mGAM and 1% DMSO and growth was monitored hourly for 24 h. All 33 bacteria–sweetener interactions were retested in the sweetener concentrations 25, 50, 100, 200, and 400 µM. We regarded the retests as verified if two or more concentrations revealed to be significant (Welch’s *t* test, *N* > 3, *P* < 0.05, Datasets EV40 and41).

### Data processing and growth quantification

Optical density readings were analyzed by plate for each strain and replicate. Growth curves were fitted to each well using the “Growthcurver” package in R (Sprouffske and Wagner, 2016). The growth of each microbe in the presence or absence of compounds was quantified using the area under the curve (AUC) of the fitted growth equation. The entire screen was quality checked by overlaying all fitted and raw curves of technical and batch replicates for each species. Out of 39,313 screened samples, 492 wells showing technical errors and 136 failed fits (< 2% in total) were removed from further analysis, making a total of 38,685 samples entering further analysis (see Dataset EV5 for the complete dataset including the excluded wells).

### Data normalization and hit selection

Non-systematic variations were controlled by normalizing data within each plate to have comparable results across replicates. The wells were normalized by using 6 control wells present on each plate. Median percent inhibition (MPI) was used to normalize samples: MPI = sample AUC/ median (controls AUC). Welch’s *t* test was performed between all replicates (two technical and three biological replicates) of each sample per compound and their corresponding control wells. A two-sided *P* value at an α-level of 0.95, standardized effect sizes, and log2 of average fold changes were calculated. The *P* values were adjusted for multiple hypothesis correction using the “Benjamini–Hochberg” method. Also, log_2_ of average fold change ( = normalized AUCs) between technical replicates was reported for each batch replicate. Finally, the hit compounds were selected using two criteria: (1) *P* value < 0.05 and (2) log_2_FC of at least 20% (see Datasets EV5–12 for *P* values and single and combinatory screen details).

### Determination of combinatory interactions

To determine interactions between sweeteners with the combinatorial compounds the Bliss model of independence was used (Bliss, 1939), which divides interactions in synergistic, antagonistic, or no interaction. Here, we used the log-transformed normalized AUC as surviving fraction of microbes as developed by Demidenko et al (Demidenko and Miller, 2019). According to this model, two compounds (A and B) act independently, if the surviving fraction of cells upon simultaneous administration of both the compounds equals the product of compounds given separately. A synergy or an antagonism exists if SF_AB_ < S_A_S_B_ or SF_AB_ > S_A_S_B_ (Appendix Fig. S3a). A two-sided *P* value was computed for determining synergy or antagonism, and an interaction was seen as significant at a *P* value threshold of <0.05 and a synergy or antagonism of at least 20% as compared to the control.

### Synthetic microbial communities

Synthetic microbial communities were assembled from the 25 gut bacteria strains and species that were used in the growth screen in monoculture. Each bacterium was grown for two passages as described above and combined with all others in equal proportions, based on optical density under anaerobic conditions. The species mix was then diluted 1:20 with fresh mGAM medium alone or mGAM containing 50 µM of the compounds dissolved in DMSO (0.2% total DMSO, also in the negative control). Passaging was done every 24 h.

### 16S rDNA sequencing and data analysis

To ensure that separation on the species level is feasible, microbial species had been selected based on distinguishable V4 rDNA sequence prior to in vitro community assembly. All samples for community compositional analysis were examined by targeting the fourth hypervariable (V4) regions of the 16S rDNA gene as predicted by barrnap (Version 0.9). DNA was extracted using the MagMAX™ Microbiome Ultra Nucleic Acid Isolation Kit along with the KingFisher Flex Purification System. Library preparation for the 16S V4 was done by the European Molecular Biology Laboratory genomics core facility using a customized barcoding, amplification, and purification approach. The barcoded amplicons were pooled in equal concentrations and sequenced on the Illumina MiSeq platform using 2 × 250 base pairs at the EMBL genomic core facility in Heidelberg, Germany. Analysis of amplicon sequencing data was done as previously described in (Blasche et al, 2021).

### Sample preparation for proteomic analysis of *P. merdae* and *R. intestinalis*

Both species were inoculated from frozen stock cultures, three replicates each, into mGAM medium and passaged twice overnight. Passage two was then transferred into fresh medium (60 ml for *P. merdae* and 150 ml for *R. intestinalis*), and pre-exposure, time-point ‘0’, samples were collected directly hereafter (three per species, one sample per replicate, three in total). The second pre-exposure samples were collected upon reaching exponential growth (after three hours for *R. intestinalis* and 2.5 h for *P. merdae*), directly prior to splitting each replicate into four equal-sized batches, one per condition: Mock (DMSO, 0.2%), 50 µM duloxetine, 50 µM isosteviol, and a combination of both, 50 µM each. This resulted in 12 treatment samples, three per treatment, whereas each of the replicates could be traced back to one preculture. Bacteria were grown in the presence of the compounds at 37 °C for four hours under anaerobic conditions, before final sample collection. Pre-exposure samples were collected as controls to monitor changes in protein expression in different growth stages and thus to minimize the capturing of expression differences due to alterations in microbial growth (tested compounds affect species growth). For collection, samples were centrifuged at 4000 rpm for 25 min at 4 °C, 15 ml PBS was added to the pellet, followed by centrifugation at 4000 rpm for 10 min. Pellets were heat-inactivated in mass spectrometry-compatible lysis buffer containing 100 mM triethyl ammonium bicarbonate (TEAB) and 0.05% RapiGest (Waters, Wilmslow), twice by heating at 80 °C for 10 min followed by cooling for 10 min on ice. The inactivated cells were stored at −80 °C until further processing.

### Proteomics sample processing and data analysis

The heat-inactivated bacterial cells were lysed by sonication in 0.1% RapiGest in TEAB to release the proteins. Equal concentration of proteins from each sample was heat denatured, reduced with 4 mM dithiothreitol (DTT), alkylated with 14 mM iodoacetamide (IAA), and digested overnight with trypsin at 37 °C (1:50 protease:protein ratio). Equal concentration of the digested peptides was labeled with respective TMTpro 18 plex reagents at 1:10 peptide: label ratio in 100 mM TEAB pH 8.0 buffer in a randomized fashion across the samples to be compared. The labeling was carried out for 1 h, and the reactions were quenched with 0.4% hydroxylamine. Equal volumes of the labeled peptides were pooled to multiplex and vacuum dried. The multiplexed peptides were desalted using C18 spin columns (Pierce) as recommended by the manufacturer and subsequently fractionated offline by HpH reverse phase chromatography into 12 concatenated fractions. The fractions were dried and resuspended in 0.1% TFA in 3% Acetonitrile (ACN) for mass spectrometry.

An equal volume of peptides from each fraction was analyzed on an Orbitrap Eclipse™ mass spectrometer coupled in-line to an Ultimate 3000 RSLC™ nano LC system as described previously (Lindell et al, 2025). Reporter ion quantification was carried out from MS3 fragmentation spectra acquired employing a synchronous precursor selection (SPS) based on a real-time search (RTS) workflow. Uniprot protein fasta databases UP000004276 (unreviewed TrEMBL db downloaded on 20230718, contains 4351 seq) and UP000004828 (unreviewed TrEMBL db downloaded on 20220411; contains 4697 seq) were configured as databases for RTS for *P. merdae* and *R. intestinalis* datasets, respectively.

The raw data were imported as fractions into Proteome Discoverer 3.0 and analyzed against respective protein databases using a predefined workflow employing both SequestHT and Comet search algorithms as described previously (Lindell et al, 2025). In brief, the search parameters included mass tolerance up to 10 ppm for precursors and 0.6 Da, up to two missed cleavages, TMT labeling on Lys and N-terminus and Carbamidomethylation of Cys as fixed modifications, and oxidation of Met as a variable modification. Common contaminants matching to the cRAP database from GPM were filtered out from the search results before empirical analysis.

Matches to the decoy database were utilized for False Discovery Rate (FDR) validation employing Percolator, and matches passing 1% FDR threshold were taken further for defining qualitative and quantitative identification. Quantitative comparisons and statistical analyses were performed using Proteome Discoverer workflows. The S/N values of TMT reporter ions of the identified peptides, which are unique to the representative proteins, were used to represent the summed protein abundance. The TMT channel with the highest total protein abundance was used as the reference channel for normalization. The normalized abundance values were used for quantitative comparison across sample sets. Protein ratios were calculated from normalized grouped abundances, comparing test and control groups. Fold changes were represented by the median abundance across replicates. Statistical significance was assessed using ANOVA at the individual protein level, with adjusted *P* values computed using the Benjamini–Hochberg correction to control the false discovery rate in this large-scale analysis.

### Tn-Bar-seq with *P. merdae* genome-wide transposon library

Two vials of the pooled library of *Parabacteroides merdae* ATCC 43184 mapped to GenBank: CP102286.1 (Voogdt et al, 2025) were thawed and used to inoculate mGAM with 30 μg/mL erythromycin and grown until mid-exponential phase. At this stage, the culture was used to inoculate at OD_600_ 0.02, 1 mL assay cultures in a deep well plate, with mGAM containing 50 μM of duloxetine, isosteviol alone or in combination in duplicates and triplicate for the DMSO control. The libraries were grown until the early stationary phase at 37 °C in an anaerobic chamber, where endpoint samples were taken for gDNA extraction. The growth of the mutant library was monitored by growing in parallel a 100 μl aliquot of the experiment and measuring OD_600_ in the same set up than the initial growth screen (BioTek Epoch 2 Microplate Spectrophotometer and BioStack 4 Microplate Stacker). gDNA was extracted with MagMAX Microbiome Ultra Nucleic Acid Isolation Kit, and the barcodes amplified with NEB Q5 Hot Start HF X2 with custom Tn-Bar-Seq primers. The amplicon with the barcodes was sequenced with Illumina NextSeq 75 nt SE. Barcodes were counted with 2FAST2Q (Bravo et al, 2022), and changes in mutant gene abundance were analyzed with TRANSIT using reads in the central 80% of the open reading frame and normalized by Trimmed Total Read-count and compared to the DMSO control. *P* values were calculated with TRANSIT resampling set at 20,000 permutations and adjusted for multiple comparisons with the Benjamini–Hochberg procedure (DeJesus et al, 2015). To cross-compare Tn-Bar-Seq to proteomics, annotations were matched by BlastP best reciprocal hit, Protein Accession to Locus ID match can be found at (Dataset EV15).

### Bioaccumulation of duloxetine and isosteviol

Bacteria were inoculated from the second passage at a starting OD_600_ of 0.05 and grown anaerobically at 37 °C in the presence of the compounds and their combination. Controls, the solvent control (bacteria plus DMSO) and the incubation control (bacteria-free mGAM with the compounds), were treated the same way. The incubation control tests for bacteria-independent compound degradation and, when compared to the whole culture, may reveal compound metabolism if a significant amount is metabolized within the observed time frame. After 24 h the sampled were taken out and whole culture (wc, no further treatment), supernatant (sup, centrifuged at 4000× *g* for 25 min) and compound control were collected and stored at −80 °C until extraction.

### Sample extraction for untargeted metabolite analysis and compound bioaccumulation

LC-MS samples, both whole cultures and supernatants, were extracted by adding 4x the amount of ice-cold (−20 °C) extraction buffer (1:1 ratio of methanol and acetonitrile with internal standards, 75 µM caffeine and 60 µM ibuprofen, spiked in) followed by an incubation at 4 °C for 15 min. Samples were then centrifuged at 4 °C and 4000 rpm for 25 min and supernatants were transferred to 96-well plates for LC-MS analysis. Samples for concentration calibration (1 in 2 dilution of compounds) and bacteria-free compound controls were processed in the same way.

### Reverse-phase LC-MS for untargeted metabolite analysis and compound bioaccumulation

LC-MS analysis was performed on an Agilent 1290 Infinity II LC system coupled with an Agilent 6546 Q-TOF with JetStream ESI source. The separation was performed using a ZORBAX RRHD Eclipse Plus column (C18, 2.1 × 100 mm, 1.8 μm; Agilent 858700-902) with a ZOBRAX Eclipse Plus guard column (C18, 2.1 × 5 mm, 1.8 μm; Agilent 821725-901) at 40 °C. The multisampler was kept at a temperature of 4 °C. The injection volume was 1 μL, and the flow rate was 0.4 mL/min. The mobile phases consisted of A: water + 0.1% formic acid + 5 mM ammonium formate; B: methanol + 0.1% formic acid + 5 mM ammonium formate. The 10 min gradient started with 5% solvent B, which was increased to 90% by 5 min and then further increased to 100% by 7 min, before returning to 5% solvent B for a 3 min re-equilibration. The instrument was operated in TOF MS acquisition mode in either positive or negative polarity. (30-1500 m/z). The source parameters were as follows: gas temperature: 200 °C, drying gas: 9 L/min, nebulizer: 20 psi, sheath gas temperature: 400 °C, sheath gas flow: 12 L/min, VCap: 3000 V, nozzle voltage: 0 V, fragmentor: 110 V, skimmer 45 V, Oct RF Vpp: 750 V. The online mass calibration was performed using a reference solution (positive: 121.05 and 922.01 *m/z*; negative: 112.99 and 1033.99 *m/z*). Collision energies used were 0 V, 10 V, 20 V, 40 V. Compounds of interest were identified based on their retention time, accurate mass, and fragmentation patterns, using pure standards for method development, compound identification, and calibration.

Untargeted metabolite analysis was performed using the Agilent MassHunter Workstation Profinder 10.0 software. Area under the curve was extracted, using the Batch Recursive Feature Extraction function for small molecules and peptides. Retention time was restricted from 0.5 to 8.0 min, and a tolerance was set to 0.05 min. Peaks above a raw height of 4000 in at least one sample were extracted, and peaks below 4000 in all samples were regarded as noise and discarded.

### Untargeted metabolomics peak identification

Raw data files were converted to.mzML format using msconvert (ProteoWizard) with peakPicking and zeroSamples functions applied. The.mzML files were uploaded to R, and XCMS was used to perform peak picking, refinement, correspondence, and peak filling with the parameters listed in Dataset EV42 (Smith et al, 2006). Statistical analysis was performed using MetaboAnalyst 6.0 (Pang et al, 2021b). Near-constant variables were filtered by interquartile range, so 25% of features were removed, and the data were then normalized by sum, log-transformed, and Pareto scaled. Correlation analysis was performed using the PatternHunter tool to highlight features more significantly altered by combination treatment with both duloxetine and isosteviol than by either treatment alone. The correspondence of feature intensity to this pattern was determined using Pearson correlation. A significance threshold of a correlation coefficient > ± 0.8 and *P* value < 0.05 was employed. Metabolite identification was performed by comparison of accurate mass to theoretical mass with an error tolerance of ±10 ppm and where possible by matching of MS2 spectra to database MS2 spectra using the PEP Search tool (Tang et al, 2014).

Targeted metabolomics samples were prepared by adding 120 μl of extraction buffer (1:1 methanol:acetonitrile, 0.1% formic acid, 20 μM caffeine, amoxicillin, and donepezil as internal standards) to 80 μl of sample, followed by a 30 min incubation at 4 °C and centrifugation (5 min, 4 °C, 3200× *g*) to remove precipitated proteins and salts. For amino acid and organic acids (except SCFA) analysis, samples were diluted 1:10 in water prior to extraction. Analysis was performed on an Agilent 1290 Infinity II LC system coupled with an Agilent 6470 triple quadrupole mass spectrometer with JetStream ESI source operated in dynamic multiple reaction monitoring (dMRM) mode. Amino acids were measured using a low pH HILIC as described previously (Mülleder et al, 2016). In brief, 0.25 μl of sample was injected, and analytes were separated on a Waters Acquity BEH Amide column (1.7 µm, 2.1 mm × 100 mm) maintained at 35 °C. The gradient used a constant flow rate of 0.9 ml/min and proceeded as follows: 0 min: 15% buffer A (1:1 acetonitrile:water, 10 mM ammonium formate, 0.176% formic acid) and 85% buffer B (95:5:5 acetonitrile:methanol:water, 10 mM ammonium formate, 0.176% formic acid), 0.7 min: 85% B, 2.55 min: 5% B, 2.9 min: 5% B, 2.91 min: 85% B, 3.5 min: stop time.

Organic acids were measured using a high pH HILIC method as described previously (Kamrad et al, 2020). In brief, 2 μl of sample was injected, and analytes were separated on a Waters Atlantis Premier BEH Z-HILIC (1.7 µm, 2.1 mm × 100 mm) column maintained at 35 °C. The gradient used a constant flow rate of 0.5 ml/min and proceeded as follows: 0 min: 15% buffer A (15 mM ammonium bicarbonate in water, pH 9) and 85% buffer B (15 mM ammonium bicarbonate in 9:1 acetonitrile:water, pH 9), 1 min: 85% B, 3 min: 25% B, 4 min: 25% B, 4.01 min: 85% B, 5 min: stop time.

SCFA were measured using a porous graphitic carbon column as described previously (Saha et al, 2021). In brief, 1 μl of sample was injected, and analytes were separated on a Thermo Scientific Hypercarb PGC column (3 µm, 50 mm × 2.1 mm) maintained at 40 °C. The gradient used a constant flow rate of 0.15 ml/min and proceeded as follows: 0 min: 100% buffer A (water, 0.1% formic acid) and 0% buffer B (acetonitrile, 0.1% formic acid), 4 min: 60% B, 4.1 min: 100% B, 6 min: 100% B, 6.01 min: 0% B, 9 min: stop time.

A pooled QC sample, blanks, and serial dilutions of a mixed analytical standard were injected throughout each run. Data were analyzed using MassHunter Workstation Quantitative Analysis for QQQ v10.1. Concentrations were obtained via external calibration using standard curves.

### Cytotoxicity assay

HeLa and Caco-2 cell lines (human cell lines, passage numbers 10 and 15, respectively) were obtained from the Cambridge University Biological Services Unit, authenticated by ATCC, and tested for Mycoplasma before use. The cells were cultured in DMEM (Gibco), containing 10% Fetal Bovine Serum (FBS; Sigma-Aldrich) in standard conditions (37 °C, 5% CO_2_). Cells were trypsinized and seeded in 96-well plates at a density of 2.5 × 10^4^/well, in 80 μL of phenol-red and FBS-free media, allowed to adhere overnight, and treated by adding 20 μL of community spent media for 24 h. Untreated cells were used as a negative control, taken as 100% viability. Further negative controls include supernatant of DMSO-treated microbial community with (DMSO control) and with drugs added prior to cell culture exposure (drug control), see Fig. 6A,B. All microbial supernatants were centrifuged and sterile filtered (Millex PVDF syringe filter, pore size 0.22 μm, Merck) prior to application. 5 μM staurosporine was used as a positive control for cytotoxicity induction. Cytotoxicity was assessed by adding 20 μL of CellTiter-Blue Reagent (Resazurin) (Promega) and incubating at 37 °C, 5% CO_2_ for 3 h. Fluorescence was measured using a VarioSkan (Thermo) plate reader, at 560/590 nm.

### Cytokine assay

Caco-2 cells were seeded and treated as described for the cytotoxicity assay. After 24 h of treatment, supernatants were harvested and stored at -80 °C until use. For cytokine assessment, LEGENDplex^TM^ Human Essential Immune Response Panel (13-plex) (BioLegend Cat. No. 740930) was used. In brief, 25 μL of the supernatant was mixed with capture beads coated with antibodies specific to every target cytokine. After 2 h incubation at RT, samples were washed, and 25 μL of detection biotinylated antibodies were added and incubated for 1 h at RT. Finally, 25 μL of streptavidin-PE conjugated was added to every sample and washed after 30 min of RT incubation. A BD LSRFortessa^TM^ was used for data acquisition using the settings specified by the supplier. Data was analyzed using BioLegend’s LEGENDplex™ data analysis software.

### Supernatant titration for HeLa cytotoxicity assay

To determine what proportion of cell culture medium (DMEM) can be replaced by bacterial community supernatant, we tested three percentiles of supernatant in HeLa cells: 5, 10, and 20%. Although the supernatant decreased cell viability with increasing concentration, even the highest concentration of 20% spent medium revealed a cell viability of 84%, high enough to be suitable for testing (Appendix Fig. S24).

### Staurosporine and LPS titration

The amount of staurosporine to be added for apoptosis induction was determined for HeLa and Caco-2 by titration (Appendix Figs. S25 and S26). Induction of cytokines by staurosporine and LPS was titrated in Caco-2 cells. The amount of staurosporine used to induce apoptosis (> 4 µM) is higher than the one inducing cytokine secretion (Appendix Fig. S23).

#### Replicates and statistical tests

Technical replicates refer to replicates within the same batch (for example, two bacterial cultures inoculated from the same starting culture or on the same plate), whereas biological replicates refer to independent experiments. All indicated sample numbers (or displayed data points) refer to distinct samples (biological and technical replicates) and not to repeated measurements of the same samples. All statistical tests are two-sided. FDR correction was applied using the Benjamini–Hochberg procedure. Unless otherwise specified, statistical significance was assessed using Student’s *t* test. Error bars represent standard deviation (s.d.).

#### Blinding

No blinding was done in experimental work or analysis.

## Supplementary information

## Data Availability

The datasets and computer code produced in this study are available in the following databases: Proteomics for *Roseburia intestinalis* and *Parabacteroides merdae:* PRIDE PXD045140. Codes used for processing and raw screening data: GitHub (https://github.com/periwal45/SW_SF100). Tn-Bar-Sequencing: ENA PRJEB76554. 16S amplicon sequencing: ENA PRJEB76553. Metabolomics: MetaboLights MTBLS11480. The source data of this paper are collected in the following database record: biostudies:S-SCDT-10_1038-S44320-026-00225-6.
