# Supplementary material for: Common xenobiotics modulate gut microbial responses to low‑calorie sweeteners in vitro
Source: Mol Syst Biol. 2026 Jun 25;22(9):1453–73. doi: 10.1038/s44320-026-00225-6 (PMC13538661; doi:10.1038/s44320-026-00225-6)
Supplement: Supplementary file 1 [file 44320_2026_225_MOESM1_ESM.pdf]

Appendix for '**Common xenobiotics modulate gut microbial responses to low-calorie sweeteners *in vitro***'

Table of content

Appendix Figure S1 3
Selection of hits
Appendix Figure S2 4
Sweetener screen and retest
Appendix Figure S3 5
Bliss interaction
Appendix Figure S4 7
Commonly used drugs and sweeteners tested for combinatory interactions in the 25 gut bacteria
Appendix Figure S5 8
Community stability over passages
Appendix Figure S6 9
Relative abundance per species in DMSO and isosteviol-duloxetine
Appendix Figure S7 10
Growth inhibition of *Parabacteroides merdae* by duloxetine, isosteviol and a combination of both as compared to the compound solvent DMSO (1%) at a concentration of 50 µM each.
Appendix Figure S8 11
Differences in protein expression in *Roseburia intestinalis* and *Parabacteroides merdae* in different growth stages after culture inoculation.
Appendix Figure S9 12
The effect of the combined treatment with duloxetine and isosteviol (y-axis) differs from the addition of the effect of each of the individual compounds in both, *P. merdae* and *R. intestinalis.*
Appendix Figure S10 13
Supplements tested for their effect on the synergy between isosteviol and duloxetine.
Appendix Figure S11 15
Suppl. for *P. merdae* BarSeq library
Appendix Figure S12 17
Supplementation of *P. merade* with magnesium sulfate (MgSO4) at different concentrations. MgSO4 did not alter growth up to a concentration of 240 mg/L in mGAM medium.
Appendix Figure S13 18
Bioaccumulation of duloxetine and isosteviol
Appendix Figure S14 19
Altered metabolite production and secretion in S. copri, R. intestinalis and P. merdae upon overnight exposure to duloxetine, isosteviol and the combination
Appendix Figure S15 20
Metabolites affected from combined exposure to duloxetine and isosteviol of *R. intestinalis*, *P. merdae* and *S. copri*.
Appendix Figure S16 21
Changes in the concentrations of 32 amino acids and derivates in *Segatella copri* upon overnight exposure to duloxetine, isosteviol and the combination.
Appendix Figure S17 22
Changes in the concentrations of organic acids in *Segatella copri*, *Roseburia intestinalis* and *Parabacteroides merdae* upon overnight exposure to duloxetine, isosteviol and the combination.

Appendix Figure S18 .... **24**
Changes in the concentrations of short chain fatty acids in *Segatella copri*, *Roseburia intestinalis* and *Parabacteroides merdae* upon overnight exposure to duloxetine, isosteviol and the combination.

Appendix Figure S19 .... **25**
Changes in the concentrations of 32 amino acids and derivates in *Roseburia intestinalis* upon overnight exposure to duloxetine, isosteviol and the combination.

Appendix Figure S20 .... **26**
Changes in the concentrations of 32 amino acids and derivates in *Parabacteroides merdae* upon overnight exposure to duloxetine, isosteviol and the combination.

Appendix Figure S21...**27**
Secretion of the cytokines interleukin-6 (IL-6), interleukin-8 (IL-8) and the interferon-γ inducible protein 10 kDa (CXCL10) by Caco-2 cells as a response to contact with spent medium.

Appendix Figure S22 .... **28**
Concentration of the five short chain fatty acids in the supernatant of the 25 bacteria community.

Appendix Figure S23 .... **29**
Secretion of IL-6, IL-8 and IP-10 after exposure to different concentrations of staurosporine and LPS in Caco-2 cells and induction of cytokine secretion after treatment with 3 mM butyrate + 2 mM propionic acid.

Appendix Figure S24 .... **30**
Viability test for HeLa cells exposed to 5, 10 and 20 % spent media from the 25 species community, done in triplicates.

Appendix Figure S25 .... **31**
HeLa cell viability after treatment with different concentrations of staurosporine for apoptosis induction.

Appendix Figure S26 .... **32**
Caco-2 viability after treatment with different concentrations of staurosporine for apoptosis induction.

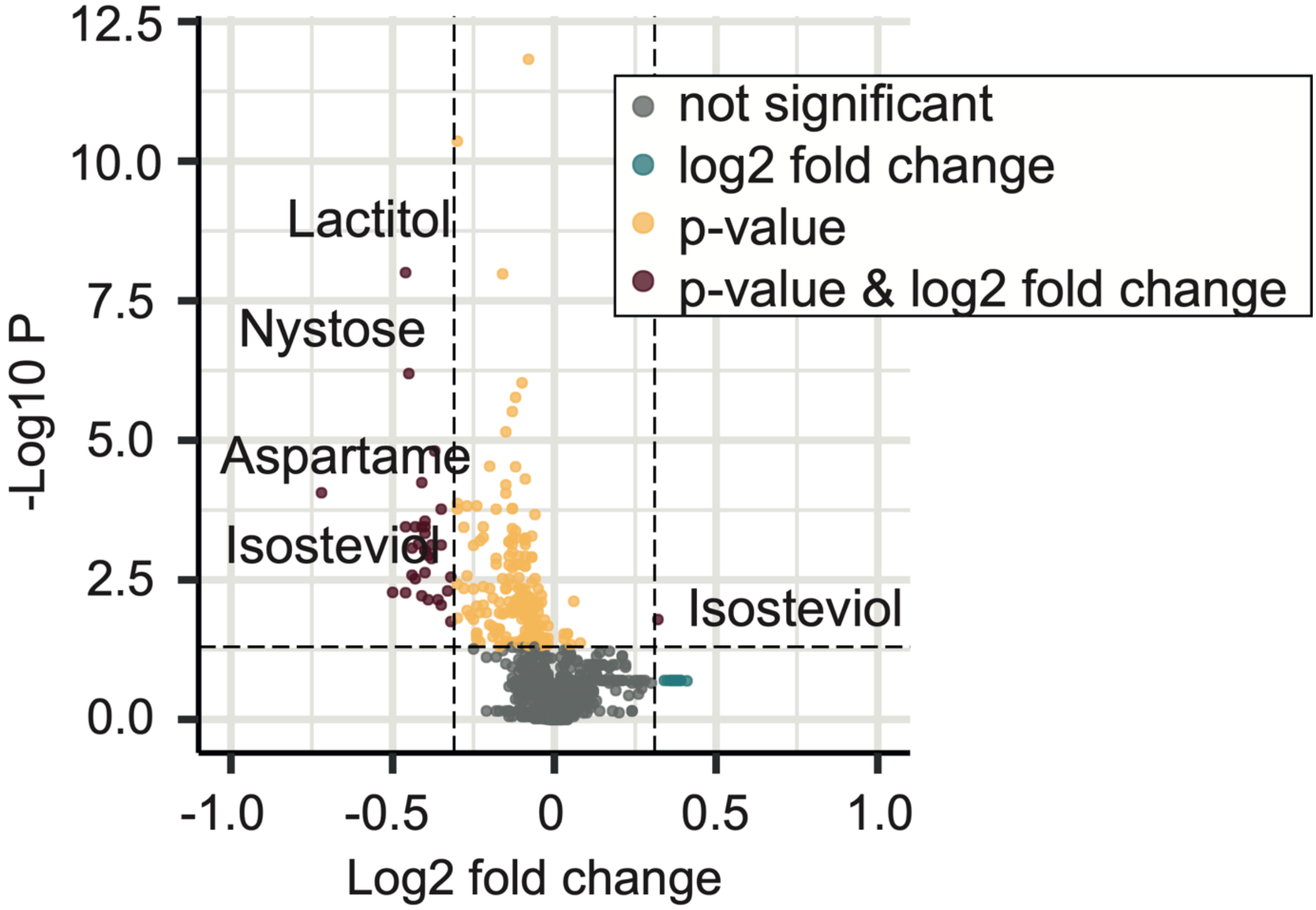

Appendix Figure S1. Selection of hits – normalised area under the curve (AUC) across all replicates was pooled and p-value between control and treated wells was computed (Welch's t-test). Median of the log2 fold change of all replicates was computed. Significant hits were determined by setting a threshold of p-value at a confidence interval of 95% and log2 fold change of +/- 0.32 (20% difference in growth)

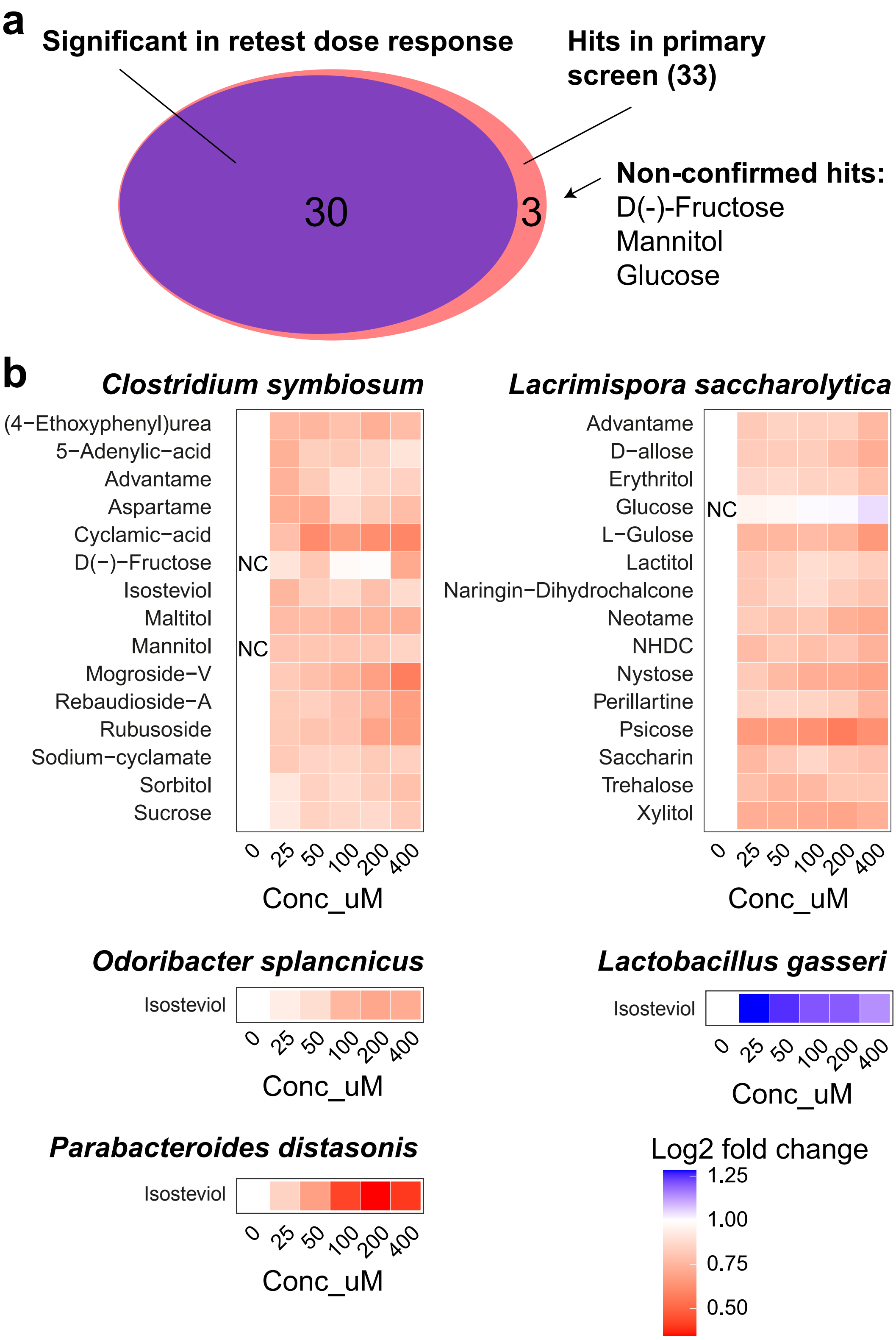

Appendix Figure S2. (a) Venn diagram showing the significant hits in the original sweetener-bacteria screen and the retested dose response. (b) Results of the dose response of the retested sweetener screen hits, concentrations tested: 0, 25, 50, 100, 200 and 400 μM. NC = not significant (criteria: min. two conc. significant in $p<0.05$, N>=3)

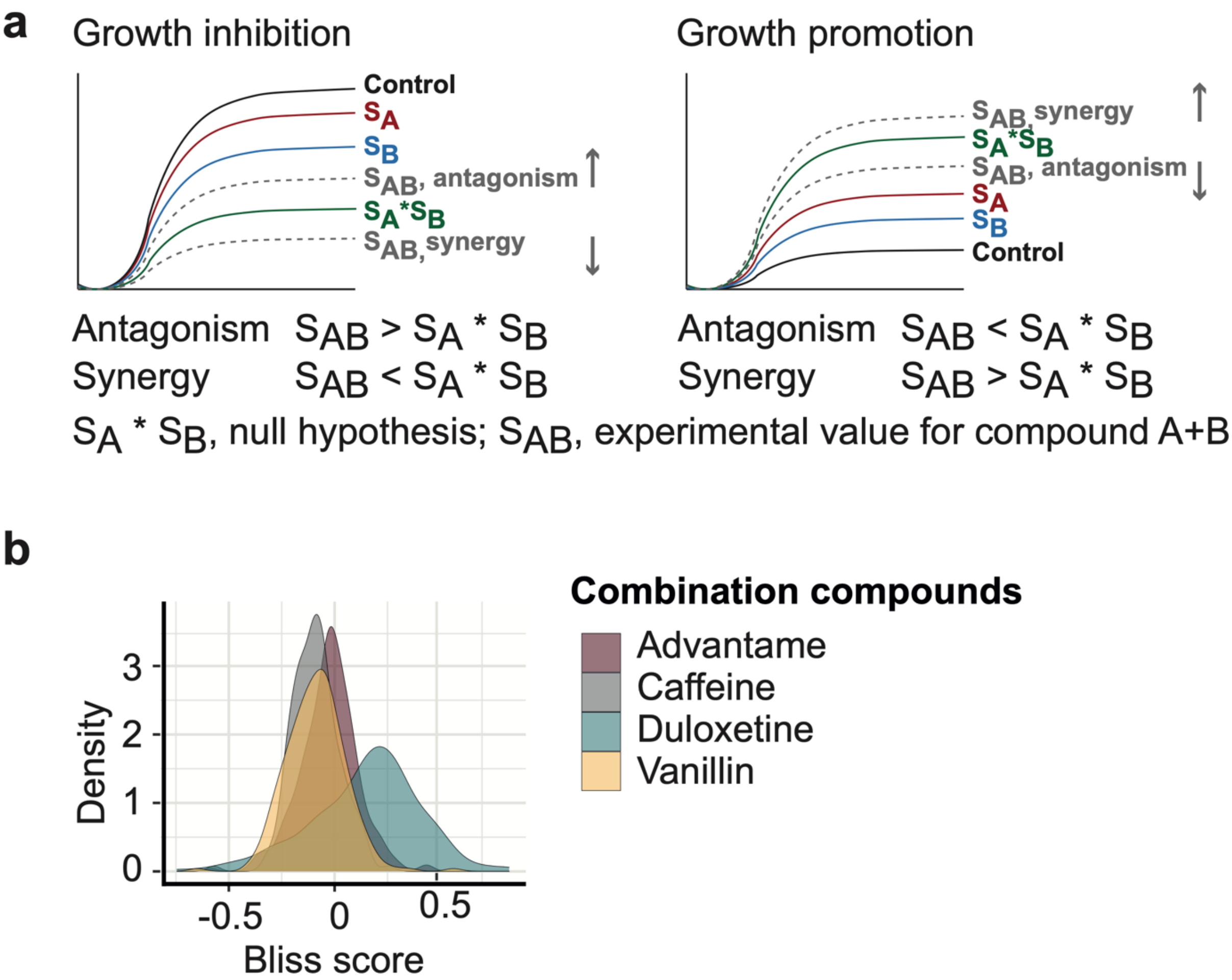

Appendix Figure S3. (a) Definition of synergy and antagonism in growth inhibition and growth promotion scenario. To determine interactions between sweeteners with the combinatorial compounds we used the bliss model of independence (BLISS, 1939) which assesses whether an interaction is synergistic or antagonistic or no interaction. We used log transformed controls normalized AUC values as surviving fraction (SF) of microbes to test the bliss independence as reported in Demidenko et al., log transformation turns the bliss model into a linear equation that can be tested easily by standard statistical tests (Demidenko & Miller, 2019). According to bliss model of independence, two compounds (A and B) act independently (eqn. 2) if the surviving fraction of cells upon simultaneous administration of both the compounds (i.e., SF_{AB}) equals the product of compounds given separately (SF_A and SF_B). A synergy exists if $SF_{AB} < S_A S_B$ else it's an antagonism (i.e., $SF_{AB} > S_A S_B$).

$$SF_{AB} = SF_A * SF_B \quad \text{eqn. 2}$$

As explained in Demidenko et al., log transformed surviving fractions (from eqn. 2) can then be expressed as the null hypothesis (H_0, eqn. 3) suggesting no interaction which can then be statistically tested for alternate hypothesis (H_A, eqn. 4) by using the standard *t*-distribution with 3 degrees of freedom (eqn. 5).

$$H_0 : lnSF_A + lnSF_B - lnSF_{AB} = 0 \quad \text{eqn. 3}$$

$$H_A : lnSF_A + lnSF_B - lnSF_{AB} \neq 0 \quad \text{eqn. 4}$$

$$T = \frac{(\tilde{x}_1+\tilde{x}_2-\tilde{x}_3)\sqrt{\sum_{i=1}^{3} n_i-3}}{\sqrt{\sum_{i=1}^{3}\sum_{j=1}^{n_i}(x_{ij}-\tilde{x}_i)^2\sum_{i=1}^{3} n_i^{-1}}} \quad \text{eqn. 5}$$

Where $\tilde{x}_1$, $\tilde{x}_2$ and $\tilde{x}_3$ represents mean log SF of the three groups (A, B and AB), n_i represents number of replicates in *i*th group and *j* is the group. A two-sided p-value is computed for determining synergy or antagonism. An interaction is pronounced significant at p-value threshold of < 0.05 and a synergy or antagonism of at-least 20%.

(b) Synergistic and antagonistic interactions of sweeteners combined with four commonly used co-occurring compounds.

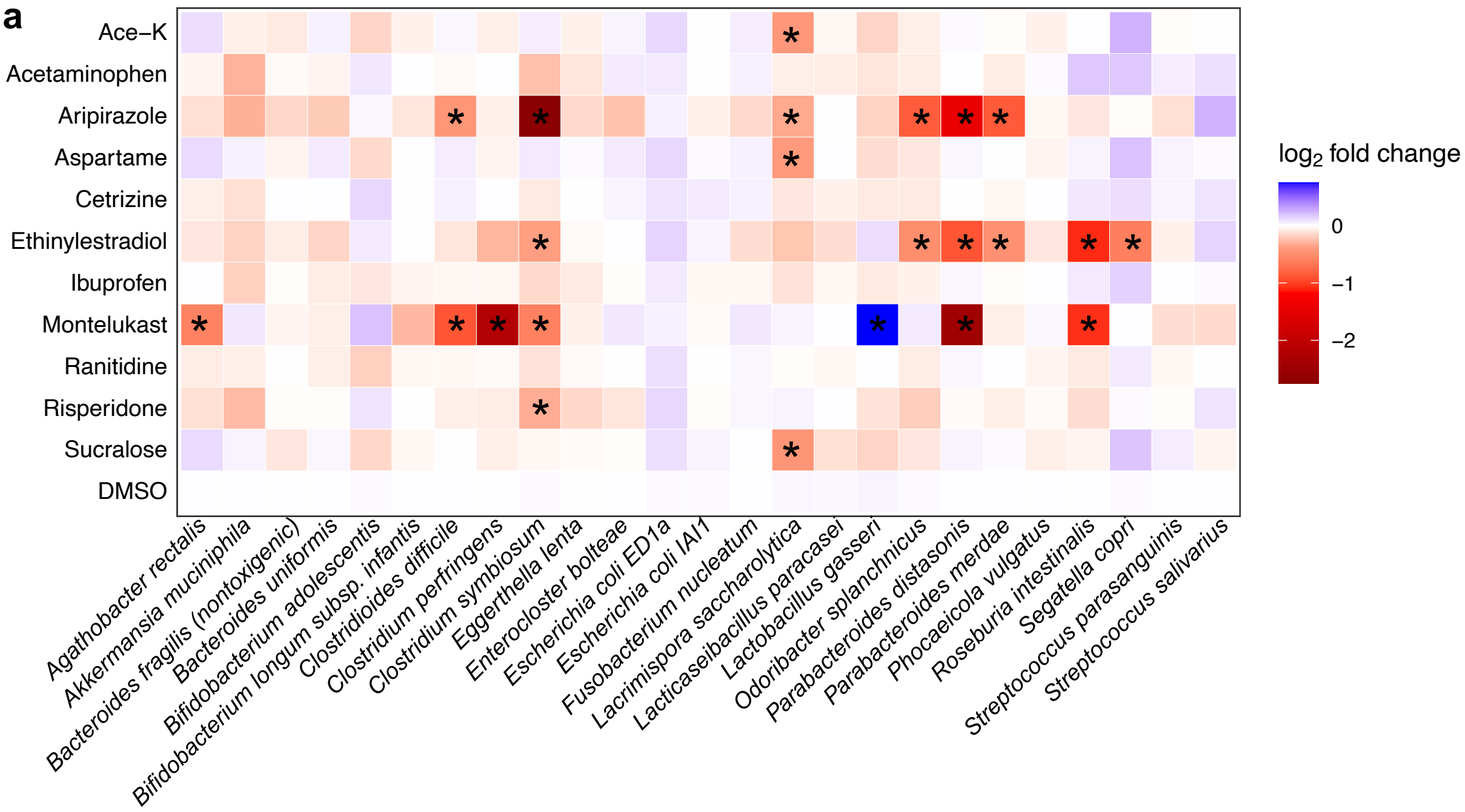

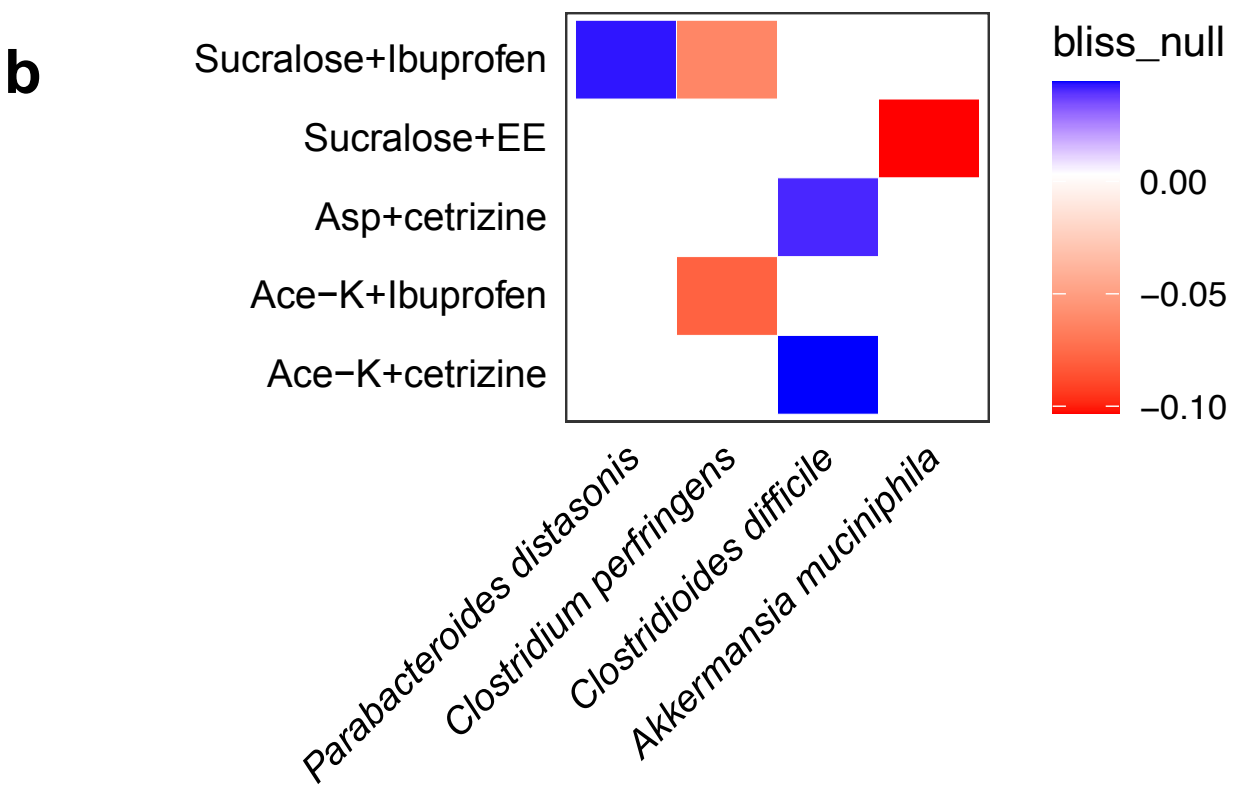

Appendix Figure S4. (a) Effect of commonly used drugs and sweeteners on microbial species (* $p<0.05$) and (b) significant bliss scores of these drugs combined with commonly used sweeteners added to the drug formulation ($p<0.05$).

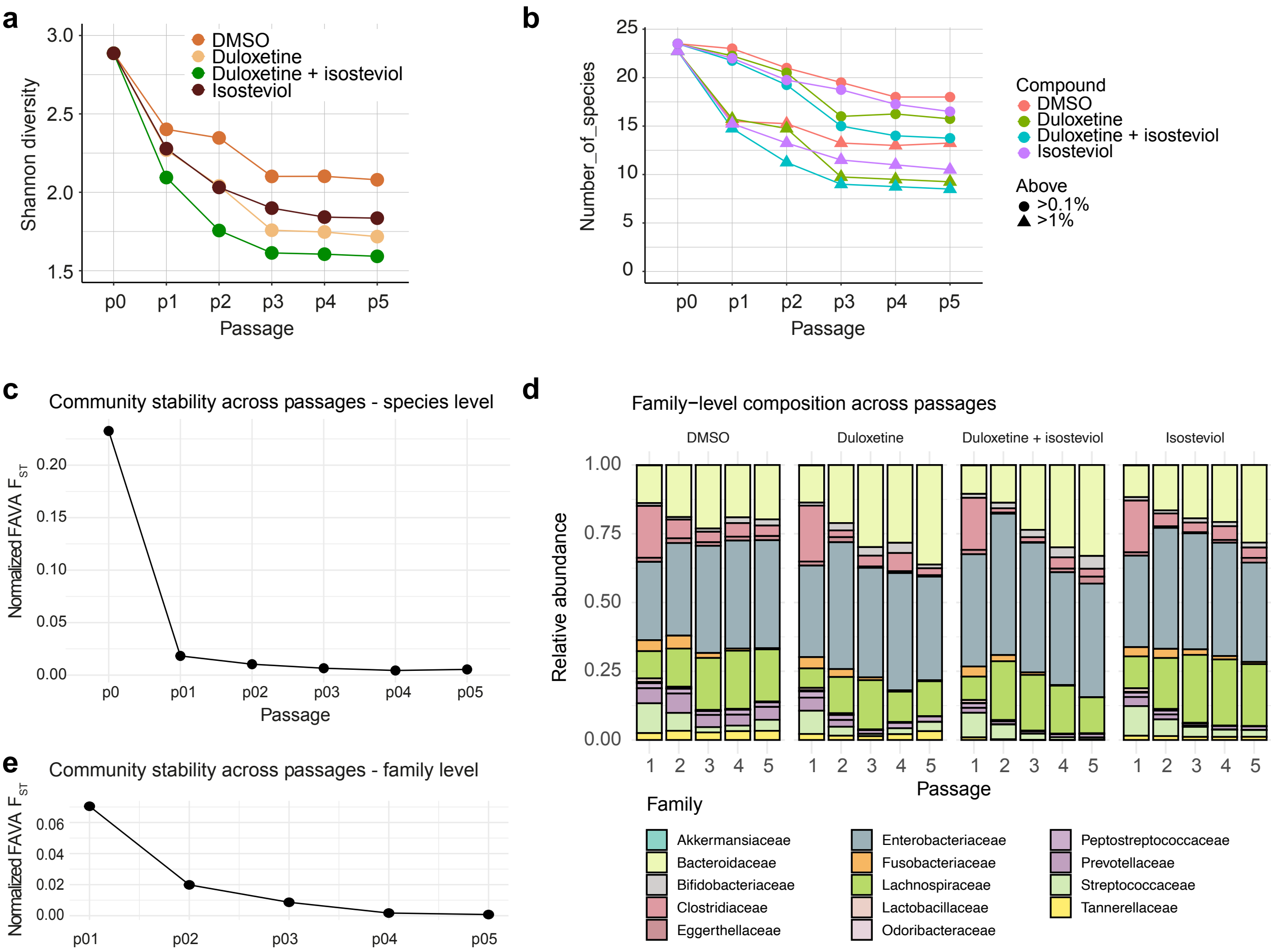

Appendix Figure S5. Community stability over passages a) Changes in alpha diversity throughout the passages in compound-treated communities (species level, cutoff 10 reads per species in amplicon sequencing), b) number of species detected when discounting low abundant species. c) Community stability as assessed by FAVA, a tool for evaluating temporal stability of microbial communities on species level (Morrison *et al*, 2024). d) Family level proportions of the bacterial composition of the communities at different transfers and e) community stability as assessed by FAVA on family level.

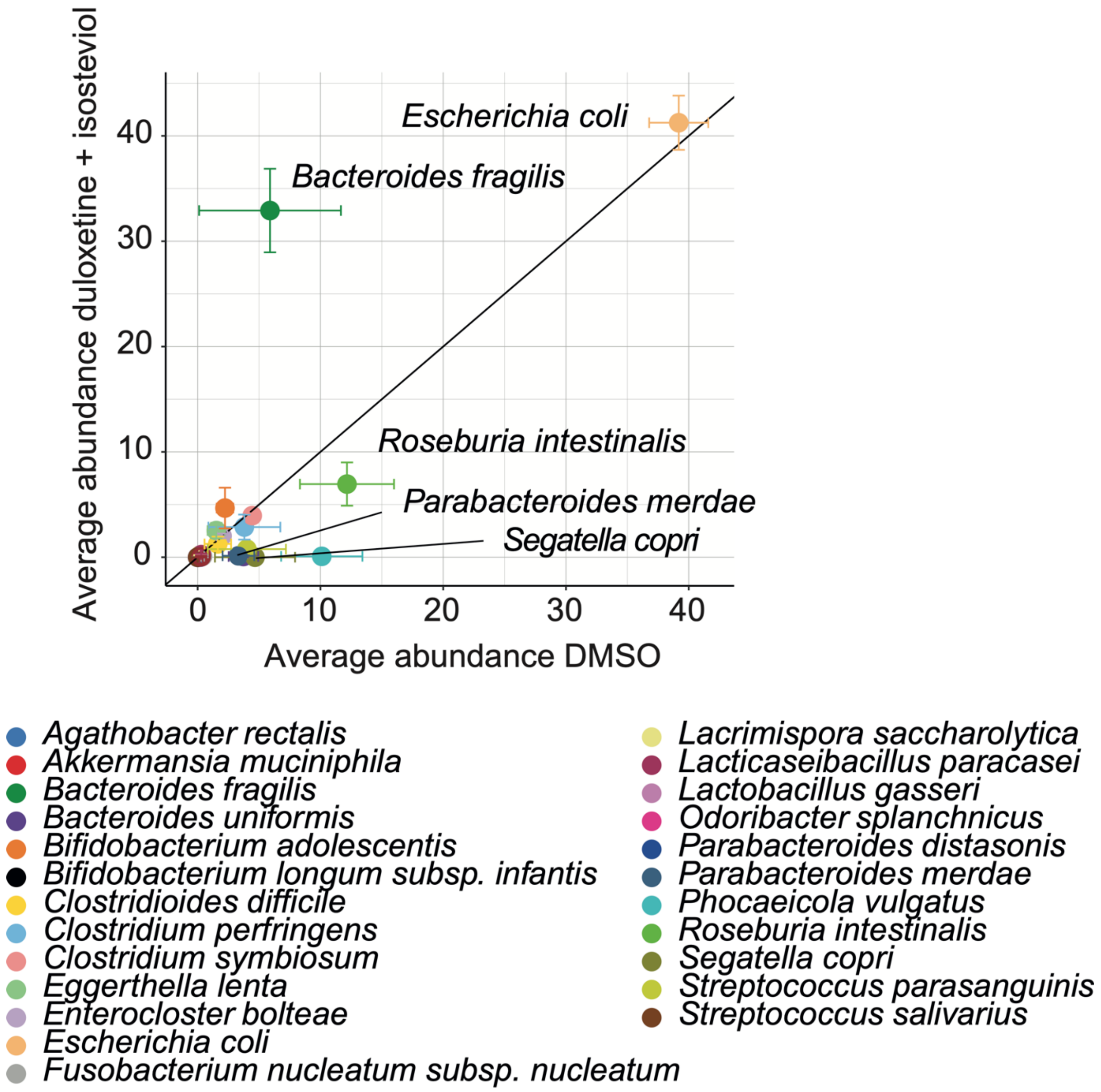

Appendix Figure S6. Relative abundance per species in DMSO and isosteviol-duloxetine in fifth (final) passage, species below diagonal are reduced abundance in isosteviol-duloxetine treated communities.

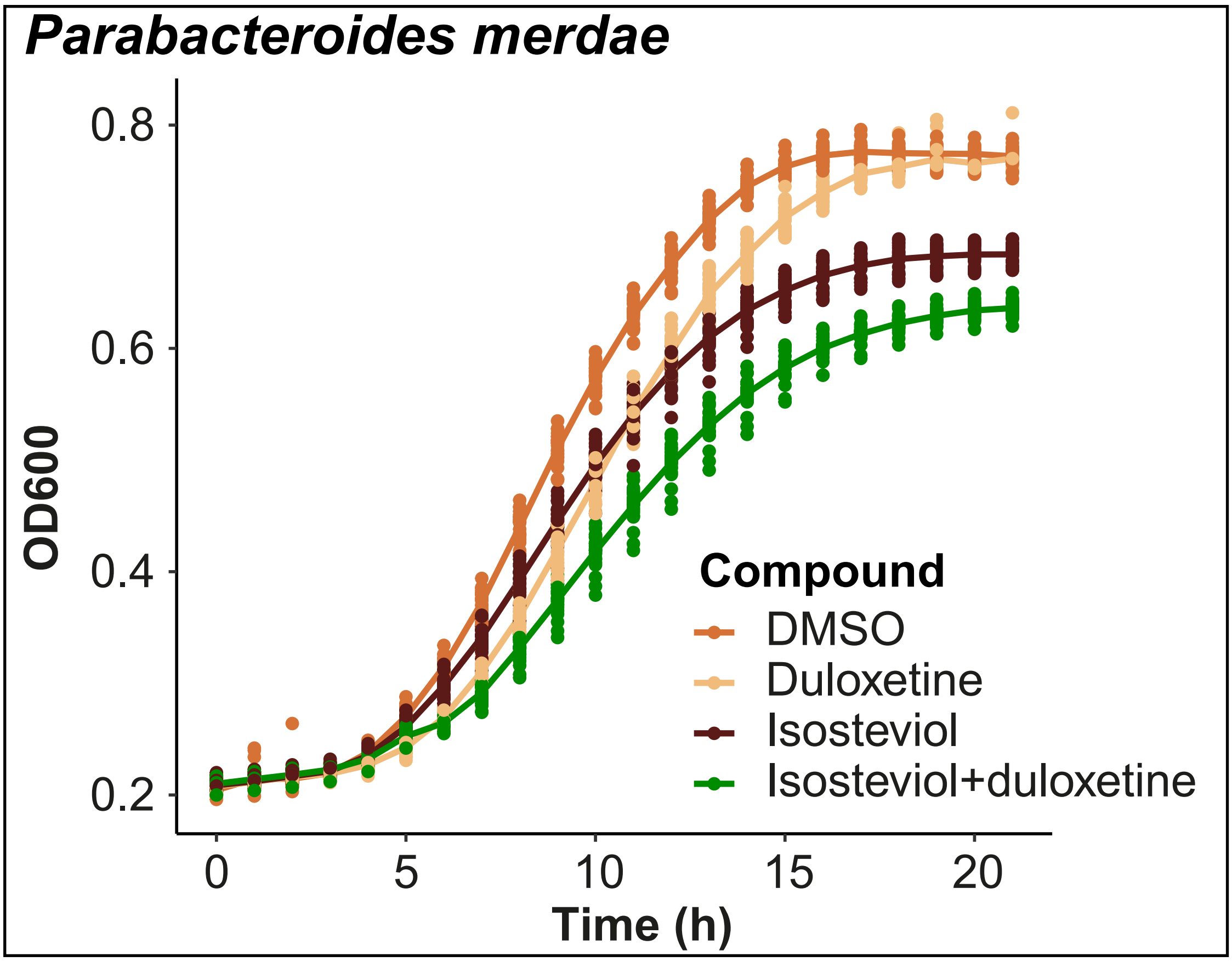

Appendix Figure S7. Growth inhibition of *Parabacteroides merdae* by duloxetine, isosteviol and a combination of both as compared to the compound solvent DMSO (1%) at a concentration of 50 µM each. N=24

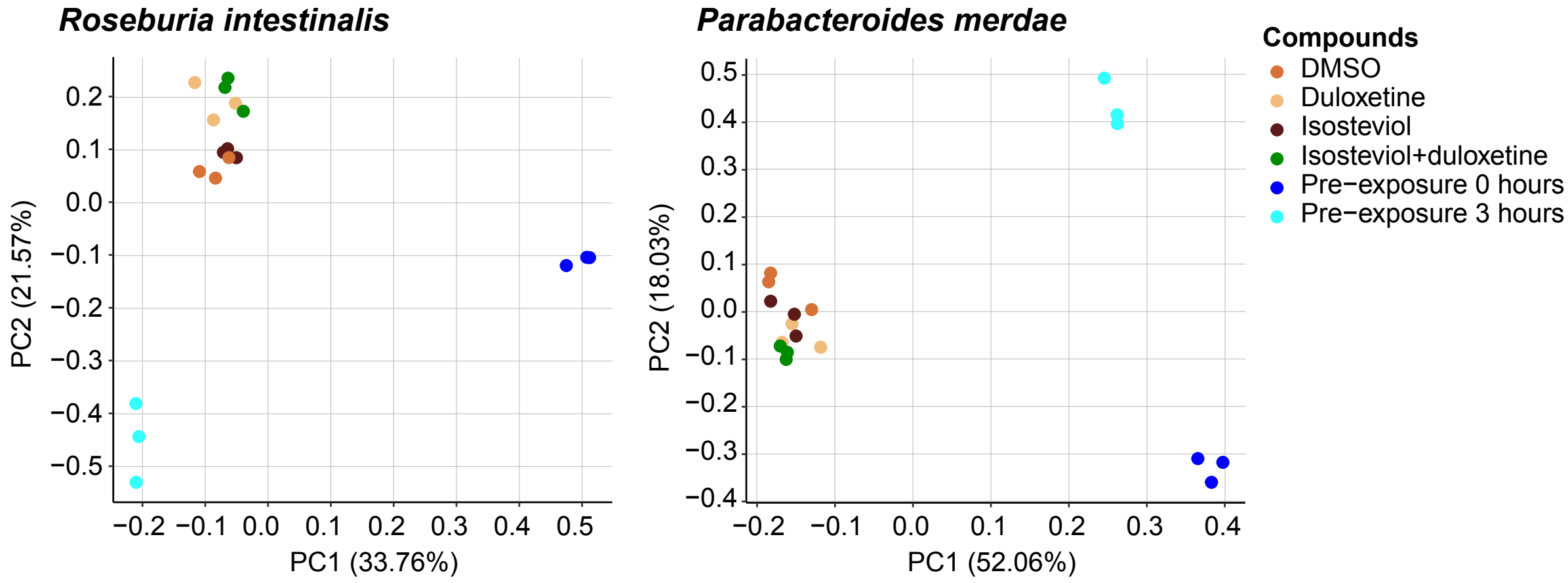

Appendix Figure S8. Differences in protein expression in *Roseburia intestinalis* and *Parabacteroides merdae* in different growth stages after culture inoculation. Pre-exposure at 0 hours and at 3 hours without drugs and compounds. Compounds (DMSO, duloxetine, isosteviol and duloxetine + isosteviol, 50 µM each) were added after collection of the 3-hour sample and the bacteria were exposed for 4 hours to each of the compounds. The compound-treated samples were harvested after a total growth time of 7 hours (end of logarithmic growth phase). N=3

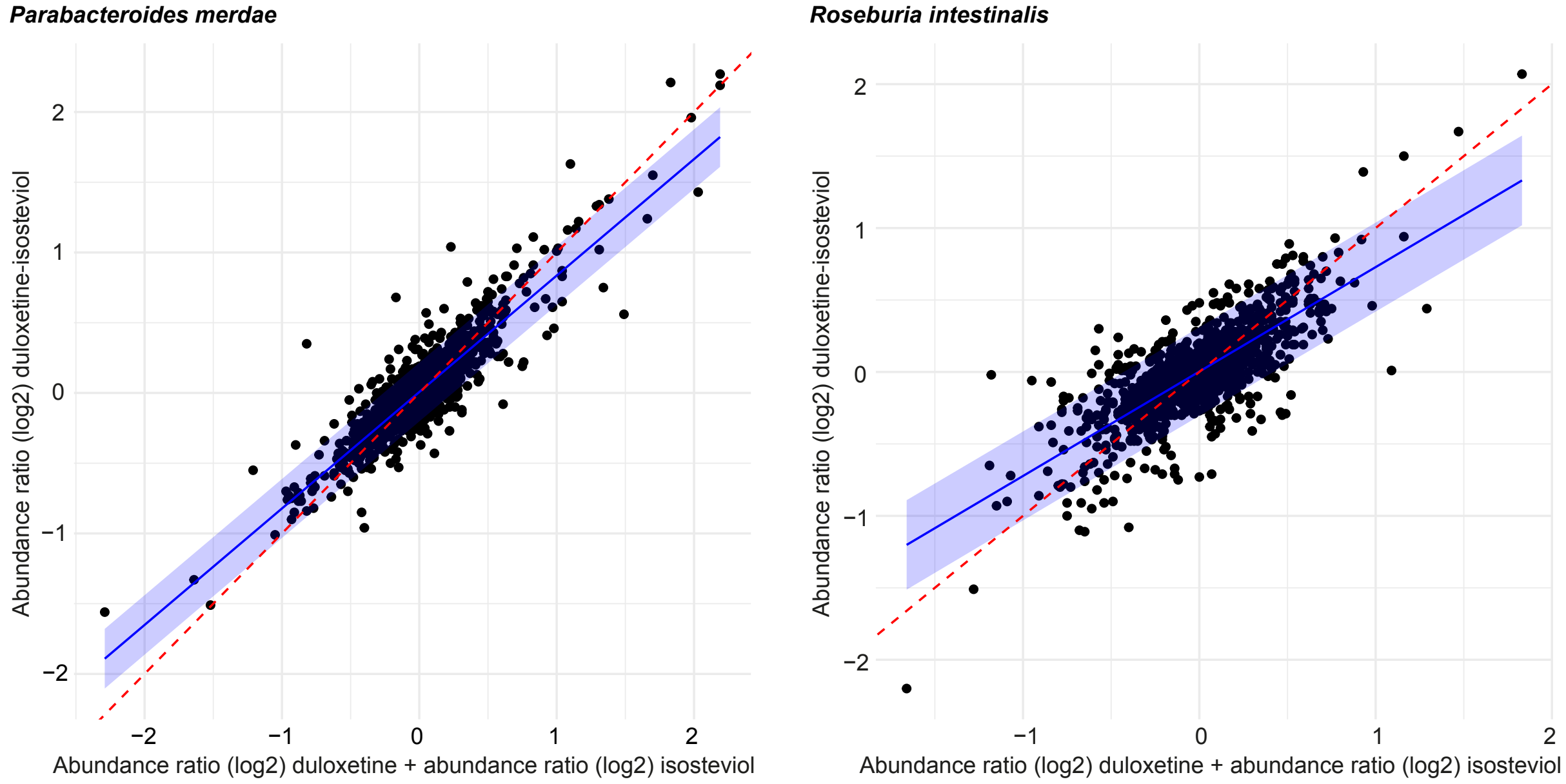

Appendix Figure S9. The effect of the combined treatment with duloxetine and isosteviol (y-axis) differs from the addition of the effect of each of the individual compounds in both, *P. merdae* and *R. intestinalis*. This difference is more pronounced in *R. intestinalis* (slope: 0.7257292, R2: 0.6025763) as compared to *P. merdae.* (slope: 0.828918, R2: 0.8049975).

a

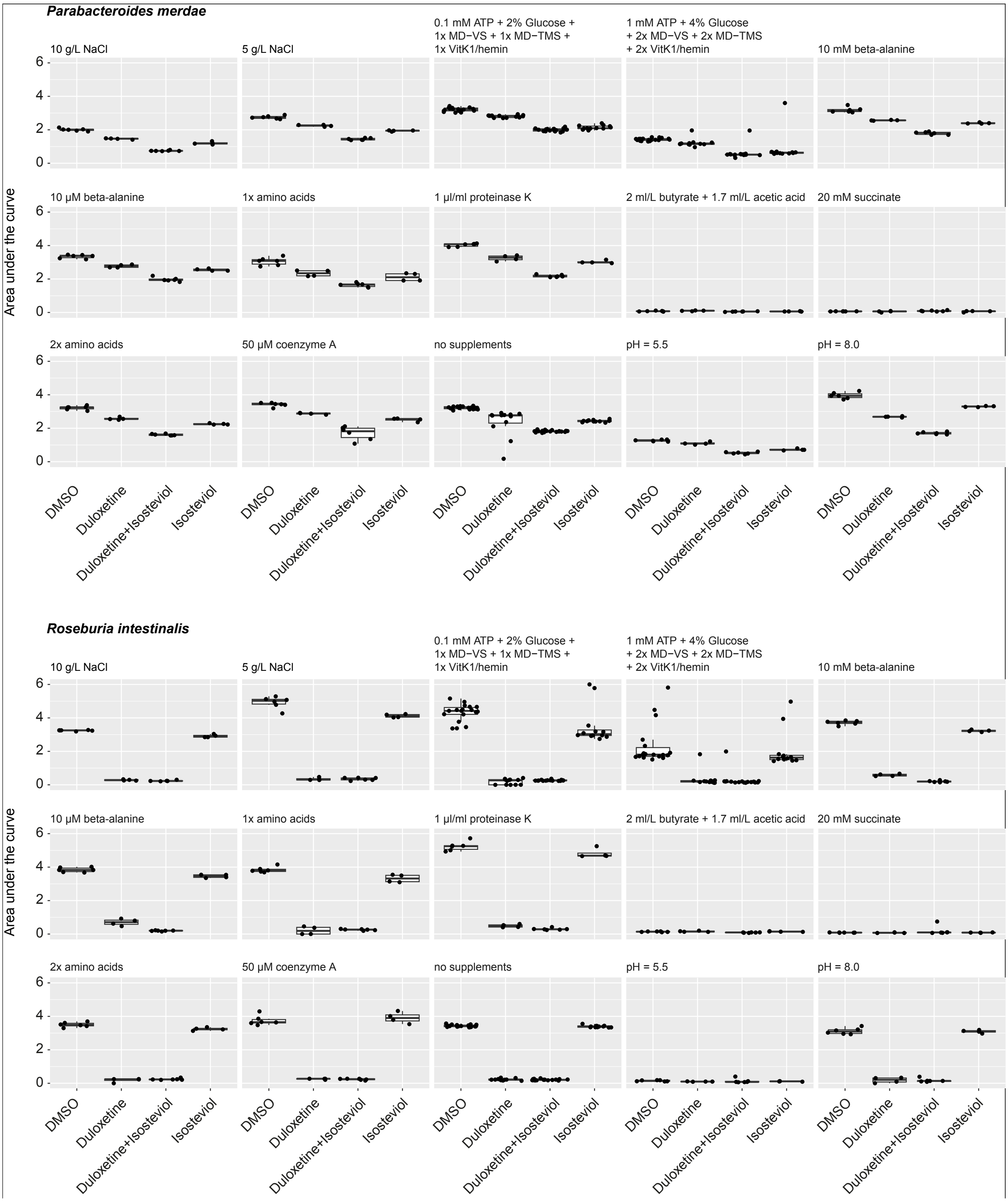

Parabacteroides merdae
10 g/L NaCl
5 g/L NaCl
0.1 mM ATP + 2% Glucose + 1x MD−VS + 1x MD−TMS + 1x VitK1/hemin
1 mM ATP + 4% Glucose + 2x MD−VS + 2x MD−TMS + 2x VitK1/hemin
10 mM beta-alanine
10 µM beta-alanine
1x amino acids
1 µl/ml proteinase K
2 ml/L butyrate + 1.7 ml/L acetic acid
20 mM succinate
2x amino acids
50 µM coenzyme A
no supplements
pH = 5.5
pH = 8.0
Area under the curve
0
2
4
6
DMSO
Duloxetine
Duloxetine+Isosteviol
Isosteviol
Roseburia intestinalis

b

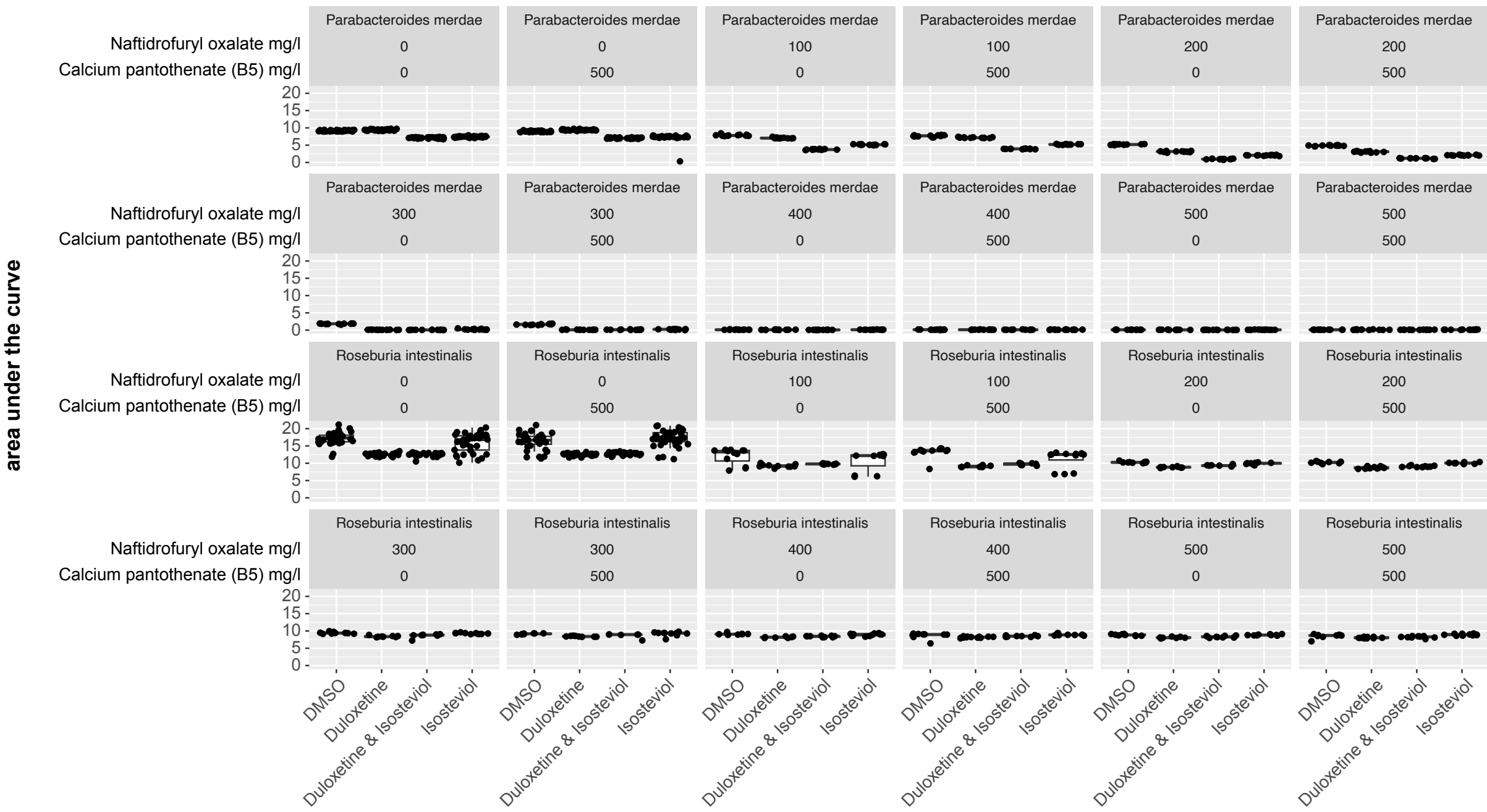

Appendix Figure S10. (a) A selection of the supplements was tested for their effect on the synergy between isosteviol and duloxetine. Although several supplements were able to improve or inhibit the growth of *R. intestinalis* and *P. merdae*, neither of them alone nor in combination was able to specifically affect the synergy. N = 6. (b) Different concentrations of naftidrofuryl (nafronyl) oxalate (an inhibitor of pantothenate synthetase, panC) and calcium pantothenate were tested in different combinations in the two bacteria exposed to isosteviol, duloxetine and the combination, N=12. Naftidrofuryl oxalate inhibited the growth, while calcium pantothenate was unable to restore it.

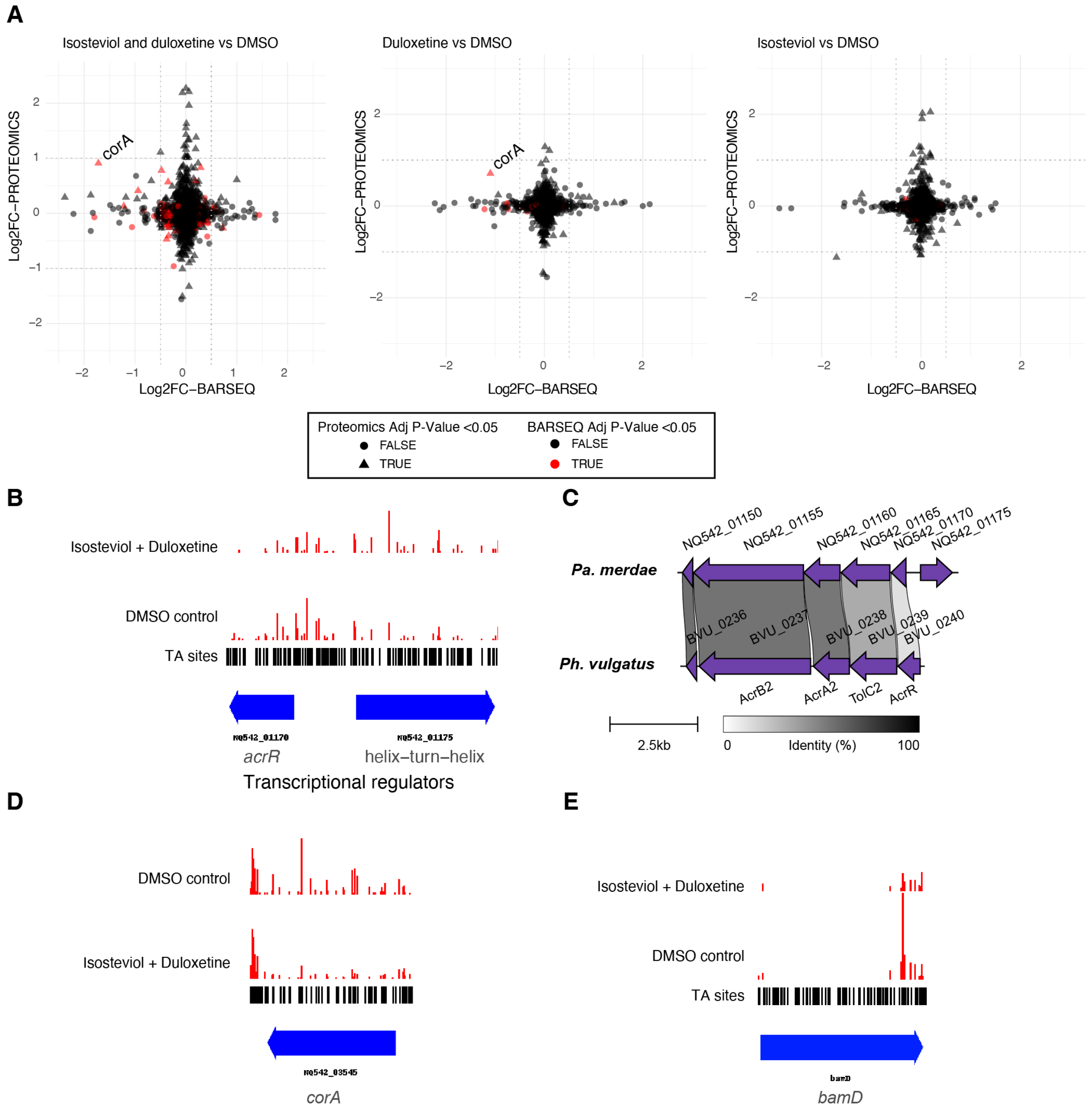

Appendix Figure S11. A. Comparison of gene fitness (BarSeq) with the expression values of the corresponding proteins (proteomics) under duloxetine, isosteviol and co-exposure compared to DMSO control. Only genes detected and matched in both datasets are shown (as per Table EV15). Each dot represents a gene, with significant hits in BarSeq indicated in red, and significant in proteomic hits as a triangle. CorA magnesium transporter stands as an upregulated gene with strong negative fitness. **B.** Representative map of insertions in *acrR* transporter gene (negative fitness), a divergent helix-turn-helix regulator gene (positive fitness) and intergenic region (negative fitness). The blue arrows represent genes, and the reads from transposon insertions are represented in red. The presence of TA sites (preferential transposon insertion site) is shown as a black track. **C.** Representative schematic of the genetic neighbourhood of the transcriptional regulator gene *acrR* located proximal to *acrB-tolC* genes, as its orthologue recently characterised as modulator of efflux transport in *B. vulgatus* (Hibberd *et al*, 2017) made with clinker (Gilchrist & Chooi, 2021). This suggests that regulation of efflux is involved in detoxification of duloxetine and combination. **D.** Representative map of insertions in *corA* **E.** Representative map of insertions at the C-terminal

domain of the outer membrane protein assembly factor BamD. Although a cut-off of insertions in central 80% of the coding gene was applied when computing gene fitness score, several *bamD* partial knock outs show a decreased fitness under duloxetine, and isosteviol + duloxetine.

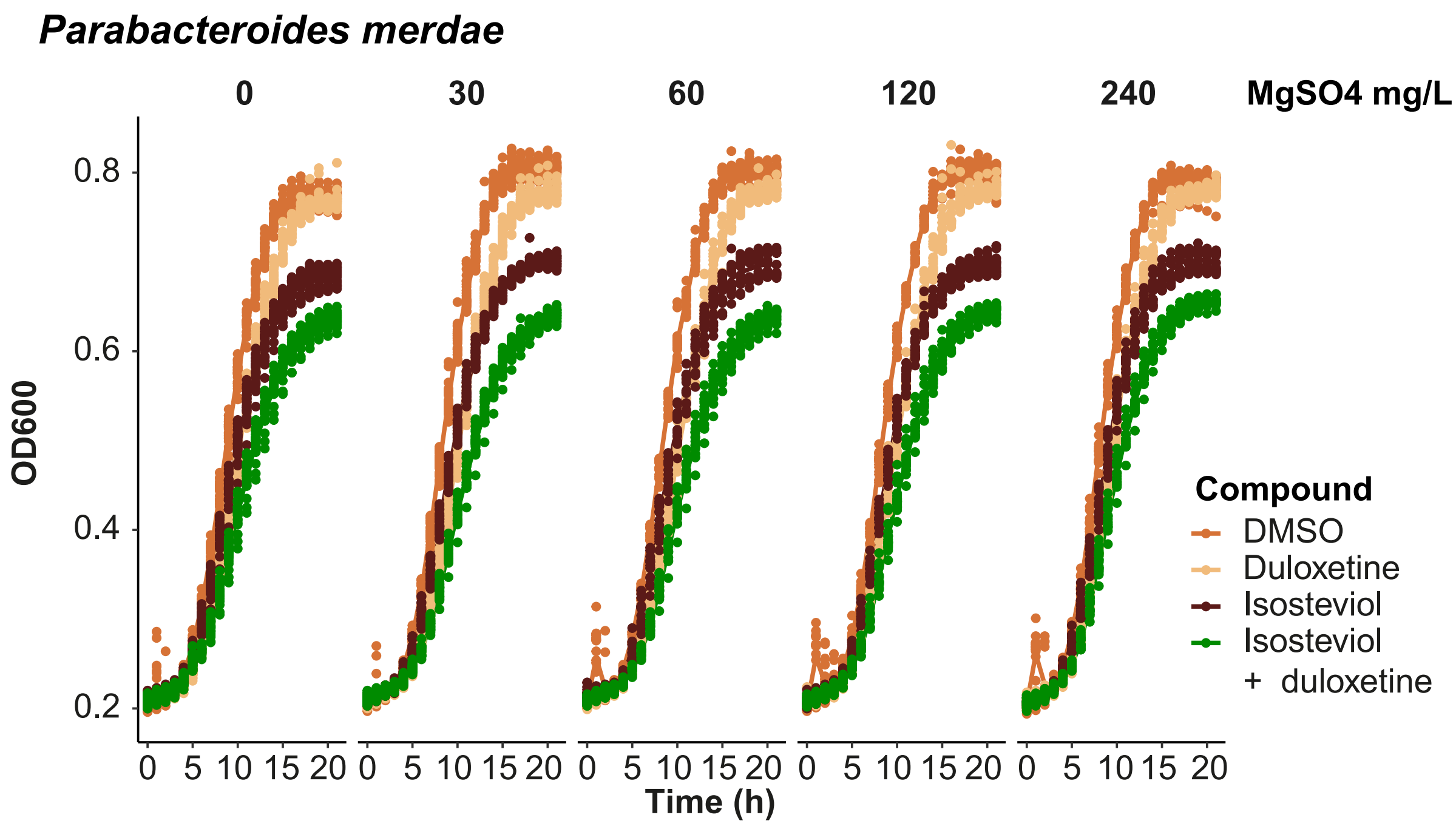

Appendix Figure S12. Supplementation of *P. merade* with magnesium sulfate (MgSO4) at different concentrations. MgSO4 did not alter growth up to a concentration of 240 mg/L in mGAM medium. N=24.

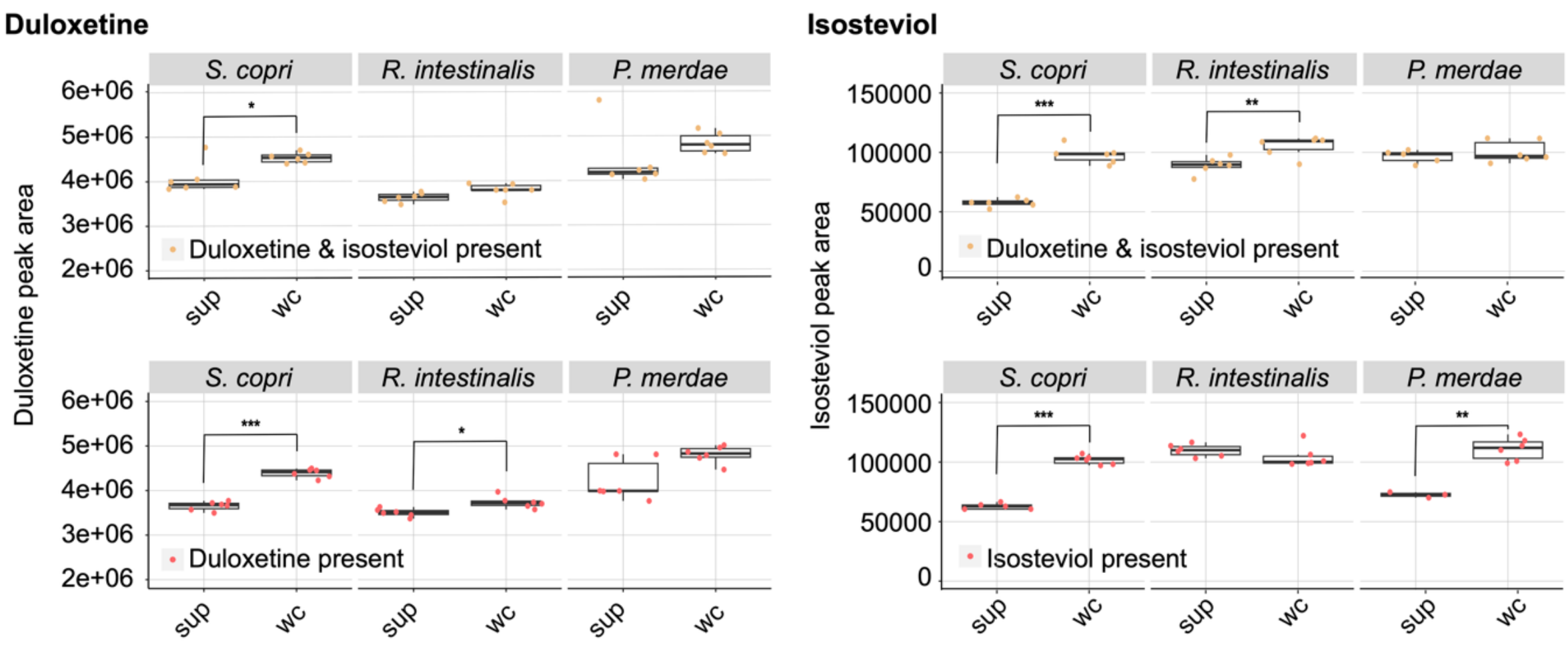

Appendix Figure S13. Bioaccumulation of duloxetine and isosteviol in *Segatella copri*, *R. intestinalis* and *P. merdae* when applied alone and in combination. *S. copri* bioaccumulates each, duloxetine and isosteviol, equally when applied alone and in combination. *R. intestinalis* bioaccumulates duloxetine only in absence of isosteviol and isosteviol only i presence of duloxetine. However, the quantitative difference is small. *P. merdae* bioaccumulates isosteviol only in absence of duloxetine. * $p<0.05$, ** $p<0.01$, *** $p<0.001$, N=6, wc=whole culture, sup=supernatant, AUC = area under the curve.

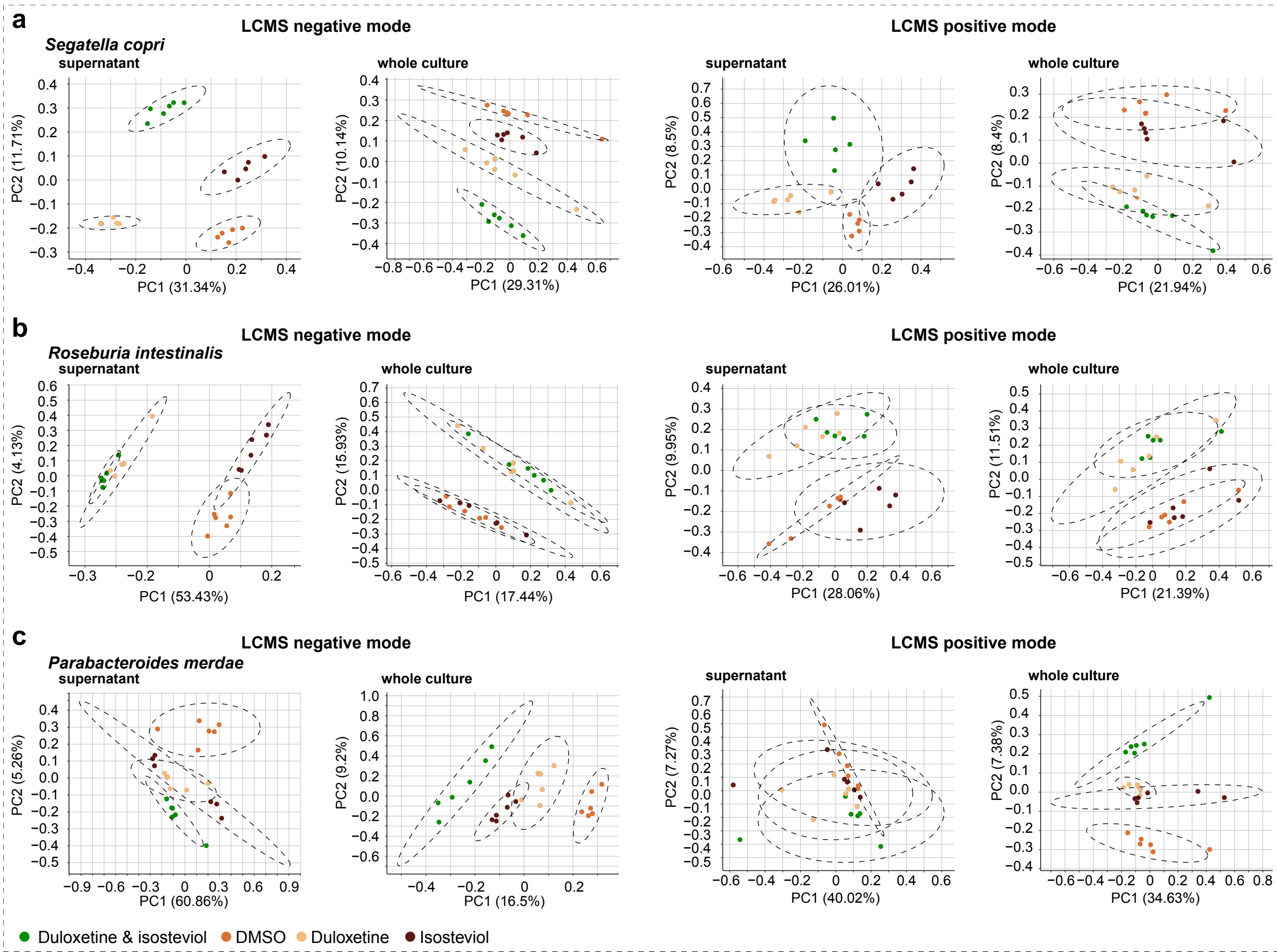

Appendix Figure S14. Altered metabolite production and secretion in *S. copri*, *R. intestinalis* and *P. merdae* upon overnight exposure to duloxetine, isosteviol and the combination of both (50 µM each in mGAM) as compared to the solvent (DMSO). N=6.

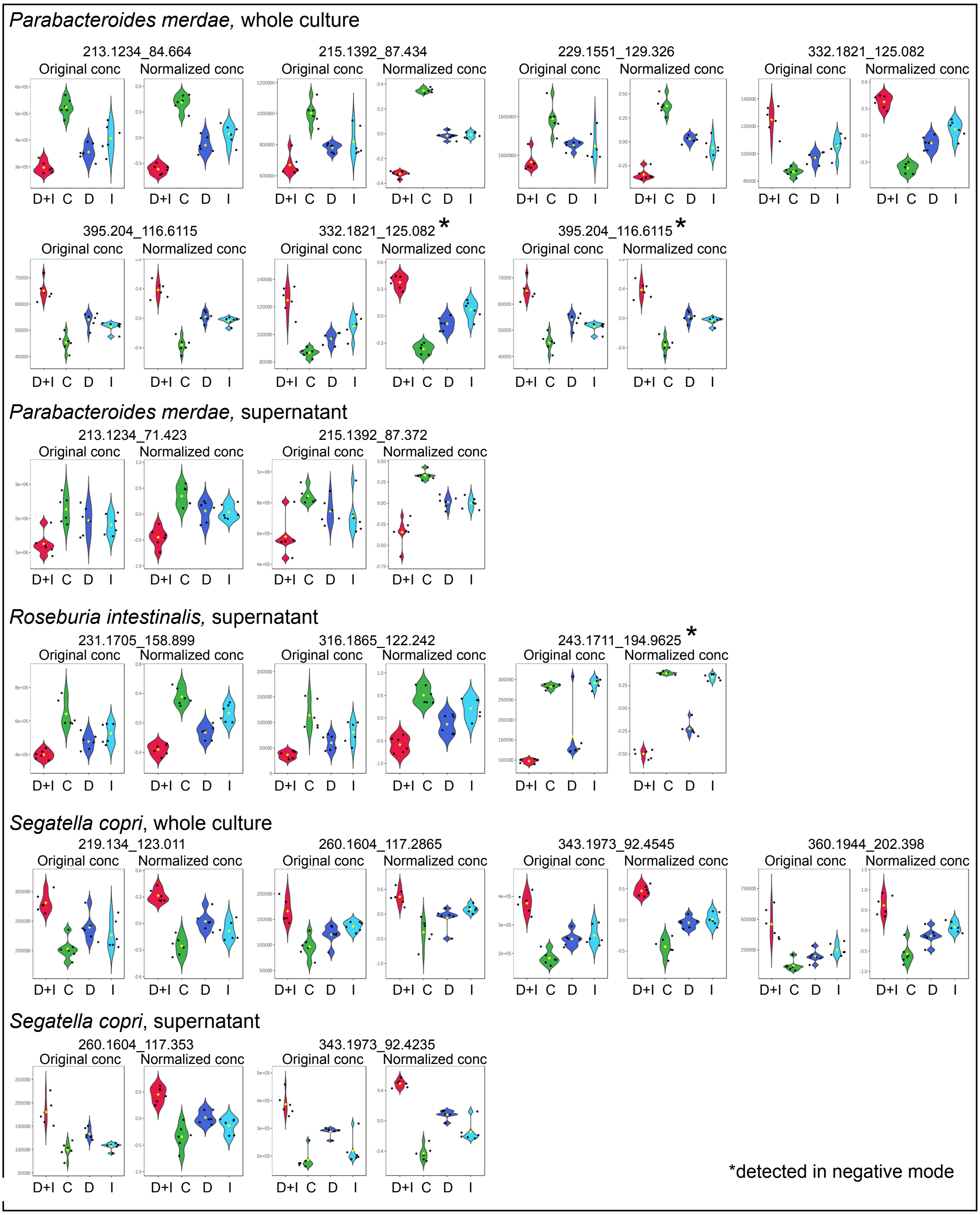

Appendix Figure S15. Metabolites affected from combined exposure to duloxetine and isosteviol of *R. intestinalis*, *P merdae* and *S. copri*. D+I = Duloxetine + isosteviol, C = control, D = duloxetine, I = isosteviol, N=6.

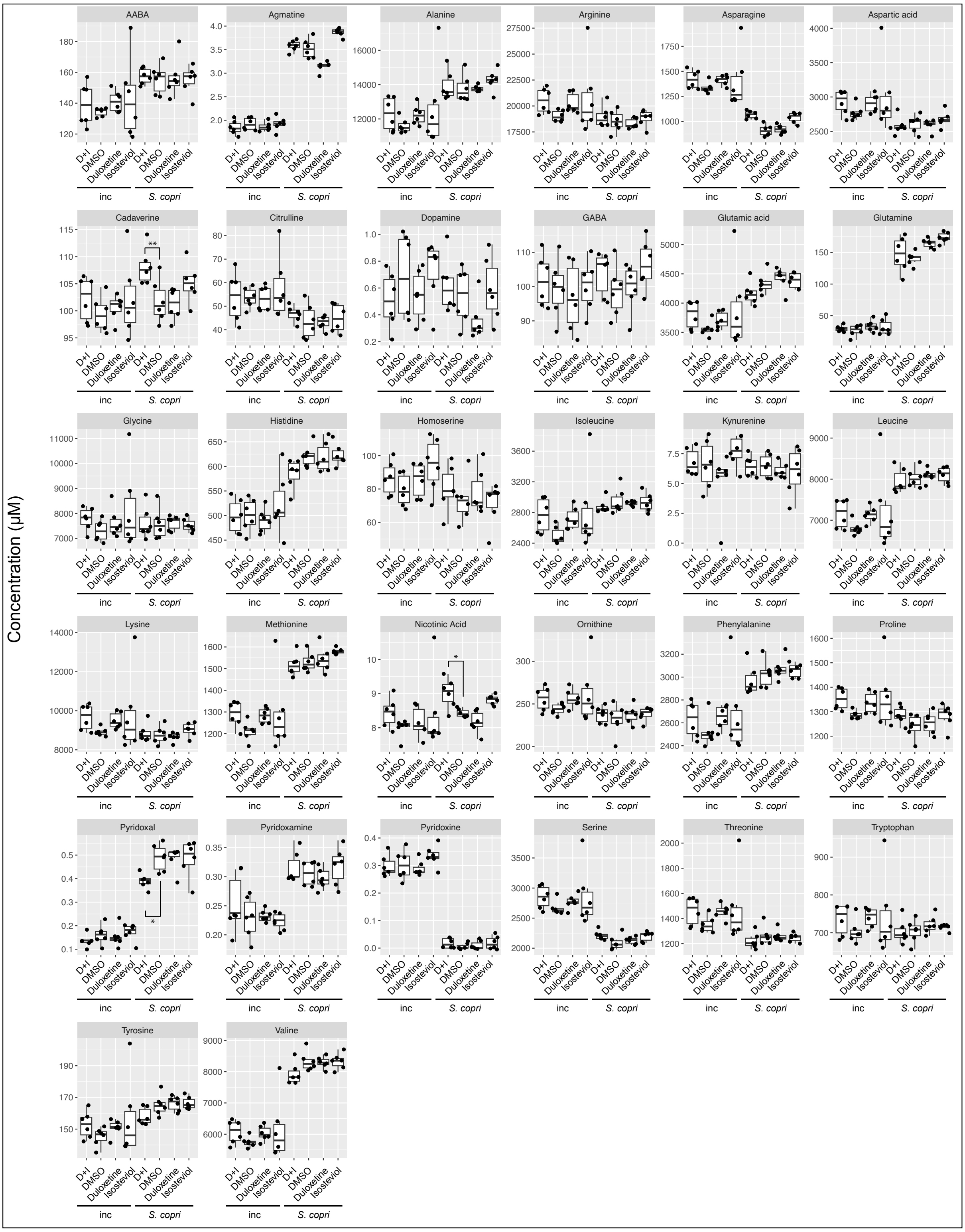

Appendix Figure S16. Changes in the concentrations of 32 amino acids and derivates in *Segatella copri* upon overnight exposure to duloxetine, isosteviol and the combination of both (50 µM each). D+I = Duloxetine + isosteviol, inc = incubation control (mGAM medium plus compounds, without bacteria). N=6, * = $p<0.05$.

Segatella copri

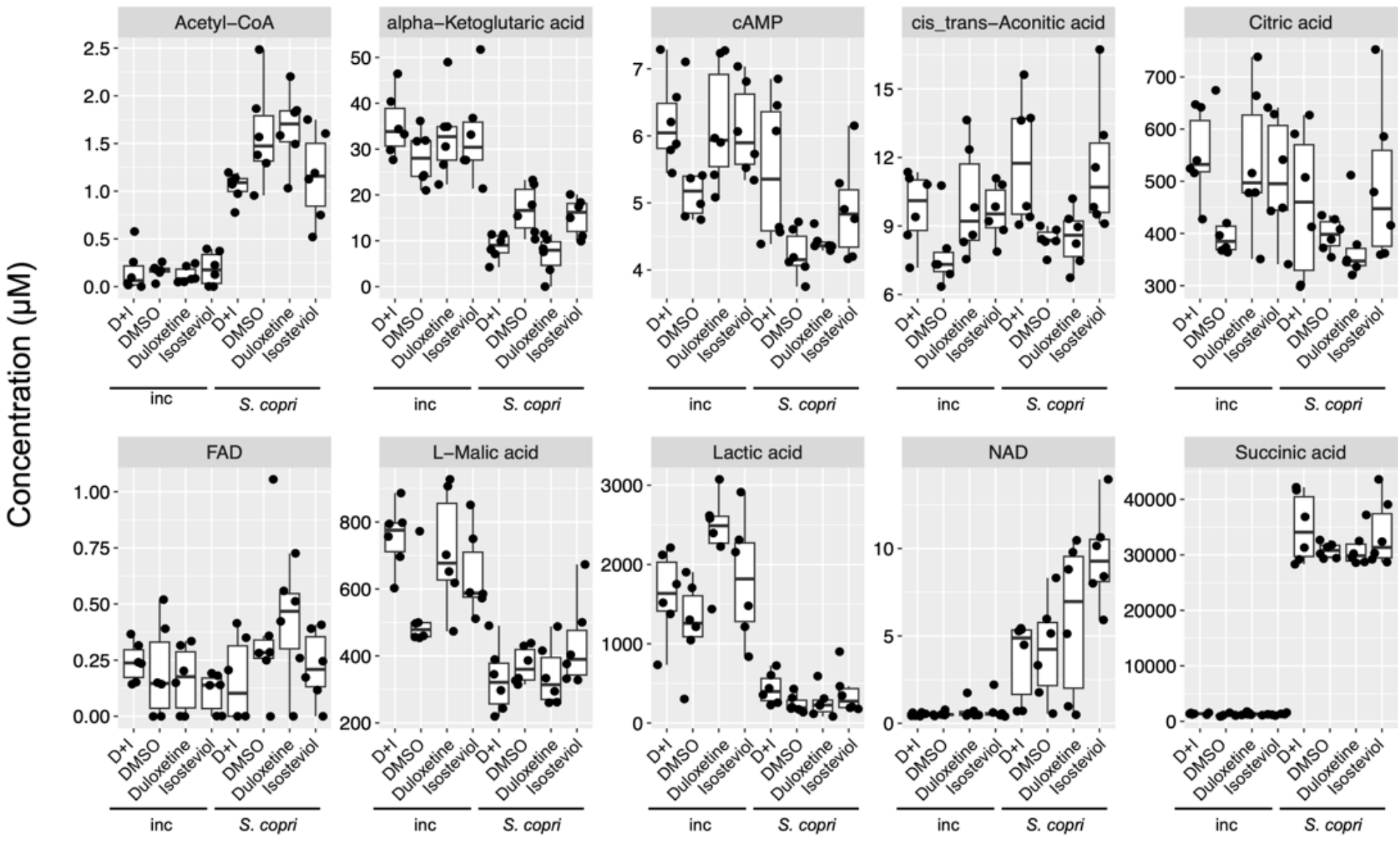

Roseburia intestinalis

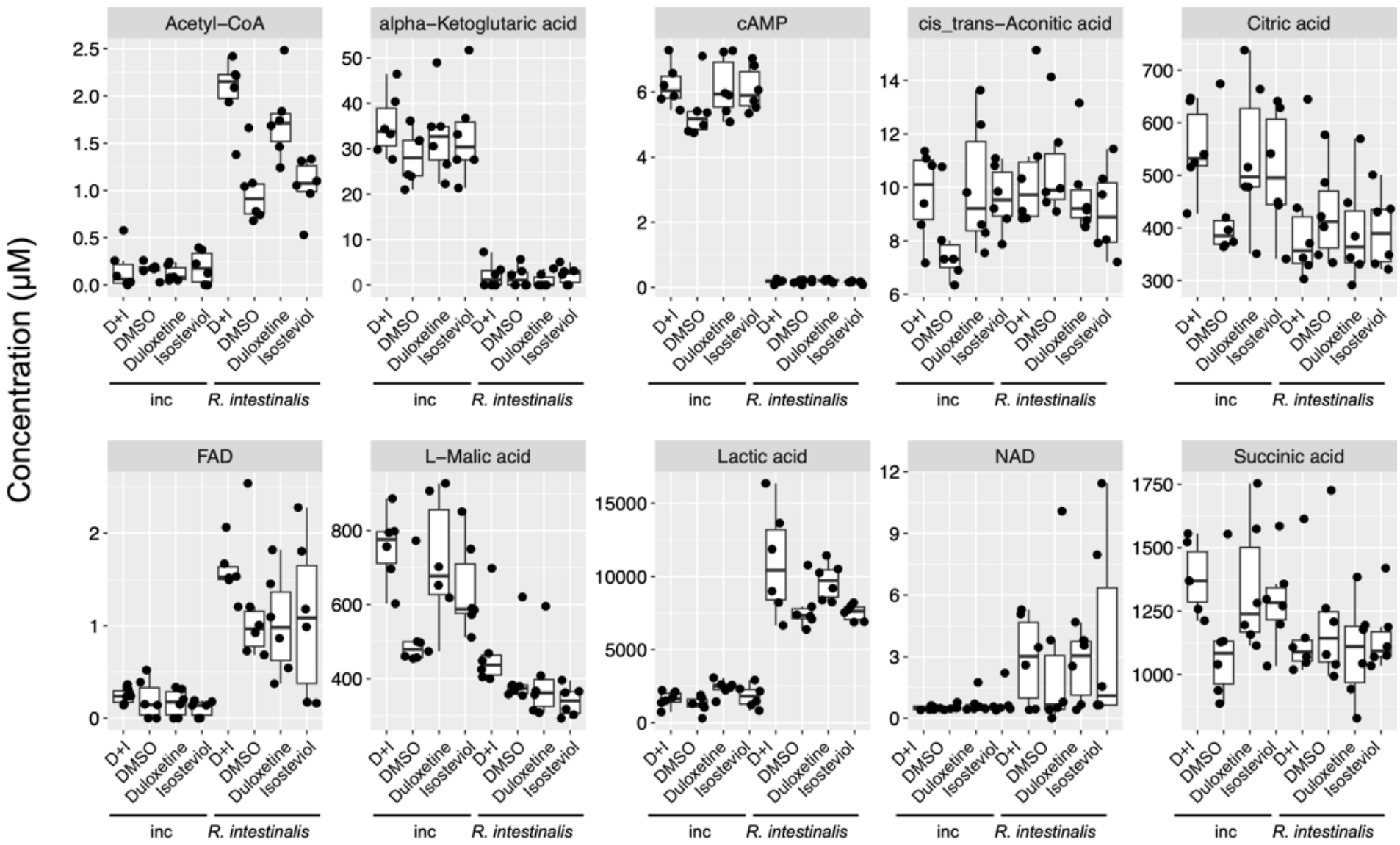

Parabacteroides merdae

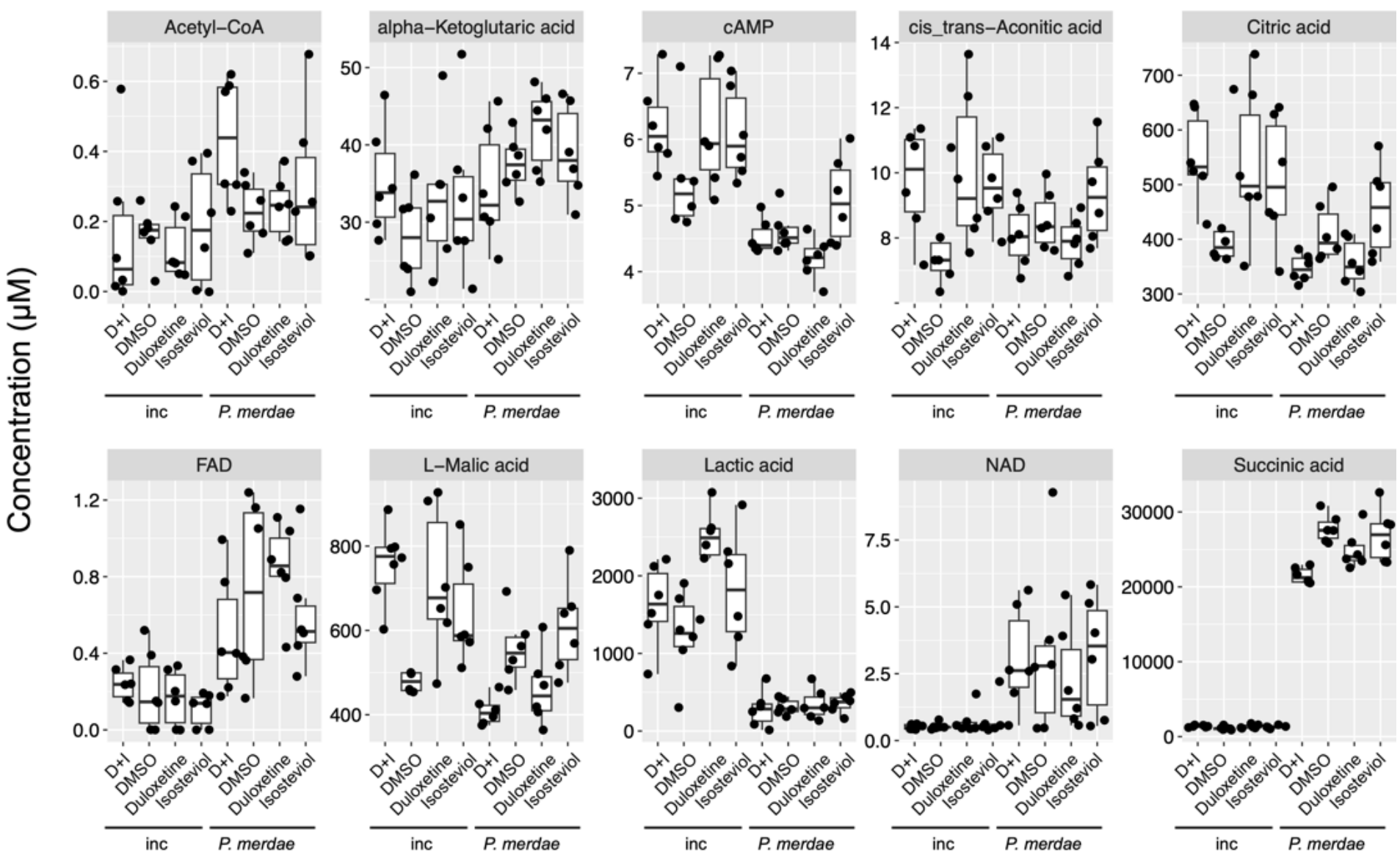

Appendix Figure S17. Changes in the concentrations of organic acids in *Segatella copri*, *Roseburia intestinalis* and *Parabacteroides merdae* upon overnight exposure to duloxetine, isosteviol and the combination of both (50 µM each). D+I = Duloxetine + isosteviol, inc = incubation control (mGAM medium plus compounds, without bacteria). N=6, * = $p<0.05$, ** = $p<0.01$, *** = $p<0.001$, **** = $p<0.0001$.

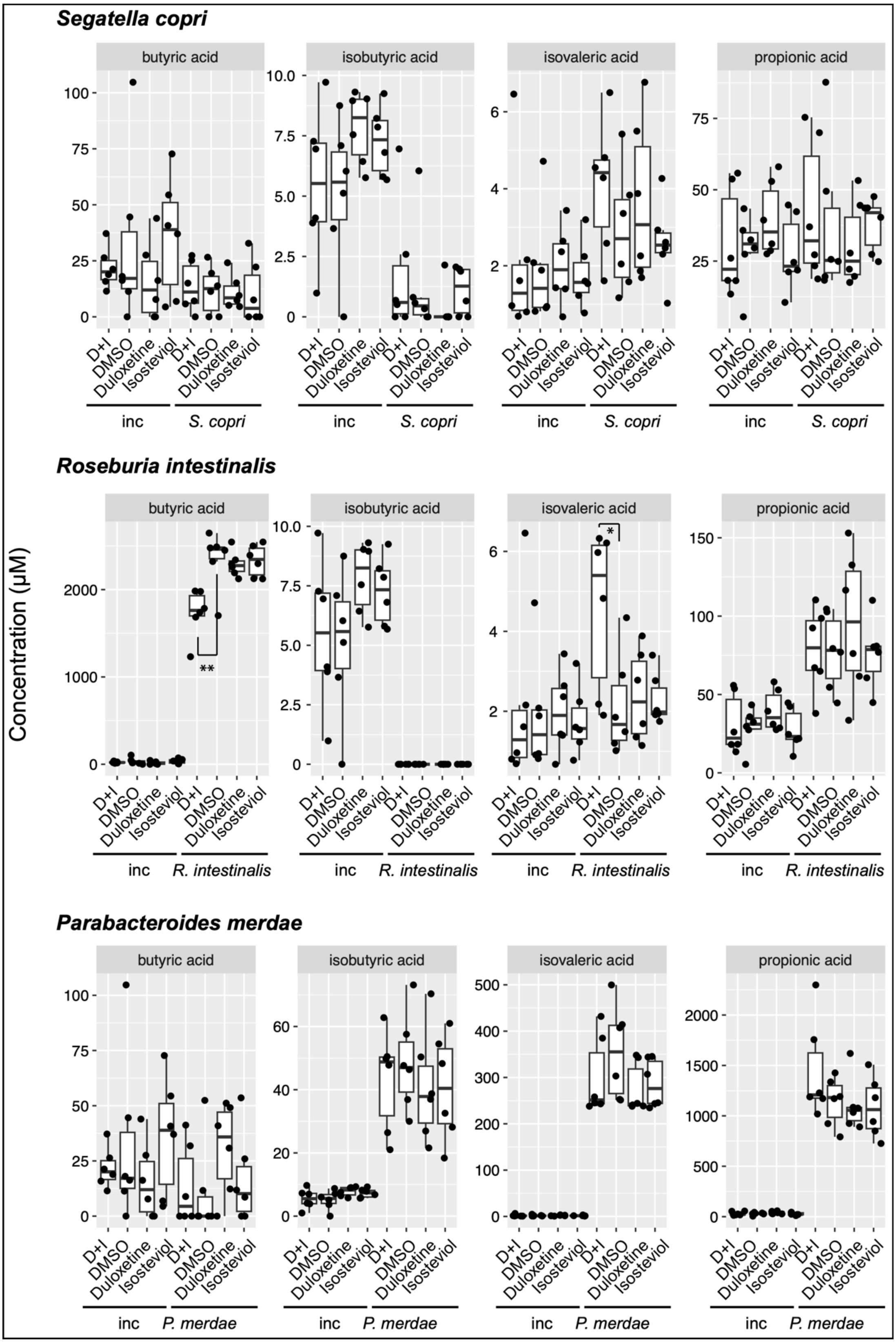

Appendix Figure S18. Changes in the concentrations of short chain fatty acids in *Segatella copri*, *Roseburia intestinalis* and *Parabacteroides merdae* upon overnight exposure to duloxetine, isosteviol and the combination of both (50 µM each). D+I = Duloxetine + isosteviol, inc = incubation control (mGAM medium plus compounds, without bacteria). N=6, * = p<0.05, ** = p<0.01, *** = p<0.001, **** = p<0.0001.

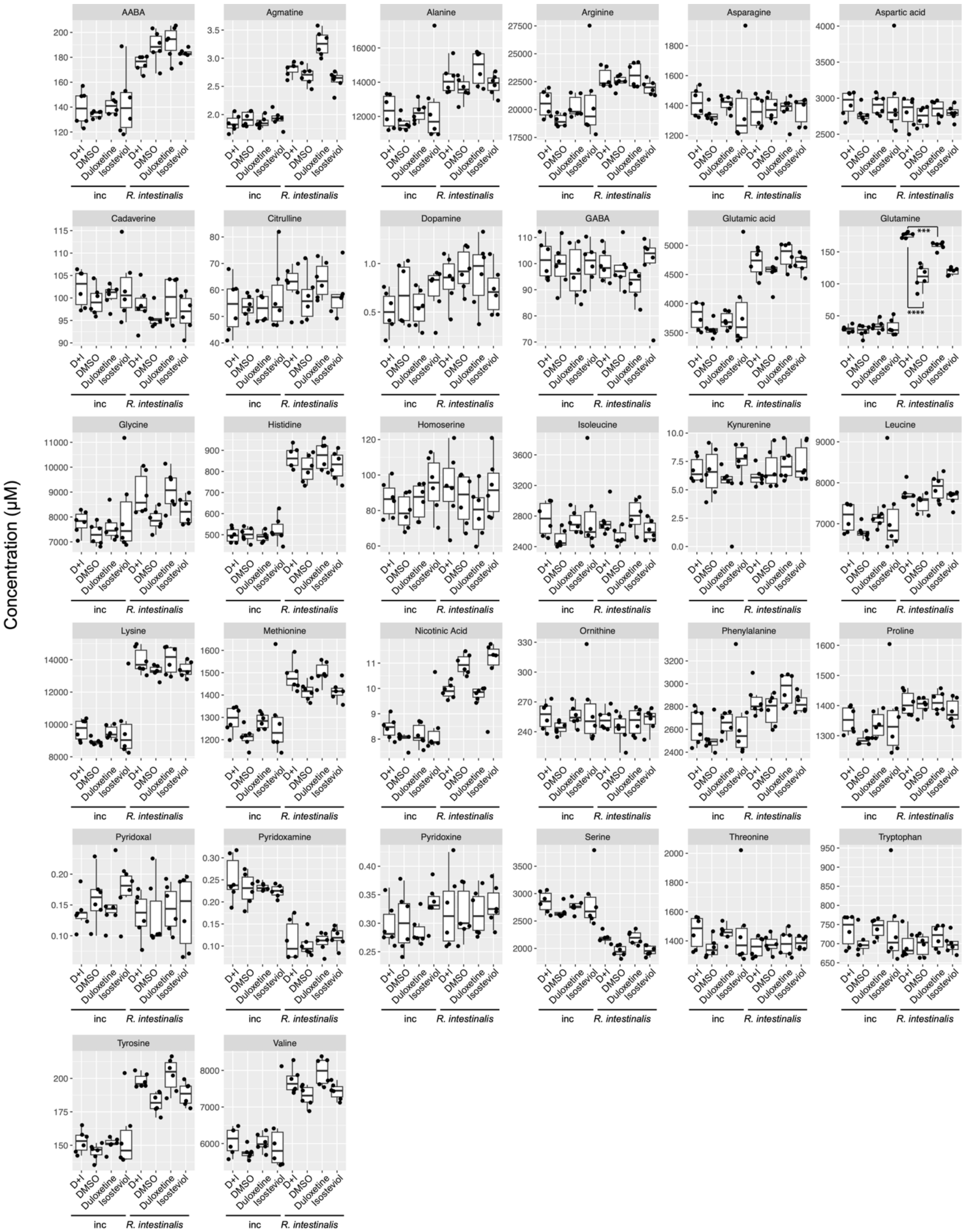

Appendix Figure S19. Changes in the concentrations of 32 amino acids and derivates in *Roseburia intestinalis* upon overnight exposure to duloxetine, isosteviol and the combination of both (50 µM each). D+I = Duloxetine + isosteviol, inc = incubation control (mGAM medium plus compounds, without bacteria). N=6, * = $p<0.05$, ** = $p<0.01$, *** = $p<0.001$, **** = $p<0.0001$.

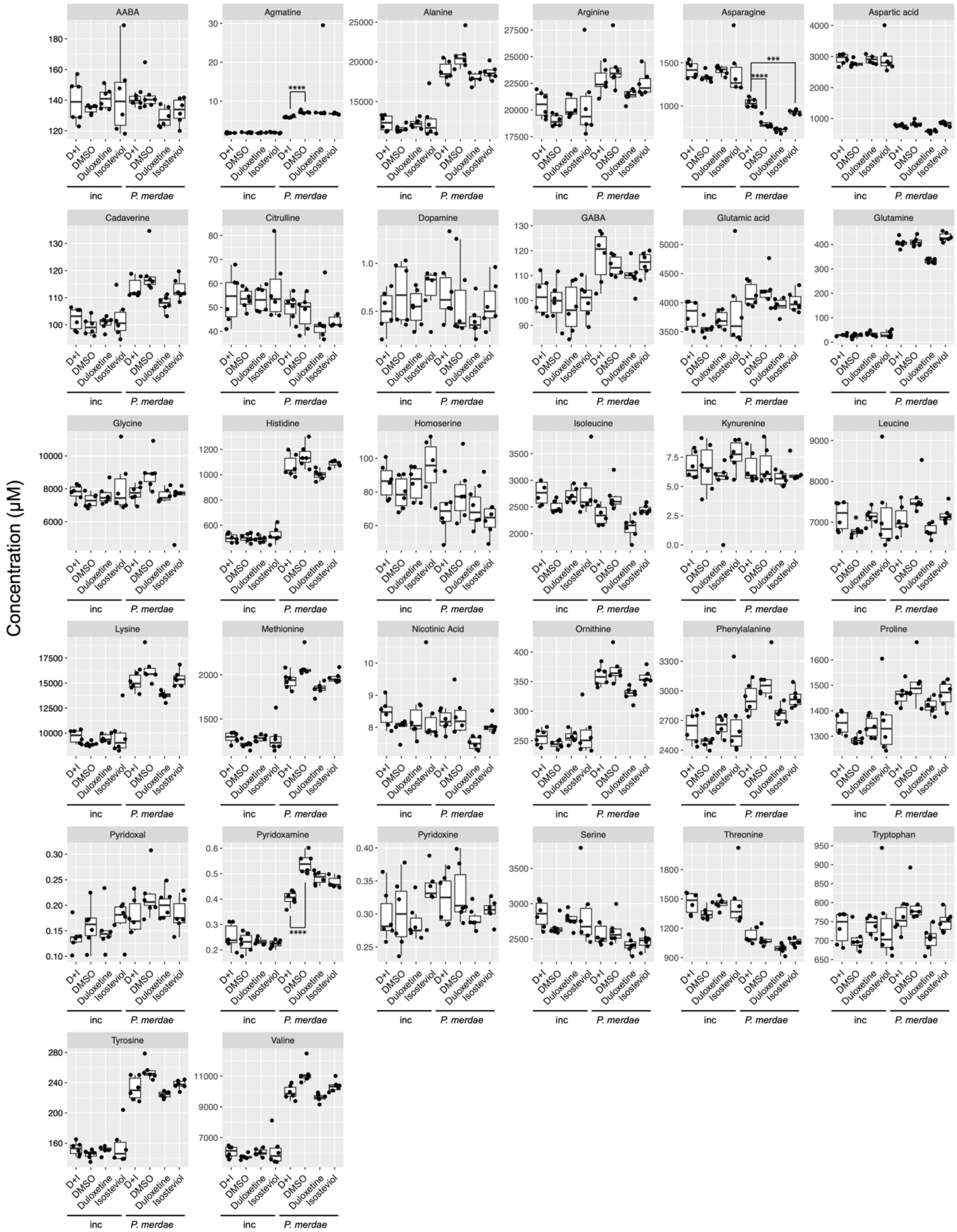

Appendix Figure S20. Changes in the concentrations of 32 amino acids and derivates in *Parabacteroides merdae* upon overnight exposure to duloxetine, isosteviol and the combination of both (50 µM each). D+I = Duloxetine + isosteviol, inc = incubation control (mGAM medium plus compounds, without bacteria). N=6, * = p<0.05, ** = p<0.01, *** = p<0.001, **** = p<0.0001.

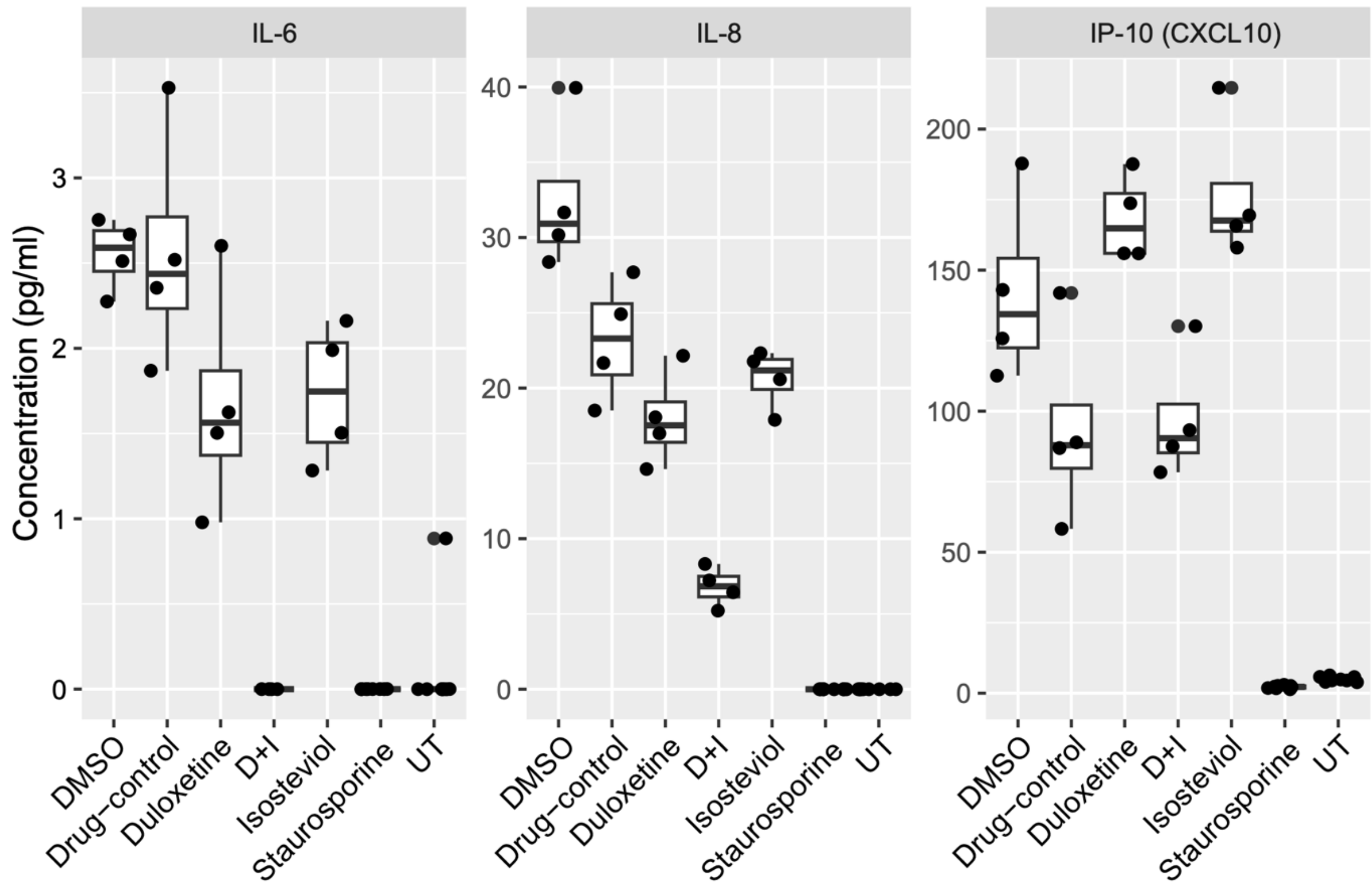

Appendix Figure S21. Secretion of the cytokines interleukin-6 (IL-6), interleukin-8 (IL-8) and the interferon-γ inducible protein 10 kDa (CXCL10) by Caco-2 cells as a response to contact with spent medium. IL-6 and IL-8 secretion is significantly reduced for the supernatant of duloxetine and isosteviol co-treated communities, as compared to the DMSO and drug control. The 25- bacteria community was treated with duloxetine, isosteviol, and duloxetine + isosteviol (D+I). Controls are DMSO (solvent, 0.2%) treated community and the supernatant of the solvent-treated community with 50 μM duloxetine and isosteviol, added after harvesting (drug control). Staurosporine and DMEM alone (UT) does not induce any of the cytokines. N>6.

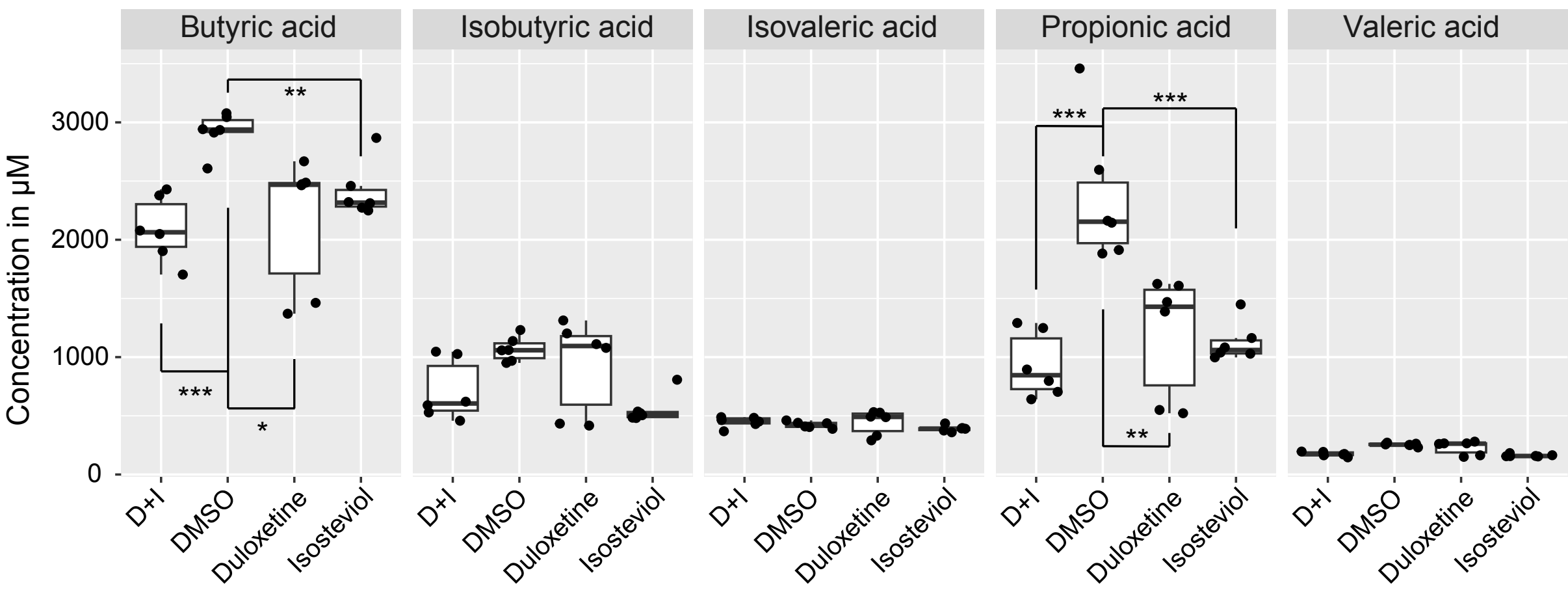

Appendix Figure S22. Concentration of the five short chain fatty acids in the supernatant of the 25 bacteria community. The supernatant contains ~3 mM butyric acid and 2 mM propionic acid. Both SCFAs are significantly decreased in duloxetine, isosteviol and duloxetine plus isosteviol (D+I) treated community supernatants. N=6, * = $p<0.05$, ** = $p<0.01$, *** = $p<0.001$.

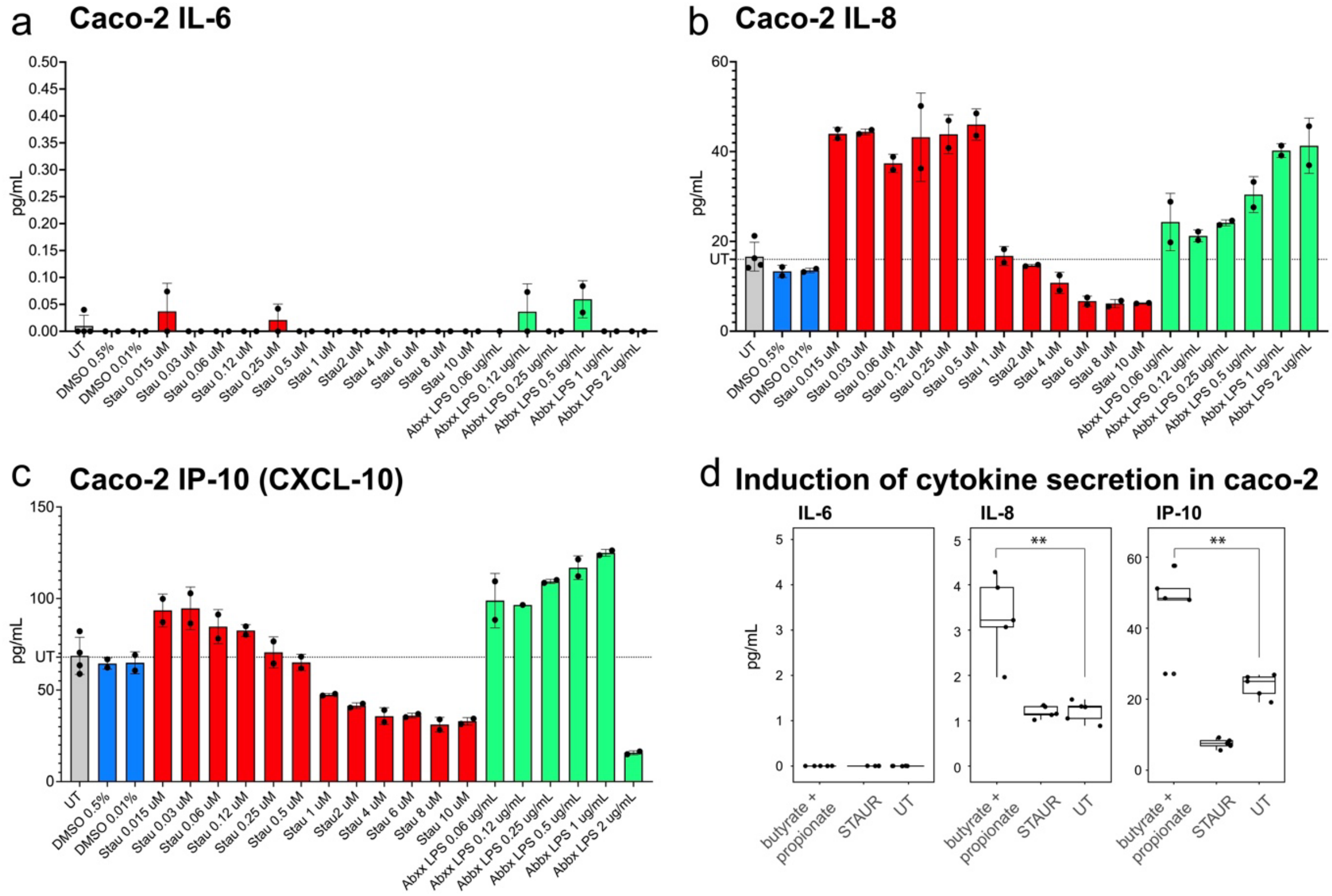

Appendix Figure S23. (a-c) Secretion of IL-6, IL-8 and IP-10 after exposure to different concentrations of staurosporine and LPS in Caco-2 cells. Secretion of each cytokine is below that of untreated cells (UT) for staurosporine (stau) concentrations above 1 µM. A concentration of 5 µM or more is required for apoptosis induction. (d) Induction of cytokine secretion in caco-2 cells after treatment with 3 mM butyrate + 2 mM propionic acid, compared to 5 µM staurosporine (STAUR) and untreated cells. ** $p<0.01$, N=5

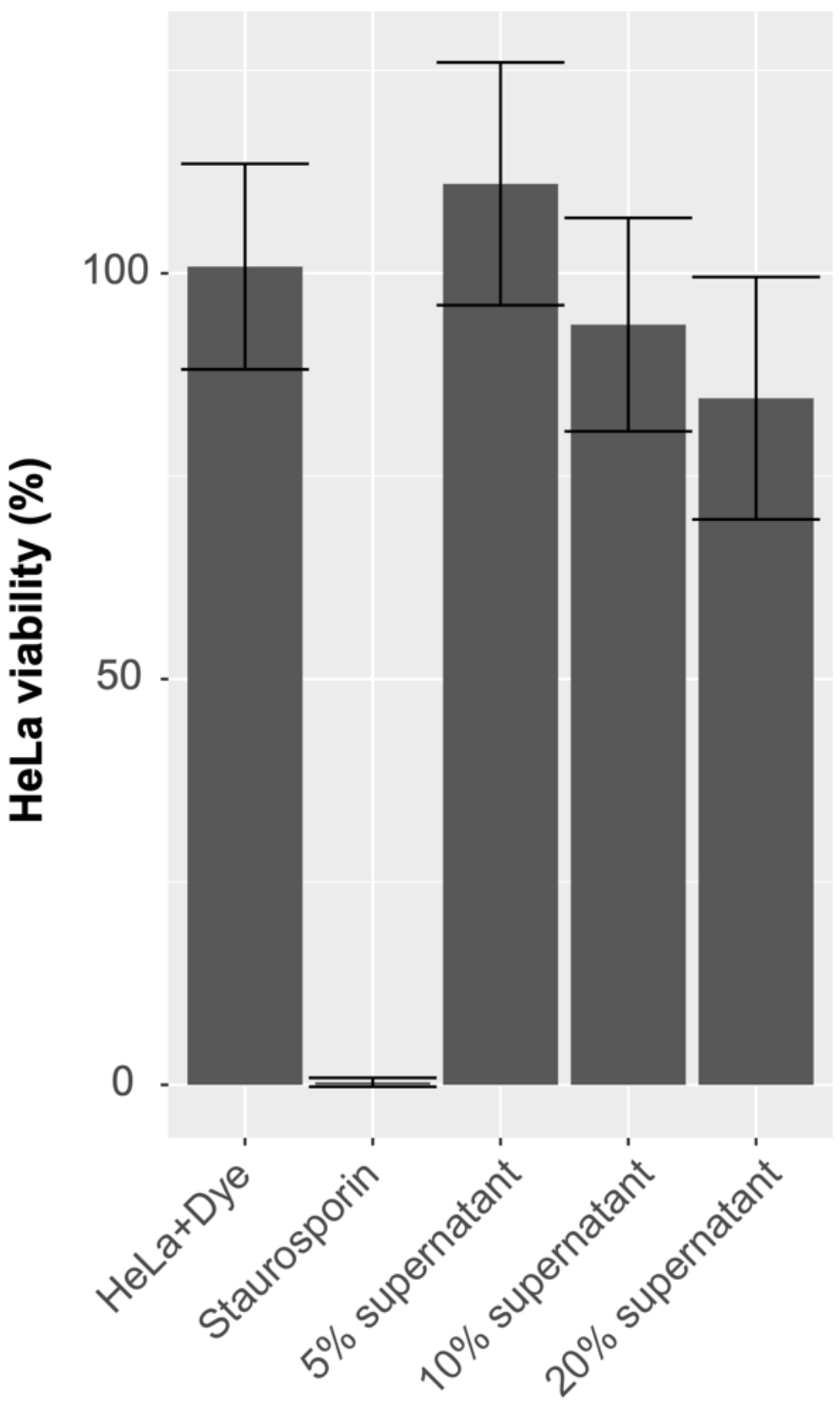

Appendix Figure S24. Viability test for HeLa cells exposed to 5, 10 and 20 % spent media from the 25 species community, done in triplicates.

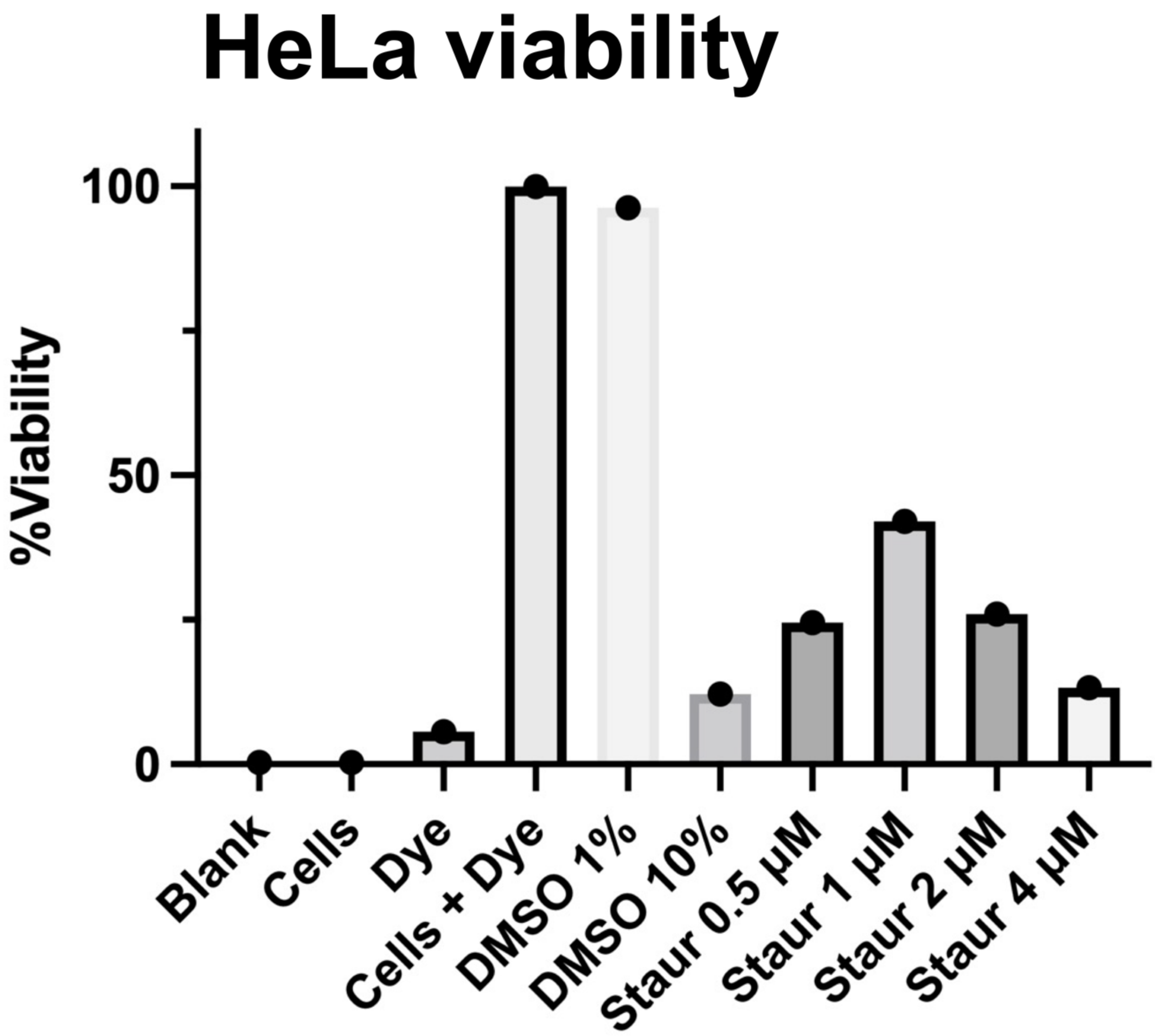

Appendix Figure S25. HeLa cell viability after treatment with different concentrations of staurosporine for apoptosis induction.

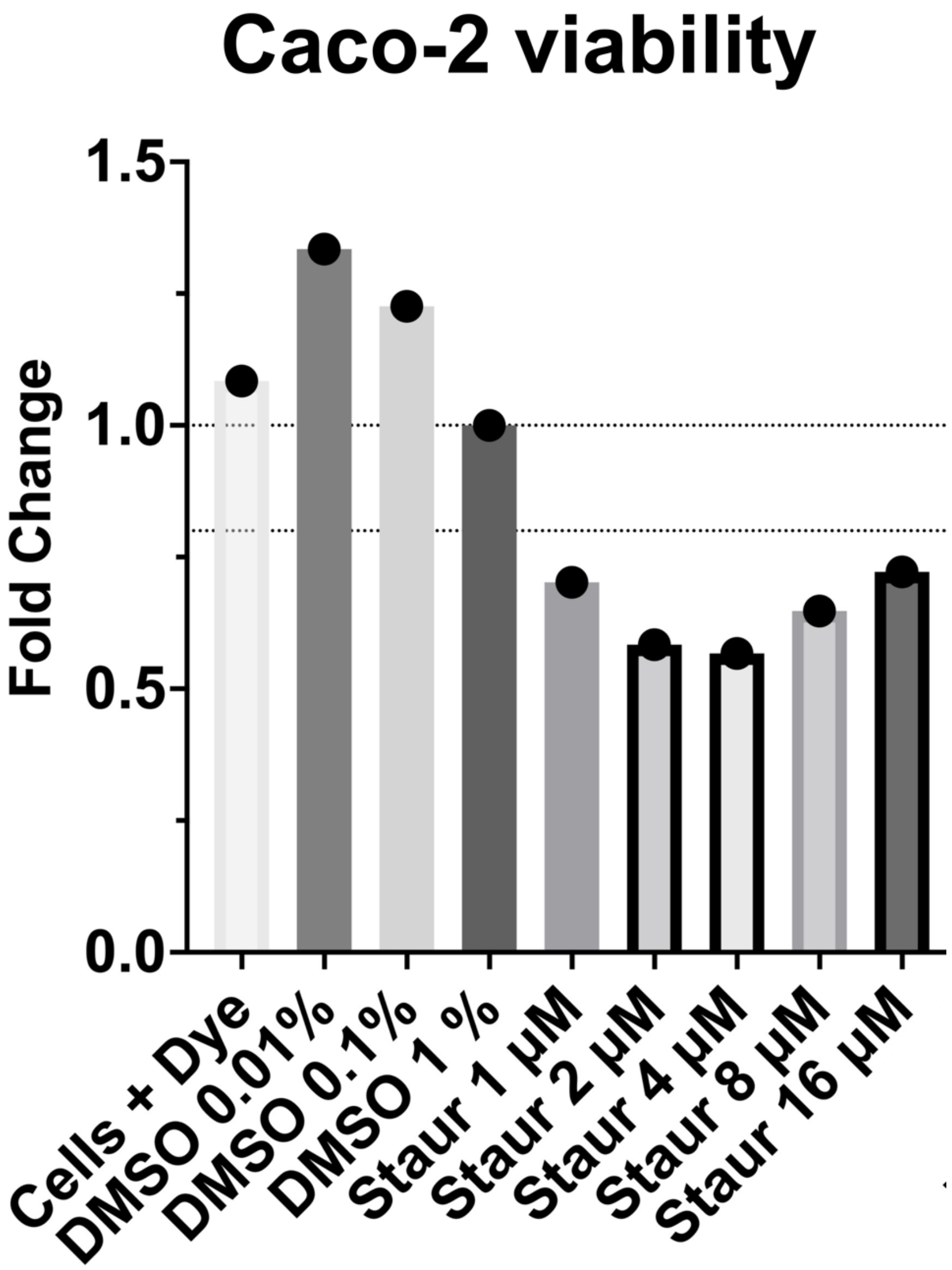

Appendix Figure S26. Caco-2 viability after treatment with different concentrations of staurosporine for apoptosis induction.

References

BLISS CI (1939) The Toxicity of Poisons Applied Jointly. *Annals of Applied Biology* 26: 585–615

Demidenko E & Miller TW (2019) Statistical determination of synergy based on Bliss definition of drugs independence. *PLoS One* 14: 1–22

Gilchrist CLM & Chooi YH (2021) Clinker & clustermap.js: Automatic generation of gene cluster comparison figures. *Bioinformatics* 37: 2473–2475

Hibberd MC, Wu M, Rodionov DA, Li X, Cheng J, Griffin NW, Barratt MJ, Giannone RJ, Hettich RL, Osterman AL, *et al* (2017) The effects of micronutrient deficiencies on bacterial species from the human gut microbiota. *Sci Transl Med* 9

Morrison ML, Xue KS & Rosenberg NA (2024) Quantifying compositional variability in microbial communities with FAVA. doi:10.1101/2024.07.03.601929 [PREPRINT]
