# Supplementary material for: Common xenobiotics modulate gut microbial responses to low‑calorie sweeteners in vitro
Source: Mol Syst Biol. 2026 Jun 25;22(9):1453–73. doi: 10.1038/s44320-026-00225-6 (PMC13538661; doi:10.1038/s44320-026-00225-6)
Supplement: Supplementary file 2 [file 44320_2026_225_MOESM2_ESM.pdf]

molecular systems biology

Common xenobiotics modulate gut microbial responses to low-calorie sweeteners in vitro

Sonja Blasche, Vinita Periwal, Nonantzin Beristain-Covarrubias, Anna Lindell, Indra Roux, Stephan Kamrad, Simone Mozzachiodi, Rob Bradley, Hilal Ozgur, Bini Ramchandran, Vladimir Benes, and Kiran Patil

Corresponding author(s): Kiran Patil (kp533@cam.ac.uk) , Sonja Blasche (sb2487@cam.ac.uk)

Review Timeline:

Submission Date:	11th Apr 25
Editorial Decision:	28th May 25
Revision Received:	2nd Sep 25
Editorial Decision:	18th Dec 25
Revision Received:	2nd Mar 26
Editorial Decision:	31st Mar 26
Appeal Received:	14th Apr 26
Editorial Decision:	23rd Apr 26
Revision Received:	13th May 26
Accepted:	9th Jun 26

Editor: Poonam Bheda

Transaction Report:

(Note: With the exception of the correction of typographical or spelling errors that could be a source of ambiguity, letters and reports are not edited. Depending on transfer agreements, referee reports obtained elsewhere may or may not be included in this compilation. Referee reports are anonymous unless the Referee chooses to sign their reports.)

28th May 2025

Manuscript Number: MSB-2025-13043
Title: Impact of low-calorie sweeteners on gut bacteria is modulated by common xenobiotics

Dear Prof Patil,

Thank you for the submission of your manuscript to Molecular Systems Biology. We have now received feedback from the three reviewers who agreed to evaluate your manuscript. As you will see from the reports below, the referees acknowledge the interest of the study and are overall supportive of your work; however they also comment on multiple aspects of the manuscript that should be strengthened in a revision.

Without repeating all the comments listed below, some of the more fundamental issues raised are the following:
- Many details of the methods and analyses are missing
- statistical analyses should be improved
- Bliss model should be further justified and validated, and Loewe synergy should be considered as an alternative

All other issues raised would need to be satisfactorily addressed. Please let me know in case you would like to discuss in further detail any of the comments, I would be happy to schedule a call.

When submitting your revised manuscript, please carefully review the instructions that follow below. We perform an initial quality control of all revised manuscripts before re-review; failure to include requested items will delay the evaluation of your revision.

We require:

1) A .docx formatted version of the manuscript text (including legends for main figures, EV figures and tables). Please make sure that the changes are highlighted to be clearly visible. Alternatively you may choose to submit your manuscript as a LaTeX file.

2) Individual production quality figure files as .eps, .tif, .jpg (one file per figure). For guidance, download the 'Figure Guide PDF' (https://www.embopress.org/page/journal/17574684/authorguide#figureformat).

3) At EMBO Press we ask authors to provide source data for the main figures. Our source data coordinator will contact you to discuss which figure panels we would need source data for and will also provide you with helpful tips on how to upload and organize the files.

4) A .docx formatted letter INCLUDING the reviewers' reports and your detailed point-by-point responses to their comments. As part of the EMBO Press transparent editorial process, the point-by-point response is part of the Peer Review File (PRF), which will be published alongside your paper.

5) A complete author checklist, which you can download from our author guidelines (https://www.embopress.org/page/journal/17574684/authorguide#submissionofrevisions). Please insert information in the checklist that is also reflected in the manuscript. The completed author checklist will also be part of the PRF.

6) Please note that all corresponding authors are required to supply an ORCID ID for their name upon submission of a revised manuscript.

7) It is mandatory to include a 'Data Availability' section after the Materials and Methods. Before submitting your revision, primary datasets produced in this study need to be deposited in an appropriate public database, and the accession numbers and database listed under 'Data Availability'. Please remember to provide a reviewer password if the datasets are not yet public (see https://www.embopress.org/page/journal/17574684/authorguide#dataavailability).

In case you have no data that requires deposition in a public database, please state so in this section as follows: "This study includes no data deposited in external repositories". Note that the Data Availability Section is restricted to new primary data that are part of this study.

8) All Materials and Methods need to be described in the main text using our 'Structured Methods' format, which is required for all research articles. According to this format, the Methods section includes a Reagents and Tools Table (listing key reagents, experimental models, software and relevant equipment and including their sources and relevant identifiers) followed by a Methods and Protocols section describing the methods using a step-by-step protocol format. The aim is to facilitate adoption of the methodologies across labs. Please upload the Reagents and Tools table as a separate document when submitting your revised manuscript. More information on how to adhere to this format as well as a downloadable template (.docx) for the

Reagents and Tools Table can be found in our author guidelines:
https://www.embopress.org/page/journal/17444292/authorguide#structuredmethods

An example of a Method paper with Structured Methods can be found here:
https://www.embopress.org/doi/10.15252/msb.20178071.

9) For data quantification: please specify the name of the statistical test used to generate error bars and p-values, the number (n) of independent experiments (specify technical or biological replicates) underlying each data point and the test used to calculate p-values in each figure legend. The figure legends should contain a basic description of n, p-values and the test applied. Graphs must include a description of the bars and the error bars (s.d., s.e.m.). Please provide exact p-values (in either the figure or figure legend).

10) Our journal encourages inclusion of *data citations in the reference list* to directly cite datasets that were re-used and obtained from public databases. Data citations in the article text are distinct from normal bibliographical citations and should directly link to the database records from which the data can be accessed. In the main text, data citations are formatted as follows: "Data ref: Smith et al, 2001" or "Data ref: NCBI Sequence Read Archive PRJNA342805, 2017". In the Reference list, data citations must be labeled with "[DATASET]". A data reference must provide the database name, accession number/identifiers and a resolvable link to the landing page from which the data can be accessed at the end of the reference. Further instructions are available at .

11) We replaced Supplementary Information with Expanded View (EV) Figures and Tables that are collapsible/expandable online. EV Figures should be cited as 'Figure EV1, Figure EV2" etc... in the text and their respective legends should be included in the main text after the legends of regular figures.
- For the figures that you do NOT wish to display as Expanded View figures, they should be bundled together with their legends in a single PDF file called *Appendix*, which should start with a short Table of Content. Appendix figures should be referred to in the main text as: "Appendix Figure S1, Appendix Figure S2" etc.
- Additional Tables/Datasets should be labeled and referred to as Table EV1, Dataset EV1, etc. Legends should be provided in a separate tab in case of .xls files. Alternatively, the legend can be supplied as a separate text file (README) and zipped together with the Table/Dataset file.
See detailed instructions here:
https://www.embopress.org/page/journal/17574684/authorguide#expandedview

12) Author contributions: CRediT has replaced the traditional author contributions section because it offers a systematic machine-readable author contributions format that allows for more effective research assessment. Please remove the Authors Contributions from the manuscript and use the free text boxes beneath each contributing author's name in our system to add specific details on the author's contribution. More information is available in our guide to authors.

13) Disclosure statement and competing interests: We updated our journal's competing interests policy in January 2022 and request authors to consider both actual and perceived competing interests. Please review the policy https://www.embopress.org/competing-interests and update your competing interests if necessary.

14) Every published paper now includes a 'Synopsis' to further enhance discoverability. Synopses are displayed on the journal webpage and are freely accessible to all readers. They include a short stand first (maximum of 300 characters, including space) as well as 2-5 one-sentences bullet points that summarizes the paper. Please write the bullet points to summarize the key NEW findings. They should be designed to be complementary to the abstract - i.e. not repeat the same text. We encourage inclusion of key acronyms and quantitative information (maximum of 30 words / bullet point). Please use the passive voice. Please attach these in a separate file or send them by email, we will incorporate them accordingly.

Please also suggest a striking image or visual abstract to illustrate your article as a PNG file 550 px wide x 300-600 px high.

Please note that these would be the final versions and changes during proofing are usually not allowed.

15) As part of the EMBO Publications transparent editorial process initiative (see our policy here: https://www.embopress.org/transparent-process#Review_Process), Molecular Systems Biology will publish online a Peer Review File (PRF) to accompany accepted manuscripts.
In the event of acceptance, this file will be published in conjunction with your paper and will include the anonymous referee reports, your point-by-point response and all pertinent correspondence relating to the manuscript. Let us know whether you agree with the publication of the PRF and as here, if you want to remove or not any figures from it prior to publication.
Please note that the Author checklist will be published at the end of the PRF.

Molecular Systems Biology has a "scooping protection" policy, whereby similar findings that are published by others during review or revision are not a criterion for rejection. Should you decide to submit a revised version, I do ask that you get in touch after three months if you have not completed it, to update us on the status.

Yours sincerely,

Poonam Bheda, PhD
Scientific Editor
Molecular Systems Biology

Reviewer #1:

The authors report interactions between 25 phylogenetically diverse gut bacterial strains and 39 commercially used sweeteners, by testing these sweeteners individually and in combination with four other compounds. OD600 measurements, 16S rRNA sequencing, proteomics, metabolomics, cytotoxicity, and cytokine assays were performed in this study. While the study benefits from an extensive dataset, the clarity of data analysis and the explanation of the resulting findings should be significantly improved.

Major comments:
1. In abstract, "four commonly co-consumed compounds, viz., advantame, caffeine, vanillin, and duloxetine." It is easily understood that food additive can be commonly co-consumed with sweeteners. However, it is not so obvious for duloxetine. I recommend that the authors either provide supporting data or references to justify duloxetine as a "commonly co-consumed" compound with sweeteners, or alternatively, revise the phrasing to clarify that duloxetine was selected for other experimental reasons.
2. Page 2 lines 5-10, The authors suggest that previous studies on sweeteners' impact on the gut microbiota may not necessarily imply direct effects, highlighting the possibility of emergent effects arising from a community context or mixture effects. While this is an important point, I would like to emphasize that emergent effects themselves represent meaningful biological phenomena, especially in complex microbial ecosystems. In fact, a microbial species' response in isolation may differ substantially from its behavior within a community context, due to interspecies interactions, community-level dynamics, and emergent properties. Therefore, emergent effects should not be interpreted as artifacts or indirect noise, but rather as essential components of the microbiome's functional response to sweeteners. I suggest the authors clarify this distinction and perhaps reframe their argument to acknowledge that both direct and community-mediated (emergent) effects are biologically significant and complementary in understanding microbiome-sweetener interactions.
3. Page 2 Lines 44-46, "Significant sweetener-bacteria interactions ($p<0.05$ and at least 20% change in growth) were re-tested in an independent batch at 25, 50, 100, 200 and 400 μM concentrations." This wasn't very clear to readers how the "significant interactions" were statistically quantified, and how many such significant interactions were determined at additional concentrations?
4. I'm confused by the relationship between Figure 1a and 1b. It shows in Figure 1a that there is 25*39=975 pairs of sweetener-bacteria pairs, this corresponds to the left panel of Figure 1b. However, there seems to be another 25*39*4=3900 sweetener-bacteria-compound triplets, but the right panel of Figure 1b only shows DMSO with the four co- consumed compounds. How is the right panel of Figure 1b obtained?
5. Page 2 Line 47-48, "We observe high overlap between the first and the second experiment (>90% overlap, 30 interactions) (supplementary figures 1-2 ..." is overly concise. Please elaborate on the specific analyses performed and explain how these analyses support the conclusion. This will help readers better understand how the >90% overlap was determined and its significance.
6. Figure 1, please indicate what statistical analyses were performed. Importantly, what statistical model was assumed for the tests?
7. Overall, statistical reporting throughout the manuscript is generally poor. Wherever applicable, all statistical methods should be fully described, including sample sizes, measures of central tendency and variability, the statistical tests used, corrections for multiple comparisons (if applicable), and clear reporting of exact p-values and data distributions. Additionally, the presentation and formatting of statistical results should adhere to standard conventions to ensure clarity and consistency.
8. Page 3 line 4, "the 30 direct interactions identified in this study" is not easy to find. It was only after careful examination that I realized these correspond to the 30 asterisks in the left panel of Figure 1b. The authors should clarify this in the main text as well as in the figure legend to help readers easily locate and understand this key result.
9. The authors claimed that "the 30 direct interactions identified in this study have not been previously reported to our knowledge", however, this reviewer found similar heatmaps to Figure 1b in other studies, such as in PMID: 35695565 (Figure 2), although presented in a genus level resolution. Have the authors compared their findings to such studies and to those stated in Supplementary Table S1? I would be surprised that none of the findings agree with any of the literatures.
10. Page 3 lines 13-15, the statement "We also tested 12 sweetener combinations with commonly used drugs (300 interactions), reflecting typical tablet formulations" appears rather abrupt, as this analysis is not introduced earlier in the manuscript. Moreover, the specific drugs tested are not clearly listed or described, which limits the reader's understanding of the rationale and relevance of this experiment.
11. Page 3, Line 30, the authors state that "Combinations showing antagonistic interactions included vanillin and saccharin,

which are frequently co-used in processed food products (Fig. 1e)." However, based on Figure 1e, there does not appear to be a significant difference between vanillin alone and the vanillin + saccharin combination. It is unclear how the authors concluded that an antagonistic interaction exists.
12. Figure 2b, the p1 passage looked similar to each other, and the changes started from p2. This is confusing to me, why didn't the compounds show impact at p1? How many technical replicates were performed for the 16s analysis?
13. Figure 2d: It is unclear what the five dots in each group represent-are they five different time points or technical replicates? If different time points are mixed, the current analysis is inappropriate. The data should first be stratified by time point, and variability should be assessed among technical replicates at each fixed time point.
14. Figure 2f: The statistical analysis presented is incomplete. To properly demonstrate the synergistic effect of the duloxetine-isosteviol combination, duloxetine and isosteviol should each be compared to the DMSO control individually, and, importantly, duloxetine and isosteviol should also each be compared to the duloxetine-isosteviol combination.
15. Page 6, Line 30: The authors state, "To ensure that the bacteria were at the same growth phase when treated, we pre-grew each species for ~3 hours to allow reaching the logarithmic growth phase." However, based on Supplementary Figure 7, it appears that 3 hours is still within the lag phase for at least P. merdae. The authors should perform growth curve modeling to accurately determine whether the 3-hour time point corresponds to the logarithmic growth phase, and provide clear evidence to support this claim.
16. Supplementary Figure 8 shows that growth phase differences account for much greater variation than compound-induced effects, and compounds do not appear to separate clearly, at least along PC1-PC2. This result is counterintuitive, as one would expect treatment effects to produce larger differences.
17. Figure 3c: Since the authors performed technical triplicates for the proteomics analyses, appropriate statistical analyses should be incorporated into this figure to properly support the findings. It appears that up- and down-regulation are determined solely based on fold changes without stats, which is insufficient.
18. Page 8-9, statistical analysis is also lacking for genetic screening, making any conclusions not reliable. I'm unable to evaluate any biological finding due to such lack of stats.
19. Supplementary figure 13, what do "duloxetine response" and "isosteviol response mean"? How are these quantified, what is the unit of the y axis? How does the difference between sup and wc indicate bioaccumulation? The scientific presentation needs better clarity and precision.
20. In Figure 6, what is a drug control? Similarly, page 13 line 5, "the DMSO control plus 50 μM isosteviol and duloxetine added after harvesting the supernatant. Both the DMSO and the DMSO plus compounds control (drug control)" what is DMSO plus compounds control (drug control)? What are the "compounds" referring to here? If "50 μM isosteviol and duloxetine" is the drugs control, then why on Figure 6 there is also a duloxetine+isosteviol group? Same concentrations of compounds but added at different time points? This is quite unclear leaving readers to guess.
21. Beyond these specific examples raised by this reviewer, it is strongly recommended that the authors carefully re-examine the entire manuscript to identify and revise similar cases of vague terminology or unclear referents.
22. The Discussion section is overly brief and largely disconnected from the specific experimental results and key findings of the study. It should be substantially expanded to better contextualize the data. For instance, the concept of 'mixture effects' is mentioned in both the Introduction and Discussion, but the manuscript does not clearly explain how these effects are supported by the experimental findings presented. What biological insights does the data offer regarding these mixture interactions? Furthermore, the study only examined the mixture effects of sweeteners against four other compounds, which presents a clear limitation in terms of generalizability. The authors should explicitly acknowledge this limitation and discuss how it may constrain the interpretation of their findings.

Minor comments:
1. In supplementary table 1, please write explicitly the first names and last names of all Last author and First author.
2. Please check completeness of references in supplementary table 1, for example, PMID: 35695565 that performs in vitro study of sweeteners on gut microbiome is missing. The authors should perform a throughout literature investigation.
3. Please review the "Phylum" column in Supplementary Table 2. It appears that both old and new taxonomic names are mixed. For instance, "Proteobacteria" is the old nomenclature, "Bacteroidota" is the updated name, and "Bacillota (Firmicutes)" presents both the new and old names simultaneously. Remove the hyperlink behind "Bacillota (Firmicutes)". Use italic font for genus and species names in Supplementary Tables 1 and 2.
4. Page 3 line 12-13, "we screened 156 sweetener-xenobiotic combinations (39 sweeteners and 4 xenobiotics)" 39 and 4 makes 43, making the sentence confusing to read. Revise as "We screened 156 sweetener-xenobiotic combinations, generated by pairing 39 sweeteners with 4 xenobiotics (39 × 4), ... "
5. For Fig 3b and any other PCA, The cord length (axis limits) for PC1 and PC2 should reflect the true data spread with equal scaling, unless otherwise justified, to ensure accurate interpretation of the principal component relationships.

Reviewer #2:

This manuscript presents a systematic, multi-omics exploration of how 39 commercially used sweeteners, alone and in combination with four frequently co-consumed xenobiotics, influence the growth, physiology, and host-relevant outputs of 25 phylogenetically diverse human-gut bacteria. By integrating high-throughput growth profiling, TMT-proteomics, genome-wide Tn-BarSeq, untargeted/targeted metabolomics, a synthetic 25-member community, and host-cell assays, the authors uncover >100 hitherto unknown compound-compound-microbe interactions and delineate a potent synergy between isosteviol and the

antidepressant duloxetine. The breadth of the data, the discovery of mixture effects that propagate from bacterial transporters to altered cytokine release in Caco-2 cells, and the open dissemination of resources fit the remit of MSB.

Overall the manuscript is well written and delivers a large amount of new data, which will advance the field. Overall this reviewer finds the manuscript suitable for publication in MSB after some suggested revisions outlined below.

(1) I suggest the authors tone down the language of 3/4 of sweeteners inhibit gut bacteria and highlight that the majority of significant hits apply to only two gut bacteria: Clostridium symbiosum or Lacrimispora saccharolytica. It is possible that a more sensitive screening method would have found more hits, yet, if these two organisms where left out of the screening set the results would have looked very different. Thus, I suggest the authors update both the first results section and the abstract to highlight this more clearly.

(2) Bliss is the most sensitive synergy measure and one could argue that it is not a "true synergy" as characterized by Loewe synergy. The authors should consider reporting on Loewe synergy in addition to Bliss and discuss the differences.

(3) For the Duloxetine / Isosteviol community experiment it would be good to assess more directly whether the two compounds inhibitor complementary subsets of bacteria or whether there is actual potentiation or synergy in the action against the target bacteria. Overall the claim to synergy in this experiment must also be further qualified ideally through the use of Loewe additivity.

(4) It is not clear to this reviewer how the claim "R. intestinalis showed a strong synergistic effect in monoculture, while P. merdae exhibited an additive effect in monoculture" is supported by Supplementary figure 7. Please provide additional commentary and detail.

(5) Overall the detailed assessment of theDuloxetine / Isosteviol combination could potentially be shortened to focus more on the implications of the findings in a health setting.

(6) On the mamalian cell cytotoxicity (Fig 6a) it seems that the effects observed could be simply additive of the spent media and the presence of the compounds. The authors should discuss this possibility in greater detail.

(7) The cytokine secretion data would point towards an anti-inflammatory action of the drug combination perturbed community. The authors should discuss this in greater detail.

(8) The discussion could be strengthened by a more detailed discussion of the findings from the paper - in particular the limitations around synergy assessments outlined in the previous points, the potential for anti-inflammatory actions and more broadly put this data into context of previous clinical and pre-clinical studies of non caloric sweeteners. Finally, it would be good to discuss any hypothesis for the public health implications of these findings.

Reviewer #3:

1. Introduction and Background:
- The introduction is informative, although I think it could better emphasize the direct impact of low-calorie sweeteners on specific gut microbiota functionalities. For instance, mentioning the specific metabolic changes observed in previous studies would strengthen the argument for examining the effects of sweeteners on microbial metabolic pathways. There is a supplementary summary table, but I feel it would be worth it to directly mention some of these findings in the introduction.

2. Methodology:
- The choice of mGAM as the growth medium is appropriate for the selected strains. However, the authors should elaborate on how this medium's composition might selectively favor certain microbial behaviors. It would be beneficial to compare its efficacy to other media employed in similar studies, supported by relevant citations.

3. Experimental Design:
- While the authors articulate the combinatory approach of isosteviol and duloxetine, they might clarify the rationale behind the dosage selection and include a more detailed exposure timeline to provide insight into timing impacts on microbial responses, further validating results related to microbial growth dynamics.

4. Results - Metabolite Analysis:
- In discussing the results, specify in further depth how the identified metabolites relate to overall gut health and functionality. Furthermore, elaboration on the health implications of elevated glutamine levels and reduced butyric acid levels would enhance the discussion around metabolic alterations caused by these compounds. The overall length of the discussion section also seems too brief relative to the results section.

5. Data Presentation - Statistical Analysis:
- The statistical methods applied are well documented, but the use of the Bliss model requires further clarification. You should detail how this model integrates microbial interactions and what specific assumptions were made when applying it to represent synergy or antagonism appropriately.

6. Discussion - Implications of Findings:
- While the discussion on the effects of sweeteners is well articulated, it often feels speculative. Including recent longitudinal studies demonstrating these health outcomes will support claims (for example, linking sweetener consumption to obesity and cardiovascular diseases). Additionally, balancing health benefits against potential negative outcomes could provide a nuanced understanding.

7. Conclusion - Future Directions:
- The conclusion could more forcefully highlight the implications of the findings regarding the human microbiome and potential therapeutic uses of duloxetine and isosteviol. Suggesting specific mechanisms for further exploration (potentially based on differential protein abundance techniques noted in your results) could pave the way for future research directions.

8. References Assessment and Use:
- The references listed support many of the central concepts. However, expanding the discussion around studies indicating the long-term effects of sweeteners on gut health would provide depth, particularly highlighting earlier studies that discuss how changes in bacterial metabolites might mediate host interactions.

9. Clarifying Limitations:
- I noticed there wasn't much discussion pertaining to potential limitations of the study. It would be prudent to acknowledge confounding factors, such as dietary variances that could skew results. This transparency will help refine future studies and reinforce the validity of your current findings.

10. Biological Context of Cytokines:
- The discussion on IL-6 and IL-8 should be deepened concerning their roles in neutrophil biology. Elucidating the dual nature of their pro- and anti-inflammatory roles will give readers a more robust understanding of how sweeteners might modulate immune responses within the gut context.

We thank the reviewers and editor for their constructive comments. These have greatly helped us to improve the clarity of the manuscript. A point-by-point response to all editorial and referees' comments is provided below.

The original comments are in black font, while our response is in blue font.

Response to editorial comments

Without repeating all the comments listed below, some of the more fundamental issues raised are the following:
- Many details of the methods and analyses are missing
- statistical analyses should be improved
- Bliss model should be further justified and validated, and Loewe synergy should be considered as an alternative

→ All points raised by the reviewers have been addressed as detailed in the point-by-point response below.

Response to referees' comments

Reviewer #1:

The authors report interactions between 25 phylogenetically diverse gut bacterial strains and 39 commercially used sweeteners, by testing these sweeteners individually and in combination with four other compounds. OD600 measurements, 16S rRNA sequencing, proteomics, metabolomics, cytotoxicity, and cytokine assays were performed in this study. While the study benefits from an extensive dataset, the clarity of data analysis and the explanation of the resulting findings should be significantly improved.

We thank the reviewer for their thoughtful and constructive feedback. We appreciate the recognition of the comprehensive dataset and the breadth of experimental approaches employed in our study.

We acknowledge the point regarding the clarity of our data analysis and the explanation of the findings. In response, we have made substantial revisions to improve the organisation and presentation of the data.

- Edited the results section to better guide the reader through the major findings

- Edited the text related to our analytical approaches, including statistical methods and data analysis to highlight key insights derived from multi-omics data

- Revised the discussion section to better link our findings to their broader implications and potential limitations

Major comments:
1. In abstract, "four commonly co-consumed compounds, viz., advantame, caffeine, vanillin, and duloxetine." It is easily understood that food additive can be commonly co-consumed with sweeteners. However, it is not so obvious for duloxetine. I recommend that the authors either provide supporting data or references to justify duloxetine as a "commonly co-consumed" compound with sweeteners, or alternatively, revise the phrasing to clarify that duloxetine was selected for other experimental reasons.

-> Duloxetine is a commonly prescribed serotonin-norepinephrine reuptake inhibitor, marketed under the brand name Cymbalta, among others. In 2022 and 2023, it ranked as the 31st most frequently prescribed drug in the United States, with over 18 million prescriptions and more than 4 million patients (https://clincalc.com/DrugStats/Drugs/Duloxetine). This makes it a relevant compound to test in combination with sweeteners.

Action taken:
We have added the rationale for use of duloxetine to the introduction. The revised text is copied below. We hope this clarifies the rationale behind our selection of duloxetine.

"*Duloxetine is a commonly used antidepressant drug34, which has been shown previously to have significant effects on metabolite secretion in gut microbes35. In the years 2022 and 2023, it was the 31st most prescribed drug with more than 18 million prescriptions and more than 4 million patients in the United States alone (https://clincalc.com/DrugStats/Drugs/Duloxetine).*"

2. Page 2 lines 5-10, The authors suggest that previous studies on sweeteners' impact on the gut microbiota may not necessarily imply direct effects, highlighting the possibility of emergent effects arising from a community context or mixture effects. While this is an important point, I would like to emphasize that emergent effects themselves represent meaningful biological phenomena, especially in complex microbial ecosystems. In fact, a microbial species' response in isolation may differ substantially from its behavior within a community context, due to interspecies interactions, community-level dynamics, and emergent properties. Therefore, emergent effects should not be interpreted as artifacts or indirect noise, but rather as essential components of the microbiome's functional response to sweeteners. I suggest the authors clarify this distinction and perhaps reframe their argument to acknowledge that both direct and community-mediated (emergent) effects are biologically significant and complementary in understanding microbiome-sweetener interactions.

-> We agree that the emergent effects are important and relevant. Indeed, that is why we have done the community experiment shown in Figure 2. We have recently shown that indirect effects can be mediated by metabolite secretion and/or changes in bioaccumulation (Garcia-Santamarina et al 2024: https://doi.org/10.1016/j.cell.2024.08.037). This is why we have done metabolomics experiments and the forward genetic screen (Figure 4).

Action taken: To mechanistically understand sweeteners/compound effect on microbiome it is essential whether effects are direct or indirect. We have now clarified by revising the text (copied below)

“These studies may imply either direct effects or indirect, emergent, effects that may arise in a community context as observed in the case of therapeutic drugs[29], or from mixture effects involving co-consumed foods or compounds. Analysis of direct effects of sweeteners on bacteria are needed to understand the mechanisms through which these compounds impact microbiota changes.”

3. Page 2 Lines 44-46, "Significant sweetener-bacteria interactions ($p<0.05$ and at least 20% change in growth) were re-tested in an independent batch at 25, 50, 100, 200 and 400 μM concentrations." This wasn't very clear to readers how the "significant interactions" were statistically quantified, and how many such significant interactions were determined at additional concentrations?

-> The text is revised for clarity as below.

*“The sweetener-bacteria interactions were tested in two independent experiments whereby the interactions meeting significance thresholds ($p<0.05$ and ≥20% growth change) in the first screen were retested across five concentrations (25-400 μM). True positive interactions were assessed as those with significance (Welch's t-test, $p<0.05$) in both experiments and with consistent effects at ≥2 concentrations (Table EV4d). We observe high overlap between the two experiments, i.e. the initial screen and the second experiment at multiple concentrations (>90% overlap, 30 out of 33 interactions, Appendix figures S1-S2, Tables EV4a-d). Thus, in total we identified 30 interactions between 5 bacterial strains and 26 sweeteners and sugar substitutes (marked with * in Fig. 1b).*

While some direct sweetener-bacteria interactions such as between stevia glycosides and lactobacilli have been described[40] (overview in Table EV1), the 30 direct interactions identified in this study have not been previously reported to our knowledge. Clostridium symbiosum or Lacrimispora saccharolytica were the most sensitive species being affected by many compounds. On the sweetener side, isosteviol was the most potent compound, inhibiting the growth of three bacteria (C. symbiosum, Odoribacter splanchnicus, and Parabacteroides distasonis) while promoting the growth of one, Lactobacillus gasseri.”

4. I'm confused by the relationship between Figure 1a and 1b. It shows in Figure 1a that there is 25*39=975 pairs of sweetener-bacteria pairs, this corresponds to the left panel of Figure 1b. However, there seems to be another 25*39*4=3900 sweetener-bacteria-compound triplets, but the right panel of Figure 1b only shows DMSO with the four co-consumed compounds. How is the right panel of Figure 1b obtained?

-> Thank you for indicating this potential unclarity. The right panel of Figure 1b shows the effect of the four compounds on the gut microbes, when tested alone. This data is used to calculate the interaction effect. The data for the 3900 three-node interactions (sweetener-compound-bacteria) is provided in the supplementary table 4a, while Figure 1c shows the subset of the data where we identify significant interactions.

Action taken: We have revised figure 1 and description for clarity.

5. Page 2 Line 47-48, "We observe high overlap between the first and the second experiment (>90% overlap, 30 interactions) (supplementary figures 1-2 ..." is overly concise.

Please elaborate on the specific analyses performed and explain how these analyses support the conclusion. This will help readers better understand how the >90% overlap was determined and its significance.

-> The reproducibility between initial screen hits and retest results was assessed by Welch's t-test. A hit was considered validated only if it demonstrated statistical significance ($p<0.05$) and consistent effects in both independent screens and the retest (significant for ≥ 2 concentrations).

Action taken: We have expanded the text for additional clarity:
*" The sweetener-bacteria interactions were tested in two independent experiments whereby the interactions meeting significance thresholds ($p<0.05$ and ≥20% growth change) in the first screen were retested across five concentrations (25-400 µM). True positive interactions were assessed as those with significance (Welch's t-test, $p<0.05$) in both experiments and with consistent effects at ≥2 concentrations (supplementary table 4d). We observe high overlap between the two experiments, i.e. the initial screen and the second experiment at multiple concentrations (>90% overlap, 30 out of 33 interactions, supplementary figures 1-2, Supplementary tables 4a-d). Thus, in total we identified 30 interactions between 5 bacterial strains and 26 sweeteners and sugar substitutes (marked with * in Fig. 1b)."*

6. Figure 1, please indicate what statistical analyses were performed. Importantly, what statistical model was assumed for the tests?

-> Action taken: We have expanded the figure caption indicating what statistical test used:

"***Fig. 1. Interactions between sweeteners and gut bacteria. (a)*** *Schematic overview of the sweetener-bacteria screening set-up.* ***(b)*** *Identified interactions between sweeteners and human gut bacteria. All compounds were tested at 50 µM concentration. Interactions were deemed significant (marked with *) when, 1) bh-corrected[93] p-value <0.05, 2) > 20% change in growth (area under the growth curve, AUC) compared to the solvent control, and 3) confirmed in an independent experiment including additional concentrations as described in the main text.* ***(c)*** *Significant sweeteners-xenobiotic interactions impacting gut bacterial growth. Bliss score for the significant interactions (bh-corrected p-value<0.05, and absolute (log2 fold change) >0.32) are shown in the colormap, see supplementary table 4a for full screening results.* ***(d)*** *Example of synergistic interaction between isosteviol and duloxetine affecting growth of R. intestinalis (DMSO solvent control vs duloxetine and isosteviol: p-val=9.10218E-40* ***(e)*** *Example of an antagonistic interaction between saccharin and vanillin affecting growth of B. adolescentis*, *p-val =0.020376*. If not stated otherwise, significance was determined using Welch's t-test. * p-val<0.05, ** p-val <0.01, *** p-val <0.001, **** p-val <0.0001*"

7. Overall, statistical reporting throughout the manuscript is generally poor. Wherever applicable, all statistical methods should be fully described, including sample sizes, measures of central tendency and variability, the statistical tests used, corrections for multiple comparisons (if applicable), and clear reporting of exact p-values and data distributions. Additionally, the presentation and formatting of statistical results should adhere to standard conventions to ensure clarity and consistency.

-> Thank you for the feedback, we have gone through the manuscript and added information where missing. To reduce crowding in the main text and figures, exact p-values are provided in the corresponding supplementary tables. This is necessitated by the high-throughput nature of many of our experiments.

8. Page 3 line 4, "the 30 direct interactions identified in this study" is not easy to find. It was only after careful examination that I realized these correspond to the 30 asterisks in the left panel of Figure 1b. The authors should clarify this in the main text as well as in the figure legend to help readers easily locate and understand this key result.

-> We have revised text and figure legend accordingly.

9. The authors claimed that "the 30 direct interactions identified in this study have not been previously reported to our knowledge", however, this reviewer found similar heatmaps to Figure 1b in other studies, such as in PMID: 35695565 (Figure 2), although presented in a genus level resolution. Have the authors compared their findings to such studies and to those stated in Supplementary Table S1? I would be surprised that none of the findings agree with any of the literatures.

-> Thank you for your feedback. The study referenced (PMID: 35695565) is already cited in our manuscript. While we initially omitted it from the supplementary table to avoid redundancy, we have now included it as per your suggestion.

Regarding the comparison at the genus level, we believe it would not provide additional insight, as it is well-established that there are immense differences even between strains regarding genetic makeup, functional capacity and the effects of xenobiotics on bacteria (e.g. PMID: 34497420, 29555994, 38271302, 25640238. Species-level differences are even more pronounced. Furthermore, our study specifically investigates the direct effects of sweeteners on bacterial strains, whereas the study in PMID: 35695565 focuses on community-level interactions, where the effects may be indirect, as the reviewer previously noted. A systematic mono-culture study is therefore essential to identify and interpret any emergent interactions as we have shown in this study using a synthetic community.

We hope this clarifies our approach and the distinction between the two studies.

10. Page 3 lines 13-15, the statement "We also tested 12 sweetener combinations with commonly used drugs (300 interactions), reflecting typical tablet formulations" appears rather abrupt, as this analysis is not introduced earlier in the manuscript. Moreover, the specific drugs tested are not clearly listed or described, which limits the reader's understanding of the rationale and relevance of this experiment.

-> Action taken: We have added the following in the introduction:

“In addition, we tested combinations of sweeteners with commonly used tablet-formulated drugs—acetaminophen, aripiprazole, cetirizine, ethinylestradiol, ibuprofen, montelukast, ranitidine, and risperidone—which are typically co-formulated with sweeteners to mask bitterness.”

Further we have added a column to supplementary table 5a listing links/publications mentioning the sweetener-drug combinations tested here (header: Example for co-formulation).

11. Page 3, Line 30, the authors state that "Combinations showing antagonistic interactions included vanillin and saccharin, which are frequently co-used in processed food products (Fig. 1e)." However, based on Figure 1e, there does not appear to be a significant difference between vanillin alone and the vanillin + saccharin combination. It is unclear how the authors concluded that an antagonistic interaction exists.

-> The original presentation of Figure 1d and e did not adequately highlight the statistical significance. We have revised the figure to emphasize comparisons between the DMSO control and each treatment. The updated data clearly show that vanillin and saccharin alone do not differ significantly from the control, whereas their co-treatment results in a significant effect (DMSO control vs. vanillin+saccharin p= 0.020375863).

All p-values and data can be found in the source data for Figure 1d.

12. Figure 2b, the p1 passage looked similar to each other, and the changes started from p2. This is confusing to me, why didn't the compounds show impact at p1? How many technical replicates were performed for the 16s analysis?

-> Microbial community assembly typically requires 3–5 passages to stabilize (PMID: 35051349; van den Berg et al., https://doi.org/10.1101/2024.04.29.591619). During early passages, especially in complex communities, dynamics are primarily shaped by interspecies interactions rather than external perturbations (PMID: 38486074), which may explain the limited effect at p1, where the community is still in the process of stabilization. For the 16S analysis, we performed four replicates total—2 biological replicates, each with 2 technical replicates.

13. Figure 2d: It is unclear what the five dots in each group represent-are they five different time points or technical replicates? If different time points are mixed, the current analysis is inappropriate. The data should first be stratified by time point, and variability should be assessed among technical replicates at each fixed time point.

-> Each dot represents an individual passage. This comparison enables a global assessment of differences between treatments.

Action taken: As suggested by the reviewer, we have revised the analysis to include all replicates in the figure. Passages are now distinguishable by shape. The underlying Shannon diversity data used in Fig. 2d, including values per treatment, passage, and each of the four replicates, are provided in Supplementary Table 7a. For clarity, we have also referenced Supplementary Table 7a in the figure legend.

14. Figure 2f: The statistical analysis presented is incomplete. To properly demonstrate the synergistic effect of the duloxetine-isosteviol combination, duloxetine and isosteviol should

each be compared to the DMSO control individually, and, importantly, duloxetine and isosteviol should also each be compared to the duloxetine-isosteviol combination.

-> Thank you for your suggestion. To maintain clarity and avoid overcrowding, we have included statistical significance only in the main figure, as referenced in the text. However, the results of all comparisons are provided in the supplementary materials. Specifically, supplementary table 8 presents all comparisons for each passage, treatment, and strain (Student's t-test).

15. Page 6, Line 30: The authors state, "To ensure that the bacteria were at the same growth phase when treated, we pre-grew each species for ~3 hours to allow reaching the logarithmic growth phase." However, based on Supplementary Figure 7, it appears that 3 hours is still within the lag phase for at least P. merdae. The authors should perform growth curve modeling to accurately determine whether the 3-hour time point corresponds to the logarithmic growth phase, and provide clear evidence to support this claim.

-> Thank you for your comment. The growth curve in supplementary figure 7 was included as a reference for compound inhibition, conducted at a total volume of 100 µL and starting at an OD of 0.05. For the proteomics experiments, cultures were initiated from a preculture, and the OD was not adjusted at the start. Therefore, OD, rather than time, was used to determine when bacteria reached the logarithmic phase. For both bacterial strains, the log phase was achieved after approximately 2.5 and 3 hours in a 150 mL culture (see OD values in supplementary Tables 11 and 12).

Action taken: We have revised the relevant section to clarify the methodology and address potential ambiguities.

"To investigate the molecular basis of the isosteviol–duloxetine interaction, we analysed proteomic changes in R. intestinalis and P. merdae following exposure to each compound individually and in combination. R. intestinalis was selected as it exhibited a strong synergistic response in monoculture. While P. merdae showed a weaker effect that did not meet the criteria for a Bliss interaction in our screen (BH-corrected $p < 0.05$ and ($\log_2$ fold change) > 0.32; supplementary figure 7), it displayed a significant interaction effect in the synthetic community (Fig. 2f). To control for proteomic changes due to growth-phase differences, both species were pre-cultured to reach logarithmic growth before 4 hours of compound exposure (Fig. 3a, Methods). This approach minimised growth-related variability, which can exceed that induced by compound treatment (supplementary figure 8)."

16. Supplementary Figure 8 shows that growth phase differences account for much greater variation than compound-induced effects, and compounds do not appear to separate clearly, at least along PC1-PC2. This result is counterintuitive, as one would expect treatment effects to produce larger differences.

-> Substantial physiological differences between growth phases in *E. coli* and other bacteria have been well documented, examples are PMID: 34032011 and 28371138. As the proteome of *E. coli* and other bacteria has shown to be responding to stresses and different growth phases, we expected to see such differences.

17. Figure 3c: Since the authors performed technical triplicates for the proteomics analyses, appropriate statistical analyses should be incorporated into this figure to properly support the findings. It appears that up- and down-regulation are determined solely based on fold changes without stats, which is insufficient.

-> Proteomics was performed in biological triplicates, not technical; each replicate was treated separately starting from preculture inoculation. Statistical significance was also included in identifying the differentially abundant proteins. This has been now clarified in the manuscript.

Action taken: We have redesigned figure 3c and added a star for every protein and condition where abundance was significantly changed (ANOVA, bh-adj. p-val <0.05. The source data for these figures can be found in " 20250730_Pmerdae-hits3b.csv " (P. merdae) and " 20250730_Rintestinalis-hits3b.csv " (R. intestinalis). Further we have added the following paragraph to methods:

"Quantitative comparisons and statistical analyses were performed using Proteome Discoverer workflows. The S/N values of TMT reporter ions of the identified peptides, that are unique to the representative proteins, were used to represent the summed protein abundance. The TMT channel with the highest total protein abundance was used as the reference channel for normalization. The normalized abundance values were used for quantitative comparison across sample sets. Protein ratios were calculated from normalized grouped abundances, comparing test and control groups. Fold changes were represented by the median abundance across replicates. Statistical significance was assessed using ANOVA at the individual protein level, with adjusted p-values computed using the Benjamini-Hochberg correction to control the false discovery rate in this large-scale analysis."

18. Page 8-9, statistical analysis is also lacking for genetic screening, making any conclusions not reliable. I'm unable to evaluate any biological finding due to such lack of stats.

-> Statistical analysis was used but apparently was unclear. We have now further clarified the statistical method and the naming of the statistic output columns from the genetic screen in supplementary table 15:

In methods: *“P-value was calculated with TRANSIT resampling set at 20000 permutations and adjusted for multiple comparisons with Benjamini-Hochberg procedure 84”*

Added in supplementary table description: *“P-values (p-value_*condition* columns) were calculated using TRANSIT resampling. Adjusted p-values (Adj_p_value_*condition* columns) were adjusted for multiplicity by BH method.”*

19. Supplementary figure 13, what do "duloxetine response" and "isosteviol response mean"? How are these quantified, what is the unit of the y axis? How does the difference between sup and wc indicate bioaccumulation? The scientific presentation needs better clarity and precision.

-> The duloxetine and isosteviol responses refer to peak area detected by targeted LC-MS for each respective compound.

Under the tested conditions (mGAM, 37 °C, 24 h), neither isosteviol nor duloxetine is biotransformed or degraded by the bacteria, allowing re-extraction of the full amount added. If bioaccumulation occurs, we expect the compound to be retained intracellularly rather than in the supernatant. By comparing measurements from the whole culture (WC) and the supernatant (SUP), we can infer the extent of bioaccumulation. This method of determining compound bioaccumulation has been established in our lab previously (PMID: 34497420).

Action taken: To improve clarity, we have updated the y-axis labels to 'peak area duloxetine' and 'peak area isosteviol'.

20. In Figure 6, what is a drug control? Similarly, page 13 line 5, "the DMSO control plus 50 μM isosteviol and duloxetine added after harvesting the supernatant. Both the DMSO and the DMSO plus compounds control (drug control)" what is DMSO plus compounds control (drug control)? What are the "compounds" referring to here? If "50 μM isosteviol and duloxetine" is the drugs control, then why on Figure 6 there is also a duloxetine+isosteviol group? Same concentrations of compounds but added at different time points? This is quite unclear leaving readers to guess.

-> We agree that this may cause some confusion. The drug control refers to the supernatant from the mock-treated microbial community (DMSO treated) to which 50 μM of each isosteviol and duloxetine were added after harvesting. In contrast, for the duloxetine + isosteviol group, the compounds were added directly to the bacterial community, exposing the bacteria to the compounds. This distinction is crucial for comparing the effects of the community supernatant alone, the community supernatant with compounds, and the compound-treated community on human cell lines.

Action taken: We have revised the figure caption and main text for increased clarity.

21. Beyond these specific examples raised by this reviewer, it is strongly recommended that the authors carefully re-examine the entire manuscript to identify and revise similar cases of vague terminology or unclear referents.

-> We have revised the manuscript for increased clarity and tracked the changes made.

22. The Discussion section is overly brief and largely disconnected from the specific experimental results and key findings of the study. It should be substantially expanded to better contextualize the data. For instance, the concept of 'mixture effects' is mentioned in both the Introduction and Discussion, but the manuscript does not clearly explain how these effects are supported by the experimental findings presented. What biological insights does the data offer regarding these mixture interactions? Furthermore, the study only examined the mixture effects of sweeteners against four other compounds, which presents a clear limitation in terms of generalizability. The authors should explicitly acknowledge this limitation and discuss how it may constrain the interpretation of their findings.

-> We have revised the manuscript and especially the discussion section explaining how the experimental findings support mixture effects and discussed the limitations of this study.

Minor comments:
1. In supplementary table 1, please write explicitly the first names and last names of all Last author and First author.

-> As the PMID listed in table one is a unique identifier of the article, we have removed the columns containing authors names and only kept PMID, Year and Title as identifiers. This is sufficient to identify the respective articles.

2. Please check completeness of references in supplementary table 1, for example, PMID: 35695565 that performs in vitro study of sweeteners on gut microbiome is missing. The authors should perform a throughout literature investigation.

-> PMID: 35695565 has been cited in the main text. To avoid duplication, we did not previously add it to the supplementary. We have added it now.

3. Please review the "Phylum" column in Supplementary Table 2. It appears that both old and new taxonomic names are mixed. For instance, "Proteobacteria" is the old nomenclature, "Bacteroidota" is the updated name, and "Bacillota (Firmicutes)" presents both the new and old names simultaneously. Remove the hyperlink behind "Bacillota (Firmicutes)". Use italic font for genus and species names in Supplementary Tables 1 and 2.

-> Thank you for pointing that out we have corrected the bacterial nomenclature.

4. Page 3 line 12-13, "we screened 156 sweetener-xenobiotic combinations (39 sweeteners and 4 xenobiotics)" 39 and 4 makes 43, making the sentence confusing to read. Revise as "We screened 156 sweetener-xenobiotic combinations, generated by pairing 39 sweeteners with 4 xenobiotics (39 × 4), ... "

-> Thank you for the comment we have edited the text accordingly.

5. For Fig 3b and any other PCA, The cord length (axis limits) for PC1 and PC2 should reflect the true data spread with equal scaling, unless otherwise justified, to ensure accurate interpretation of the principal component relationships.

-> Done.

Reviewer #2:

This manuscript presents a systematic, multi-omics exploration of how 39 commercially used sweeteners, alone and in combination with four frequently co-consumed xenobiotics, influence the growth, physiology, and host-relevant outputs of 25 phylogenetically diverse human-gut bacteria. By integrating high-throughput growth profiling, TMT-proteomics, genome-wide Tn-BarSeq, untargeted/targeted metabolomics, a synthetic 25-member community, and host-cell assays, the authors uncover >100 hitherto unknown compound-compound-microbe interactions and delineate a potent synergy between isosteviol and the antidepressant duloxetine. The breadth of the data, the discovery of mixture effects that propagate from bacterial transporters to altered cytokine release in Caco-2 cells, and the open dissemination of resources fit the remit of MSB.

Overall the manuscript is well written and delivers a large amount of new data, which will advance the field. Overall this reviewer finds the manuscript suitable for publication in MSB after some suggested revisions outlined below.

-> We thank the reviewer for detailed and thoughtful comments. We appreciate the encouraging recognition of our systematic, multi-omics approach and the novel findings, particularly regarding the synergy between isosteviol and duloxetine in compound-compound-microbe interactions.

We have addressed the suggestions in the revision as detailed below.

(1) I suggest the authors tone down the language of 3/4 of sweeteners inhibit gut bacteria and highlight that the majority of significant hits apply to only two gut bacteria: Clostridium symbiosum or Lacrimispora saccharolytica. It is possible that a more sensitive screening method would have found more hits, yet, if these two organisms where left out of the screening set the results would have looked very different. Thus, I suggest the authors update both the first results section and the abstract to highlight this more clearly.

-> We agree with the reviewer in that the hits are ascribed to mostly two species and without these two included the results would different. On the other side, had we included more species, say around 40 or even 100, the statistics again could look different.

To address this, we have modified the abstract to state explicitly that the results apply to only tested bacteria.

“Three quarters of the tested sweeteners individually impacted growth of at least one ***tested*** *bacterial strain.”*

Further we have changed the header of respective results section toa more neutral:
" Impact of sweeteners on bacterial growth "

We hope this clarifies that our results are specific to the 25 species tested, thereby addressing the raised concern.

(2) Bliss is the most sensitive synergy measure and one could argue that it is not a "true

synergy" as characterized by Loewe synergy. The authors should consider reporting on Loewe synergy in addition to Bliss and discuss the differences.

-> Bliss independence and Loewe additivity models have some key differences between based on their underlying assumptions.
https://pubmed.ncbi.nlm.nih.gov/30881603/
https://pubmed.ncbi.nlm.nih.gov/24492921/

	**Bliss independence**	**Loewe additivity**
Assumes same Mode of action	No, both compounds' actions/mechanisms are assumed to be mutually exclusive	Yes, both compounds share similar target or mode of action
Interaction type	Independent	Dose equivalence
Calculation basis	Effect levels (%)	Dose ratios

In this study, we have tested the combinations at one single concentration of 50uM for both compounds. It is also unlikely that all tested compounds will have the same target/mode of action. Thus, Bliss model of independence is the appropriate model to estimate synergy in this study. A statistical determination of synergy was calculated as described in Demidenko et al (PMID: 31765385). Previous studies in compound interaction effects on microbes have also used Bliss model (https://doi.org/10.1038/s41586-018-0278-9).

(3) For the Duloxetine / Isosteviol community experiment it would be good to assess more directly whether the two compounds inhibitor complementary subsets of bacteria or whether there is actual potentiation or synergy in the action against the target bacteria. Overall the claim to synergy in this experiment must also be further qualified ideally through the use of Loewe additivity.

-> Thanks for the insightful comment. While constructing defined bacterial communities with complementary subsets lies beyond the scope of this study, we agree that species-specific analyses are informative. To this end, we conducted ANOVA for each species across treatments and passages (supplementary table 10). This revealed significant differential responses in several strains, including *P. merdae* and *S. copri*, indicating that the treatment with the compounds selectively affects community members. However, given the complexity of microbial communities, secondary effects—such as interspecies interactions—likely contribute (e.g. DOI: 10.1016/j.cell.2024.08.037), and the observed responses cannot be fully attributed to direct effects on individual species. For Bliss vs. Loewe, please see also our answer to the previous comment.

(4) It is not clear to this reviewer how the claim "R. intestinalis showed a strong synergistic effect in monoculture, while P. merdae exhibited an additive effect in monoculture" is supported by Supplementary figure 7. Please provide additional commentary and detail.

-> The effect on *R. intestinalis* showed a significant Bliss score and more than 20% growth inhibition. Although *P. merdae* exhibited a significant Bliss score, it did not reach the 20% inhibition threshold. supplementary figure 7 was included to highlight the growth differences between treatments.

Action taken: We have revised the text and supplementary files to clarify this:

"To investigate the molecular basis of the isosteviol–duloxetine interaction, we analysed proteomic changes in R. intestinalis and P. merdae following exposure to each compound individually and in combination. R. intestinalis was selected as it exhibited a strong synergistic response in monoculture. While P. merdae showed a weaker effect that did not meet the criteria for a Bliss interaction in our screen (BH-corrected $p < 0.05$ and (log_2 fold change) > 0.32; Appendix figure S7), it displayed a significant interaction effect in the synthetic community (Fig. 2f). To control for proteomic changes due to growth-phase differences, both species were pre-cultured to reach logarithmic growth before 4 hours of compound exposure (Fig. 3a, Methods). This approach minimised growth-related variability, which can exceed that induced by compound treatment (Appendix figure S8)."

(5) Overall the detailed assessment of the Duloxetine / Isosteviol combination could potentially be shortened to focus more on the implications of the findings in a health setting.

-> Thank you for the suggestion. We have now revised the discussion including potential implications for microbiome-host interactions.

(6) On the mamalian cell cytotoxicity (Fig 6a) it seems that the effects observed could be simply additive of the spent media and the presence of the compounds. The authors should discuss this possibility in greater detail.

-> Indeed, our "drug control" refers to the supernatant from the vehicle-treated microbial community, into which 50 µM of both isosteviol and duloxetine were added after harvesting. This control accounts for any additive effects resulting from the combined presence of the compounds and the microbial supernatant.

Action taken: We have revised Fig. 6, the figure caption and main text for increased clarity.

*“Fig. 6. Metabolic response of gut bacterial community to isosteviol-duloxetine combination impacts cytotoxicity and cytokine secretion. (a) Spent medium of duloxetine and isosteviol co-treated communities show increased toxicity in HeLa cells, but not in (b) Caco-2 cells. And secretion of the cytokines (c) interleukin-6 (IL-6), (d) interleukin-8 (IL-8) and (e) the interferon-γ inducible protein 10 kDa (CXCL10 or IP-10) by Caco-2 cells as a response to contact with spent medium. IL-6 and IL-8 secretion is significantly reduced for the supernatant of duloxetine and isosteviol co-treated communities, as compared to the DMSO and drug control. The 25-strain bacterial community was treated with duloxetine (Dulox), isosteviol (Isostev), or their combination. Supernatants were harvested and applied to human cell lines. Controls included: (1) a DMSO-treated community (0.2% solvent control), and (2) supernatant from the DMSO-treated community supplemented post-harvest with 50 µM duloxetine and isosteviol (drug control). N>6, significance was determined by Welch's t-test. *p-val <0.05, ***p-val <0.001, UT untreated cells.”*

(7) The cytokine secretion data would point towards an anti-inflammatory action of the drug combination perturbed community. The authors should discuss this in greater detail.

-> We thank the reviewer for pointing this out. We agree that the effect described could be taken as anti-inflammatory. However, considering the sentinel role of the gut and the constant exposure to exogenous stimuli, maintaining the appropriate level of cytokine production and immune cell recruitment is crucial to preserve its integrity. Given the complexity and dynamic interplay between the host's inflammatory response and the gut microbiota, predicting the effects of the in vitro observed changes is challenging. Nonetheless, since IL-6 and IL-8 play dual roles by exhibiting both pro- and anti-inflammatory effects depending on the context, the consequences of their modulation for host immunity will depend on the individual's health status.

Action taken: We have revised the discussion to place greater emphasis on this aspect:

"In the case of duloxetine and isosteviol, our study shows that their co-administration significantly alters the growth of several beneficial gut bacteria compared to either compound alone. This combination also impacts microbial community composition, metabolite secretion, cytotoxicity of microbial metabolites, and cytokine secretion by Caco-2 cells. Notably, it affects key microbiota-derived metabolites involved in host–microbiome interactions, such as butyrate and propionate. Within the human gastrointestinal tract, SCFAs generated by the gut microbiome are in situ utilised by colonocytes, with a minimal amount reaching the liver and even fewer entering the systemic circulation. Butyrate represents 15% of the gut bacteria-derived SCFAs and is used as a main source of energy by colonocytes[79]. Meanwhile, propionate contributes to gluconeogenesis and lipid metabolism of the colonic cells80, strengthening the gut barrier integrity. The decrease observed in these two microbial metabolites after treating the bacterial community with duloxetine and isosteviol may be linked to the suppressive effect on IL-8 and IL-6 secretion by Caco-2 cells.

While our data indicate that a combination of butyrate and propionate can stimulate IL-8 secretion, this alone does not fully account for the effects observed with the community supernatant (Appendix figure S23d). However, bacterial-derived butyrate influences the differentiation and inflammatory profile of mononuclear cells and colonic mucosal cells. In the absence of tissue damage, butyrate helps maintain the appropriate number of "patrolling monocytes" and inflammatory molecules, notably IL-6, IL-8, IL-1beta, and TNF, which are necessary to limit pathogen proliferation and invasion, thereby supporting intestinal integrity and repair in homeostasis[79]. Conversely, propionate regulates the secretion of antimicrobial peptides by intestinal epithelial cells and the expression of cell receptors associated with IL-8 and CXCL10 secretion. IL-8 is a potent neutrophil chemoattractant that also mediates oxidative burst, both of which are crucial in immune responses against pathogens[79]. Thus, the reduction of IL-6 and IL-8 induced by supernatants from communities treated with duloxetine and isosteviol indicates a potential dysregulation of inflammatory signalling that could impair host immune responses to pathogens. The gut functions as a sentinel organ under constant "alert" in basal conditions, requiring finely tuned regulation of complex interactions among intestinal epithelial cells, microbiota, metabolite production, cell receptor expression, and cytokine secretion[81]. Given the complexity and dynamic interplay between the host's inflammatory response and the gut microbiota, predicting the effects of the

discussed changes is challenging. Nonetheless, since IL-6 and IL-8 play dual roles by exhibiting both pro- and anti-inflammatory effects depending on the context, the consequences of their modulation for host immunity will depend on the individual's health status."

(8) The discussion could be strengthened by a more detailed discussion of the findings from the paper - in particular the limitations around synergy assessments outlined in the previous points, the potential for anti-inflammatory actions and more broadly put this data into context of previous clinical and pre-clinical studies of non caloric sweeteners. Finally, it would be good to discuss any hypothesis for the public health implications of these findings.

-> We have revised the discussion section considering the reviewer's recommendation. We would like to be cautious regarding implications for public health given the in vitro nature of our data.

Reviewer #3:

1. Introduction and Background:
- The introduction is informative, although I think it could better emphasize the direct impact of low-calorie sweeteners on specific gut microbiota functionalities. For instance, mentioning the specific metabolic changes observed in previous studies would strengthen the argument for examining the effects of sweeteners on microbial metabolic pathways. There is a supplementary summary table, but I feel it would be worth it to directly mention some of these findings in the introduction.

-> Thank you for the suggestion. We have revised the introduction to include more of the studies mentioned in supplementary table 1. The edited paragraph is provided below.

"Previous studies examining the impact of sweeteners on the gut microbiota and gut bacteria [9,22,27] suggest that these effects are widespread across different bacterial phylogenetic groups. This includes compound-specific effects, such as a product-specific increase in SCFA production for six human samples[9]. Moreover, it has been demonstrated that the consumption of select sweeteners, when combined with a high-fat diet, can alter the composition of the gut microbiota in mice and rats, thereby affecting both microbiota dynamics and host metabolic interactions[16,17,28,29]. Additionally, exposure to sweeteners has been shown to alter fermentation profiles and alter metabolism in vitro, indicating a connection between sweetener exposure and modifications in both microbiota composition and microbial metabolic processes[19]. Table EV1 summarizes a variety of prior in vivo and in vitro studies. These studies may imply either direct effects or indirect, emergent, effects that may arise in a community context as observed in the case of therapeutic drugs[30], or from mixture effects involving co-consumed foods or compounds. Analysis of direct effects of sweeteners on bacteria are needed to understand the mechanisms through which these compounds impact microbiota changes."

2. Methodology:
- The choice of mGAM as the growth medium is appropriate for the selected strains. However, the authors should elaborate on how this medium's composition might selectively favor certain microbial behaviors. It would be beneficial to compare its efficacy to other media employed in similar studies, supported by relevant citations.

->

We have revised the discussion substantially including the below sentence:

"Moreover, the choice of growth medium can influence microbial growth dynamics and interaction outcomes, potentially limiting the generalizability and sensitivity of in vitro findings[21]."

3. Experimental Design:
- While the authors articulate the combinatory approach of isosteviol and duloxetine, they might clarify the rationale behind the dosage selection and include a more detailed exposure timeline to provide insight into timing impacts on microbial responses, further validating results related to microbial growth dynamics.

-> The median concentration of sweeteners in the human colon was estimated to be ~ 49.6µM (supplementary table 3b). Growth of each of gut bacteria was measured hourly for a exposure of 24 hours and the area under the curve was calculated for each tested compound and combination (supplementary table 4a).

Action taken: We have revised the methods section for clarity

4. Results - Metabolite Analysis:
- In discussing the results, specify in further depth how the identified metabolites relate to overall gut health and functionality. Furthermore, elaboration on the health implications of elevated glutamine levels and reduced butyric acid levels would enhance the discussion around metabolic alterations caused by these compounds. The overall length of the discussion section also seems too brief relative to the results section.

-> Thank you for the comment, we have expanded the discussion including suggested topics.

1. We have discussed the role of SCFAs and gut barrier integrity:
"Notably, it affects key microbiota-derived metabolites involved in host–microbiome interactions, such as butyrate and propionate. Within the human gastrointestinal tract, SCFAs generated by the gut microbiome are in situ utilised by colonocytes, with a minimal amount reaching the liver and even fewer entering the systemic circulation. Butyrate represents 15% of the gut bacteria-derived SCFAs and is used as a main source of energy by colonocytes. Meanwhile, propionate contributes to gluconeogenesis and lipid metabolism of the colonic cells, strengthening the gut barrier integrity. The decrease observed in these two microbial metabolites after treating the bacterial community with duloxetine and isosteviol may be linked to the suppressive effect on IL-8 and IL-6 secretion by Caco-2 cells."

2. We have further added the following sentence to the results and discussion section 'Co-exposure impacts primary metabolism in *R. intestinalis* and *P. merdae*:

"Although it remains unclear to what extent increased glutamine production by R. intestinalis contributes to total glutamine levels in the colon, it has previously been shown that increased gut glutamine levels can be beneficial (anti-inflammatory) and detrimental (among others, increased risk of digestive system cancers)[57-59]."

5. Data Presentation - Statistical Analysis:
- The statistical methods applied are well documented, but the use of the Bliss model requires further clarification. You should detail how this model integrates microbial interactions and what specific assumptions were made when applying it to represent synergy or antagonism appropriately.

-> We have added more detailed explanation to supplementary figure 3 (details of the bliss model), supplementary tables (4e,f) and relevant sections in the main text and methods.

6. Discussion - Implications of Findings:
- While the discussion on the effects of sweeteners is well articulated, it often feels speculative. Including recent longitudinal studies demonstrating these health outcomes will support claims (for example, linking sweetener consumption to obesity and cardiovascular diseases). Additionally, balancing health benefits against potential negative outcomes could provide a nuanced understanding.

-> Thank you for the comment, we have revised the discussion.

"Multiple studies have examined the effects of long-term sweetener consumption across different stages of human life, reporting a broad range of impacts on both the host and gut microbiome (e.g. references[28,90–94]; cross-sectional studies and clinical trials reviewed in[95]). Some found significant links between sweetener consumption and obesity and cardiovascular disease[96–100]. Future investigations will be necessary to elucidate how concurrently ingested food components, pharmaceuticals, and other bioactive compounds modulate the impact of sweeteners on the gut microbiome and host physiology."

7. Conclusion - Future Directions: - The conclusion could more forcefully highlight the implications of the findings regarding the human microbiome and potential therapeutic uses of duloxetine and isosteviol. Suggesting specific mechanisms for further exploration (potentially based on differential protein abundance techniques noted in your results) could pave the way for future research directions.

-> Thank you for the comment. We have thoroughly revised the Discussion section. Although, based on our results for single strains and *in vitro* communities, we would like to refrain from implying any direct health implications or comment on use of medications. Yet, our study warrants further investigation in in vivo settings. We have therefore added the following to the discussion:

"This study also highlights important directions for future research with potential public health relevance. Given the substantial inter-individual variability in gut microbiota composition at the strain level, it will be critical to determine whether the metabolic effects observed here are strain specific. Such insights could inform personalized approaches to assess health risks originating from compound co-consumption as well as for microbiome-targeted diets and therapies. Furthermore, comprehensive in vivo profiling of the concentrations, kinetics, and dynamics of relevant compounds is essential to bridge the gap between in vitro findings and real-world exposures in human populations. These efforts will be key to evaluating the broader risks of microbiome–compound combination interactions for population health and consumer safety. Mechanistic insights from our study provide a starting point to unravel compound combination effects in the complex microbiome context."

8. References Assessment and Use:
- The references listed support many of the central concepts. However, expanding the discussion around studies indicating the long-term effects of sweeteners on gut health would provide depth, particularly highlighting earlier studies that discuss how changes in bacterial metabolites might mediate host interactions.

-> Thank you for the comment. We have revised the discussion accordingly and added the following section addressing the long-term consumption of sweeteners. However, given that our study spans a limited timeframe—24 hours for single-species experiments and five passages (referring to five days) for *in vitro* communities—we do not consider it appropriate to speculate on long-term effects.

"Multiple studies have examined the effects of long-term sweetener consumption across different stages of human life, reporting a broad range of impacts on both the host and gut microbiome (e.g. references[28,90–94]; cross-sectional studies and clinical trials reviewed in[95]). Some found significant links between sweetener consumption and obesity and cardiovascular disease[96–100]. Future investigations will be necessary to elucidate how concurrently ingested food components, pharmaceuticals, and other bioactive compounds modulate the impact of sweeteners on the gut microbiome and host physiology."

And in the Results:

"In the context of artificial sweeteners, longitudinal studies have shown altered serum levels of gut microbial metabolites[27]."

9. Clarifying Limitations:
- I noticed there wasn't much discussion pertaining to potential limitations of the study. It would be prudent to acknowledge confounding factors, such as dietary variances that could skew results. This transparency will help refine future studies and reinforce the validity of your current findings.

->

We have revised the discussion as outlined in the response to comment #7.

10. Biological Context of Cytokines:
- The discussion on IL-6 and IL-8 should be deepened concerning their roles in neutrophil biology. Elucidating the dual nature of their pro- and anti-inflammatory roles will give readers a more robust understanding of how sweeteners might modulate immune responses within the gut context.

-> We have now expanded the discussion on IL–6 and IL-8 production and the potential impact of their reduction induced by duloxetine and isosteviol on the gut homeostasis.

"Given the complexity and dynamic interplay between the host's inflammatory response and the gut microbiota, predicting the effects of the discussed changes is challenging. Nonetheless, since IL-6 and IL-8 play dual roles by exhibiting both pro- and anti-inflammatory effects depending on the context, the consequences of their modulation for host immunity will depend on the individual's health status."

18th Dec 2025

Manuscript Number: MSB-2025-13043R
Title: Impact of low-calorie sweeteners on gut bacteria is modulated by common xenobiotics

Dear Prof Patil,

Thank you again for submitting your revised work to Molecular Systems Biology. We have now heard back from two of the original three reviewers who evaluated your study; unfortunately Reviewer 2 did not re-review and therefore we also involved a new Reviewer 4 to look over the previous comments and your responses. As you will see below, the Reviewer 3 is fully supportive of the paper, while Reviewers 1 and 4 have significant remaining overlapping concerns on the interpretability of the results, which they agreed upon in a cross-commenting session would need to be addressed in full with additional experiments and/or analyses. We would therefore ask you to address their concerns in a revision. In particular it will be necessary to compare/justify physiological duloxetine concentrations in the gut compared to that used in your assay in vitro, address concerns on the unstable DMSO control, and bolster the analysis of single/synthetic mix of isolates with some data from human gut microbial samples (which could potentially be done with publicly available data on sweetener consumption). All other comments would need to be addressed in full. Please let me know in case you would like to discuss in further detail any of the any of the reviewer comments, I would be happy to schedule a call.

We remind you that we have the following formatting requirements:

1) A .docx formatted version of the manuscript text (including legends for main figures, EV figures and tables). Please make sure that the changes are highlighted to be clearly visible. Alternatively you may choose to submit your manuscript as a LaTeX file.

2) Individual production quality figure files as .eps, .tif, .jpg (one file per figure). For guidance, download the 'Figure Guide PDF' (https://media.springernature.com/original/springer-cms/rest/v1/content/27825798/data/v1).

3) At EMBO Press we ask authors to provide source data for the main figures. Our source data coordinator will contact you to discuss which figure panels we would need source data for and will also provide you with helpful tips on how to upload and organize the files.

4) A .docx formatted letter INCLUDING the reviewers' reports and your detailed point-by-point responses to their comments. As part of the EMBO Press transparent editorial process, the point-by-point response is part of the Peer Review File (PRF), which will be published alongside your paper.

5) A complete author checklist, which you can download from our author guidelines (https://link.springer.com/journal/44320/submission-guidelines#cms). Please insert information in the checklist that is also reflected in the manuscript. The completed author checklist will also be part of the PRF.

6) Please note that all corresponding authors are required to supply an ORCID ID for their name upon submission of a revised manuscript.

7) It is mandatory to include a 'Data Availability' section after the Materials and Methods. Before submitting your revision, primary datasets produced in this study need to be deposited in an appropriate public database, and the accession numbers and database listed under 'Data Availability'. Please remember to provide a reviewer password if the datasets are not yet public (see https://link.springer.com/partners/embo-press/editorial-policies#Data%20availability%20statement).

In case you have no data that requires deposition in a public database, please state so in this section as follows: "This study includes no data deposited in external repositories". Note that the Data Availability Section is restricted to new primary data that are part of this study.

8) All Materials and Methods need to be described in the main text using our 'Structured Methods' format, which is required for all research articles. According to this format, the Methods section includes a Reagents and Tools Table (listing key reagents, experimental models, software and relevant equipment and including their sources and relevant identifiers) followed by a Methods and Protocols section describing the methods using a step-by-step protocol format. The aim is to facilitate adoption of the methodologies across labs. Please upload the Reagents and Tools table as a separate document when submitting your revised manuscript. More information on how to adhere to this format as well as a downloadable template (.docx) for the Reagents and Tools Table can be found in our author guidelines: https://link.springer.com/journal/44320/submission-guidelines#cms

9) For data quantification: please specify the name of the statistical test used to generate error bars and p-values, the number (n) of independent experiments (specify technical or biological replicates) underlying each data point and the test used to

calculate p-values in each figure legend. The figure legends should contain a basic description of n, p-values and the test applied. Graphs must include a description of the bars and the error bars (s.d., s.e.m.). Please provide exact p-values (in either the figure or figure legend).

10) Our journal encourages inclusion of *data citations in the reference list* to directly cite datasets that were re-used and obtained from public databases. Data citations in the article text are distinct from normal bibliographical citations and should directly link to the database records from which the data can be accessed. In the main text, data citations are formatted as follows: "Data ref: Smith et al, 2001" or "Data ref: NCBI Sequence Read Archive PRJNA342805, 2017". In the Reference list, data citations must be labeled with "[DATASET]". A data reference must provide the database name, accession number/identifiers and a resolvable link to the landing page from which the data can be accessed at the end of the reference. Further instructions are available at .

11) We replaced Supplementary Information with Expanded View (EV) Figures and Tables that are collapsible/expandable online. EV Figures should be cited as 'Figure EV1, Figure EV2" etc... in the text and their respective legends should be included in the main text after the legends of regular figures.
- For the figures that you do NOT wish to display as Expanded View figures, they should be bundled together with their legends in a single PDF file called *Appendix*, which should start with a short Table of Content. Appendix figures should be referred to in the main text as: "Appendix Figure S1, Appendix Figure S2" etc.
- Additional Tables/Datasets should be labeled and referred to as Table EV1, Dataset EV1, etc. Legends should be provided in a separate tab in case of .xls files. Alternatively, the legend can be supplied as a separate text file (README) and zipped together with the Table/Dataset file.
See detailed instructions here:
https://link.springer.com/journal/44320/submission-guidelines#cms

12) Author contributions: CRediT has replaced the traditional author contributions section because it offers a systematic machine-readable author contributions format that allows for more effective research assessment. Please remove the Authors Contributions from the manuscript and use the free text boxes beneath each contributing author's name in our system to add specific details on the author's contribution. More information is available in our guide to authors.

13) Disclosure statement and competing interests: We updated our journal's competing interests policy in January 2022 and request authors to consider both actual and perceived competing interests. Please review the policy https://link.springer.com/partners/embo-press/editorial-policies#Competing%20interest%20disclosures and update your competing interests if necessary.

14) Every published paper now includes a 'Synopsis' to further enhance discoverability. Synopses are displayed on the journal webpage and are freely accessible to all readers. They include a short stand first (maximum of 300 characters, including space) as well as 2-5 one-sentences bullet points that summarizes the paper. Please write the bullet points to summarize the key NEW findings. They should be designed to be complementary to the abstract - i.e. not repeat the same text. We encourage inclusion of key acronyms and quantitative information (maximum of 30 words / bullet point). Please use the passive voice. Please attach these in a separate file or send them by email, we will incorporate them accordingly.

Please also suggest a striking image or visual abstract to illustrate your article as a PNG file 550 px wide x 300-600 px high.

Please note that these would be the final versions and changes during proofing are usually not allowed.

15) As part of the EMBO Publications transparent editorial process initiative (see our policy here: https://link.springer.com/partners/embo-press/editorial-policies#Peer%20review), Molecular Systems Biology will publish online a Peer Review File (PRF) to accompany accepted manuscripts.
In the event of acceptance, this file will be published in conjunction with your paper and will include the anonymous referee reports, your point-by-point response and all pertinent correspondence relating to the manuscript. Let us know whether you agree with the publication of the PRF and as here, if you want to remove or not any figures from it prior to publication.
Please note that the Author checklist will be published at the end of the PRF.

Molecular Systems Biology has a "scooping protection" policy, whereby similar findings that are published by others during review or revision are not a criterion for rejection. Should you decide to submit a revised version, I do ask that you get in touch after three months if you have not completed it, to update us on the status.

Yours sincerely,

Poonam Bheda, PhD
Scientific Editor
Molecular Systems Biology

Reviewer #1:

The authors have addressed my previous concerns; however, after reading the revised manuscript and clarifications, I still have reservations.

In Figure 2C, the number of species in the DMSO control declines across passages, with a sharper drop by P5. This indicates the system remains in flux and has not reached a stable baseline. Comparing treatments against an unstable control reduces confidence that the observed differences are representative. It also makes Figure 2F problematic: interpreting relative abundances across treatments is confounded when overall richness in the DMSO control has fallen so markedly.

In an adult gut microbiome, Parabacteroides merdae typically constitutes <2% of total taxa. Duloxetine plus metabolites in the gut lumen is often cited around ~50 µM, but the parent duloxetine concentration is likely much lower because most biotransformation occurs in the liver prior to biliary secretion. Moreover, bacterial densities in stool differ from those in the authors' assay conditions. Could the authors clarify whether the bacteria:duloxetine ratios (cell density and molarity) used in their assays fall within physiologically relevant ranges for the human gut?

Finally, while the assay is suitable for testing compound effects on single isolates, it is not evident that these results translate to a complex community. For example, P. merdae is a recognised cross-feeder that benefits from sugars liberated by primary degraders and, in turn, releases succinate and acetate that support other taxa. Please discuss how such trophic interactions could modulate-or even reverse-the single-isolate effects reported here, and consider whether community-level experiments or co-cultures are needed to substantiate the conclusions. That needs be part of the discussion on limitation as the authors have no evidence that this translate to gut microbiome.

Reviewer #3:

The authors have adequtely addressed my comments, and it is now suitable for publication consideration.

Reviewer #4:

In their manuscript, the authors systematically screened interactions between non-caloric sweeteners, selected xenobiotics, and a 25-strain synthetic gut microbial community to assess effects on bacterial growth, community composition, metabolite secretion, and host-relevant readouts in vitro.
While the study provides a comprehensive mapping of sweetener-xenobiotic-microbe interactions, several limitations constrain its biological interpretation:
1-The use of a defined 25-strain synthetic community, rather than starting with human fecal microbiota, limits ecological relevance and may miss emergent interactions present in more complex communities.
2-The link between observed suppression of certain beneficial bacteria and SCFA production remains indirect: although decreases in butyrate and propionate are reported, the authors do not explicitly connect these metabolites to the specific SCFA-producing strains that were suppressed.
3-The choice of Parabacteroides merdae and Roseburia intestinalis for proteomic analysis appears to be based on experimental tractability and general metabolic relevance, rather than a systematic rationale tied to SCFA production or community-level effects, leaving a gap in mechanistic interpretation.
4-The study does not contextualize findings within open-access human metagenomic datasets that include sweetener consumption, limiting insights into population-level relevance.
5-Several tested sweeteners and drugs, such as duloxetine, are mostly absorbed in the small intestine and are unlikely to reach the colon at concentrations comparable to those used in vitro. Conversely, compounds like steviol glycosides or sucralose are poorly absorbed and may reach colonic microbes, but their in vivo concentrations and residence times are highly variable. By applying a single concentration of all compounds in a rich medium, the study overlooks key aspects of human gastrointestinal pharmacokinetics, including differential absorption, microbial metabolism, and exposure duration, which are critical for interpreting microbial and metabolite responses in a physiologically meaningful context.

18th Dec 2025

Manuscript Number: MSB-2025-13043R
Title: Impact of low-calorie sweeteners on gut bacteria is modulated by common xenobiotics

Dear Prof Patil,

Thank you again for submitting your revised work to Molecular Systems Biology. We have now heard back from two of the original three reviewers who evaluated your study; unfortunately Reviewer 2 did not re-review and therefore we also involved a new Reviewer 4 to look over the previous comments and your responses. As you will see below, the Reviewer 3 is fully supportive of the paper, while Reviewers 1 and 4 have significant remaining overlapping concerns on the interpretability of the results, which they agreed upon in a cross-commenting session would need to be addressed in full with additional experiments and/or analyses. We would therefore ask you to address their concerns in a revision. In particular it will be necessary to compare/justify physiological duloxetine concentrations in the gut compared to that used in your assay in vitro, address concerns on the unstable DMSO control, and bolster the analysis of single/synthetic mix of isolates with some data from human gut microbial samples (which could potentially be done with publicly available data on sweetener consumption). All other comments would need to be addressed in full. Please let me know in case you would like to discuss in further detail any of the any of the reviewer comments, I would be happy to schedule a call.

→ Thank you for handling our manuscript and your constructive feedback. Below we provide point-by-point response to all reviewers' comments, including human relevance of the drug concentration used.

The main comments relate to showing 'human relevance' of our data. However, this is beyond the scope of the current study and to our understanding is not required for Molecular Systems Biology. The strength of our study is in providing a system-level view on the impact of sweeteners on gut bacteria and their combination with other xenobiotics, and we hope that this is recognised. This view is both complementary and necessary to go beyond the correlative approach of cohort studies.

We have edited the manuscript to mention the limitations of our approach.

We remind you that we have the following formatting requirements:

1) A .docx formatted version of the manuscript text (including legends for main figures, EV figures and tables). Please make sure that the changes are highlighted to be clearly visible. Alternatively you may choose to submit your manuscript as a LaTeX file.

2) Individual production quality figure files as .eps, .tif, .jpg (one file per figure). For guidance, download the 'Figure Guide PDF' (https://media.springernature.com/original/springer-cms/rest/v1/content/27825798/data/v1).

3) At EMBO Press we ask authors to provide source data for the main figures. Our source data coordinator will contact you to discuss which figure panels we would need source data for and will also provide you with helpful tips on how to upload and organize the files.

4) A .docx formatted letter INCLUDING the reviewers' reports and your detailed point-by-point responses to their comments. As part of the EMBO Press transparent editorial process, the point-by-point response is part of the Peer Review File (PRF), which will be published alongside your paper.

5) A complete author checklist, which you can download from our author guidelines (https://link.springer.com/journal/44320/submission-guidelines#cms). Please insert information in the checklist that is also reflected in the manuscript. The completed author checklist will also be part of the PRF.

6) Please note that all corresponding authors are required to supply an ORCID ID for their name upon submission of a revised manuscript.

7) It is mandatory to include a 'Data Availability' section after the Materials and Methods. Before submitting your revision, primary datasets produced in this study need to be deposited in an appropriate public database, and the accession numbers and database listed under 'Data Availability'. Please remember to provide a reviewer password if the datasets are not yet public (seehttps://link.springer.com/partners/embo-press/editorial-policies#Data%20availability%20statement).

In case you have no data that requires deposition in a public database, please state so in this section as follows: "This study includes no data deposited in external repositories". Note that the Data Availability Section is restricted to new primary data that are part of this study.

8) All Materials and Methods need to be described in the main text using our 'Structured Methods' format, which is required for all research articles. According to this format, the Methods section includes a Reagents and Tools Table (listing key reagents, experimental models, software and relevant equipment and including their sources and relevant identifiers) followed by a Methods and Protocols section describing the methods using a step-by-step protocol format. The aim is to facilitate adoption of the methodologies across labs. Please upload the Reagents and Tools table as a separate document when submitting your revised manuscript. More information on how to adhere to this format as well as a downloadable template (.docx) for the Reagents and Tools Table can be found in our author guidelines: https://link.springer.com/journal/44320/submission-guidelines#cms

9) For data quantification: please specify the name of the statistical test used to generate error bars and p-values, the number (n) of independent experiments (specify technical or biological replicates) underlying each data point and the test used to calculate p-values in each figure legend. The figure legends should contain a basic description of n, p-values and the test applied. Graphs must include a description of the bars and the error bars (s.d., s.e.m.). Please provide exact p-values (in either the figure or figure legend).

10) Our journal encourages inclusion of *data citations in the reference list* to directly cite datasets that were re-used and obtained from public databases. Data citations in the article text are distinct from normal bibliographical citations and should directly link to the database records from which the data can be accessed. In the main text, data citations are formatted as follows: "Data ref: Smith et al, 2001" or "Data ref: NCBI Sequence Read Archive PRJNA342805, 2017". In the Reference list, data citations must be labeled with "[DATASET]". A data reference must provide the database name, accession number/identifiers and a resolvable link to the landing page from which the data can be accessed at the end of the reference. Further instructions are available athttps://link.springer.com/journal/44320/submission-guidelines#cms-Reference-guidelines.

11) We replaced Supplementary Information with Expanded View (EV) Figures and Tables that are collapsible/expandable online. EV Figures should be cited as 'Figure EV1, Figure EV2" etc... in the text and their respective legends should be included in the main text after the legends of regular figures.
- For the figures that you do NOT wish to display as Expanded View figures, they should be bundled together with their legends in a single PDF file called *Appendix*, which should start with a short Table

of Content. Appendix figures should be referred to in the main text as: "Appendix Figure S1, Appendix Figure S2" etc.
- Additional Tables/Datasets should be labeled and referred to as Table EV1, Dataset EV1, etc. Legends should be provided in a separate tab in case of .xls files. Alternatively, the legend can be supplied as a separate text file (README) and zipped together with the Table/Dataset file.
See detailed instructions here:
https://link.springer.com/journal/44320/submission-guidelines#cms

12) Author contributions: CRediT has replaced the traditional author contributions section because it offers a systematic machine-readable author contributions format that allows for more effective research assessment. Please remove the Authors Contributions from the manuscript and use the free text boxes beneath each contributing author's name in our system to add specific details on the author's contribution. More information is available in our guide to authors.

13) Disclosure statement and competing interests: We updated our journal's competing interests policy in January 2022 and request authors to consider both actual and perceived competing interests. Please review the policy https://link.springer.com/partners/embo-press/editorial-policies#Competing%20interest%20disclosures and update your competing interests if necessary.

14) Every published paper now includes a 'Synopsis' to further enhance discoverability. Synopses are displayed on the journal webpage and are freely accessible to all readers. They include a short stand first (maximum of 300 characters, including space) as well as 2-5 one-sentences bullet points that summarizes the paper. Please write the bullet points to summarize the key NEW findings. They should be designed to be complementary to the abstract - i.e. not repeat the same text. We encourage inclusion of key acronyms and quantitative information (maximum of 30 words / bullet point). Please use the passive voice. Please attach these in a separate file or send them by email, we will incorporate them accordingly.

Please also suggest a striking image or visual abstract to illustrate your article as a PNG file 550 px wide x 300-600 px high.

Please note that these would be the final versions and changes during proofing are usually not allowed.

15) As part of the EMBO Publications transparent editorial process initiative (see our policy here:https://link.springer.com/partners/embo-press/editorial-policies#Peer%20review), Molecular Systems Biology will publish online a Peer Review File (PRF) to accompany accepted manuscripts. In the event of acceptance, this file will be published in conjunction with your paper and will include the anonymous referee reports, your point-by-point response and all pertinent correspondence relating to the manuscript. Let us know whether you agree with the publication of the PRF and as here, if you want to remove or not any figures from it prior to publication.
Please note that the Author checklist will be published at the end of the PRF.

Molecular Systems Biology has a "scooping protection" policy, whereby similar findings that are published by others during review or revision are not a criterion for rejection. Should you decide to submit a revised version, I do ask that you get in touch after three months if you have not completed it, to update us on the status.

Yours sincerely,

Poonam Bheda, PhD

Scientific Editor
Molecular Systems Biology

Please find our responses below in blue.

Reviewer #1:
The authors have addressed my previous concerns; however, after reading the revised manuscript and clarifications, I still have reservations.

In Figure 2C, the number of species in the DMSO control declines across passages, with a sharper drop by P5. This indicates the system remains in flux and has not reached a stable baseline. Comparing treatments against an unstable control reduces confidence that the observed differences are representative. It also makes Figure 2F problematic: interpreting relative abundances across treatments is confounded when overall richness in the DMSO control has fallen so markedly.

→ We thank the reviewer for this remark. To address this, we quantitatively assessed community stability across passages using variability across vectors of relative abundances (FST-based Assessment of Variability across vectors of relative Abundances, FAVA), a tool developed to evaluate temporal stability of microbial communities (Morrison et al. *PNAS* 2025; PMID: 40063791). FAVA assigns F_{ST} values ranging from 0 (identical communities) to 1 (maximally dissimilar). In the DMSO control, the highest F_{ST} values were observed immediately after assembly, consistent with expected post-inoculation dynamics. Importantly, F_{ST} values decreased and plateaued by passage 3, indicating convergence to a stable community state. Moreover, treated and untreated (DMSO) communities were compared within the same passage number, ensuring that treatment effects were evaluated against passage-matched controls, thereby supporting the validity of the comparative analyses.

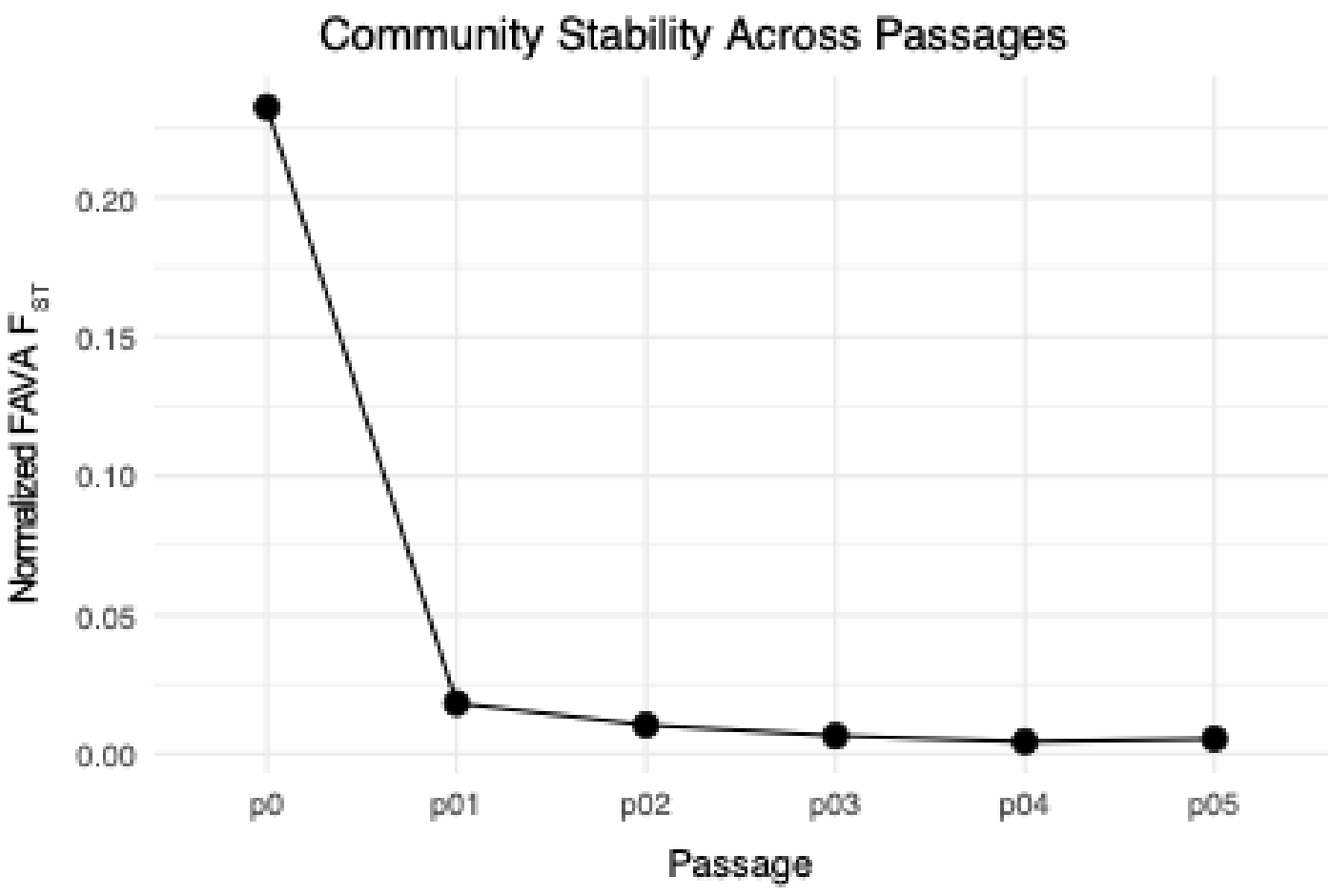

Variability across passages of the 25-species sweetener community was highest immediately after inoculation (FAVA = 0.233) and subsequently decreased, reaching and remaining at values below 0.019.

In addition, we would like to note that the proteomics and metabolomics analyses on single species are independently performed and thus the results, which clearly show molecular effects at system level, are self-standing.

Action taken:

We have added the FAVA analysis in the Appendix (Figure S5).

In an adult gut microbiome, Parabacteroides merdae typically constitutes <2% of total taxa. Duloxetine plus metabolites in the gut lumen is often cited around ~50 μM, but the parent duloxetine concentration is likely much lower because most biotransformation occurs in the liver prior to biliary secretion. Moreover, bacterial densities in stool differ from those in the authors' assay conditions. Could the authors clarify whether the bacteria:duloxetine ratios (cell density and molarity) used in their assays fall within physiologically relevant ranges for the human gut?

→ We thank the reviewer for this comment. The duloxetine concentration used in our experiments (50 μM) was chosen to approximate reported levels (PMID: 40462285 and PMID: 12920170) and to match those used in previous studies (PMID: 34497420).

Regarding Lantz et al 2003 (PMID: 12920170), we agree with the reviewer that approximately 20% of the administered dose was recovered in faeces. Notably, up to 4.1% of the total administered dose was recovered in human faeces as unchanged duloxetine (Table 5 in Lantz et al.). It is also important to note that this study evaluated a single 20.2 mg oral dose, which is below the established therapeutic dose range of duloxetine (typically 30–60 mg/day, with doses up to 120 mg/day, see https://www.medicalnewstoday.com/articles/drugs-cymbalta-dosage#3 and https://www.nhs.uk/medicines/duloxetine/how-and-when-to-take-duloxetine/). Such higher therapeutic doses may further alter duloxetine absorption and metabolism. In the absence of longitudinal faecal excretion data from patients receiving therapeutic dosing, we selected a dose consistent with those used in prior studies to ensure comparability.

Nevertheless, we do not claim any *in vivo* relevance at this stage. The strength of our study is in providing mechanistic insights and leads that can be used to generate hypotheses for *in vivo* studies (or interpretation of results from these). For example, our previous *in vitro* studies on testing effect of therapeutic drugs in bacteria (Maier et al. Nature 2018; Klünemann et al. Nature 2021; Garcia-Santamarina et al. Cell 2024) have contributed to the acknowledgement of the impact of non-antibiotics on human gut microbiota (which has been now shown *in vivo* by multiple independent studies). Drugs are now frequently identified as major contributors (sometimes even above diet) to microbiome variation. Thus, *in vitro* studies like ours are important to move from correlations to mechanisms.

Finally, while the assay is suitable for testing compound effects on single isolates, it is not evident that these results translate to a complex community. For example, P. merdae is a recognised cross-feeder that benefits from sugars liberated by primary degraders and, in turn, releases succinate and acetate that support other taxa. Please discuss how such trophic interactions could modulate-or even reverse-the single-isolate effects reported here, and consider whether community-level experiments or co-cultures are needed to substantiate the conclusions. That needs be part of the discussion on limitation as the authors have no evidence that this translate to gut microbiome.

We agree that trophic interactions, including cross-feeding, can substantially modulate species responses in complex communities (not only in the case of *P. merdae*), and effects are expected to differ from those observed in monocultures. This consideration motivated our follow-up experiments in the defined 25-species community, in which we tested co-exposure to duloxetine and isosteviol. These experiments were not intended to replicate *in vivo* gut conditions or resemble faecal communities; rather, they were designed to monitor species-level dynamics in a controlled community where changes can be monitored and compared with monocultures. Such a synthetic community approach is crucial in identifying mechanisms that are difficult to discern *in vivo*. For example, our previous study showed that around 60% of monoculture effects hold also in the community-context

(PMID: 39321801).

Action:
We have altered the following section of the discussion to point out limitations more clearly:

"Both of our experimental screens—one examining bacterium–sweetener interactions and the other assessing interactions among bacteria, sweeteners, and co-consumed compounds—were performed at a single concentration (50 µM for both sweeteners and co-consumed compounds) in a single growth medium (mGAM), using four co-consumed compounds. This controlled design allowed systematic interrogation of thousands of interactions but captures only a limited portion of the broader spectrum of dietary, pharmaceutical, and environmental exposures, as well as the considerable inter-individual variability in gut microbiota composition. Moreover, the choice of growth medium can influence microbial growth dynamics and interaction outcomes, potentially limiting the generalizability and sensitivity of in vitro findings[21]. The species composition of the synthetic community further restricts interactions to those among the included taxa. Trophic interactions, such as cross-feeding, can modify species responses in communities relative to monocultures. Despite these limitations, synthetic communities provide mechanistic insights that are challenging to obtain in vivo, and previous work suggests that approximately 60% of monoculture effects are preserved in community contexts[101]."

Reviewer #3:

The authors have adequately addressed my comments, and it is now suitable for publication consideration.

We thank the reviewer for their careful evaluation and constructive feedback.

Reviewer #4:

In their manuscript, the authors systematically screened interactions between non-caloric sweeteners, selected xenobiotics, and a 25-strain synthetic gut microbial community to assess effects on bacterial growth, community composition, metabolite secretion, and host-relevant readouts in vitro.
While the study provides a comprehensive mapping of sweetener-xenobiotic-microbe interactions, several limitations constrain its biological interpretation:
1-The use of a defined 25-strain synthetic community, rather than starting with human fecal microbiota, limits ecological relevance and may miss emergent interactions present in more complex communities

→ The strength of our study is in providing a system-level view on the impact of sweeteners on gut bacteria and their combination with other xenobiotics, and we hope that it is recognised. This view is both complementary and necessary to go beyond the correlative approach of cohort studies. Our experiments were not intended to replicate *in vivo* gut conditions or resemble faecal communities; rather, they were designed to monitor species-level dynamics in a controlled community where changes can be better quantified. Our single species and synthetic community approach is crucial in identifying mechanisms that are difficult to discern in vivo or by using complex undefined communities.

For example, our previous *in vitro* studies on testing effect of therapeutic drugs in bacteria (Maier et al. Nature 2018; Klünemann et al. Nature 2021; Garcia-Santamarina et al. Cell 2024) have contributed to the acknowledgement of the impact of non-antibiotics on human gut microbiota (which has been now shown *in vivo* by multiple independent studies). Drugs are now frequently identified as

major contributors (sometimes even above diet) to microbiome variation. Thus, *in vitro* studies like ours are important to move from correlations to mechanisms.

2-The link between observed suppression of certain beneficial bacteria and SCFA production remains indirect: although decreases in butyrate and propionate are reported, the authors do not explicitly connect these metabolites to the specific SCFA-producing strains that were suppressed.

We agree with the reviewer that the observed changes in SCFA concentrations may not be directly attributed to the suppression of individual SCFA-producing species. This is primarily due to the complexity of SCFA metabolism within microbial communities. First, multiple bacterial species are capable of producing and biotransforming SCFAs. Second, SCFA biosynthesis is typically a collective process, with many species contributing to the overall production of these metabolites (PMID: 31123355; 38565643). Third, SCFA metabolism in the gut is strongly shaped by cross-species interactions, including metabolic cross-feeding and functional redundancy, which can decouple changes in metabolite levels from the abundance of specific taxa (BioRxiv, doi: 10.1101/2025.05.14.653412). Consequently, directly linking altered SCFA levels to the suppression of individual strains is challenging and beyond the scope of the current study (which already contains extensive novel data).

3-The choice of Parabacteroides merdae and Roseburia intestinalis for proteomic analysis appears to be based on experimental tractability and general metabolic relevance, rather than a systematic rationale tied to SCFA production or community-level effects, leaving a gap in mechanistic interpretation.

In monoculture, *Roseburia intestinalis* exhibited the strongest response to co-exposure (Fig. 1d), while *Parabacteroides merdae* showed a statistically significant response that did not meet our predefined 20% effect-size threshold (Appendix Fig. S7). Yet, within the synthetic community, *Parabacteroides merdae* displayed a significant and sustained decline across all five passages under co-exposure conditions, reinforcing its relevance at the community level (Fig. 2f). Together, these patterns made *P. merdae* and *R. intestinalis* logical candidates for deeper mechanistic investigation. Additionally, the availability of a transposon mutant library for *P. merdae* enabled forward genetic analyses, further supporting its selection for follow-up experiments. We do not think that choosing a species that is experimentally tractable is a negative feature – on the contrary, this is the basis of modern molecular biology and systems biology approach.

4-The study does not contextualize findings within open-access human metagenomic datasets that include sweetener consumption, limiting insights into population-level relevance.

Connecting mechanistic study with cohort studies is a complex and time-consuming task and beyond the scope of this work.

5-Several tested sweeteners and drugs, such as duloxetine, are mostly absorbed in the small intestine and are unlikely to reach the colon at concentrations comparable to those used in vitro. Conversely, compounds like steviol glycosides or sucralose are poorly absorbed and may reach colonic microbes, but their in vivo concentrations and residence times are highly variable. By applying a single concentration of all compounds in a rich medium, the study overlooks key aspects of human gastrointestinal pharmacokinetics, including differential absorption, microbial metabolism, and exposure duration, which are critical for interpreting microbial and metabolite responses in a physiologically meaningful context.

We thank the reviewer for raising this important point regarding gastrointestinal pharmacokinetics and colonic exposure. Indeed, the fraction of ingested sweeteners and drugs that reaches the colon varies substantially depending on compound-specific absorption and metabolism. For example, less than 1% of ingested aspartame and acesulfame K is estimated to reach the colon, whereas advantame, saccharin, sucralose, steviol glycosides, and polyols such as maltitol and lactitol can reach the large intestine in varying but potentially relevant amounts (reviewed in PMID: 32326137). Notably, even compounds that are largely absorbed or metabolized prior to colonic entry, such as aspartame, have been reported to exert microbiota-mediated effects in vivo, including aggravation of colitis in a mouse model (PMID: 1894884).

To contextualize the in vitro exposures used in this study, the median concentration of sweeteners in the human colon has been estimated at approximately 49.6 µM (Supplementary Table 3b). Nevertheless, we acknowledge that applying a single concentration across compounds does not capture the full complexity of human gastrointestinal pharmacokinetics, including differential absorption, residence time, and microbial metabolism. A uniform concentration was therefore chosen to enable systematic and controlled comparisons across compounds and to identify relative microbial and metabolic responses under standardized conditions. We view this approach as a reductionist but necessary first step toward dissecting compound-specific and combinatorial effects, which can be further refined in future studies using dynamic dosing or physiologically informed exposure models.

31st Mar 2026
RE: Manuscript MSB-2025-13043RR, Impact of low-calorie sweeteners on gut bacteria is modulated by common xenobiotics

Dear Prof Patil,

Thank you again for submitting your work to Molecular Systems Biology. We have now heard back from the two referees whom we asked to re-evaluate your manuscript. As you will see from the reports below, although Reviewer 4 found their concerns to be addressed, Reviewer 1 maintained their concerns on the unstable DMSO-treated cultures and whether conclusions can be robustly drawn. Given this potential technical flaw, we contacted Reviewers 2, 3, and 4 for their opinion on whether they agreed that this was a major concern. Unfortunately Reviewer 4 agrees with Reviewer 1 that the unstable control community and culture conditions is a substantial issue that makes the robustness and value of the work questionable. Given this advice, I am afraid we see little choice but to return the manuscript to you at this point with the decision that we cannot offer to publish it.

I am sorry that the review of your work did not result in a more favourable outcome on this occasion, but I hope that you will not be discouraged from sending your work to Molecular Systems Biology in the future.

Thank you for the opportunity to examine this work.

Yours sincerely,

Poonam Bheda, PhD
Scientific Editor
Molecular Systems Biology

Reviewer #1:

I remain unconvinced by the conclusions, even when considering the FAVA-based analyses. In particular, the DMSO control shows a loss of fewer species between P0 and P1 than between P4 and P5, where three species are lost. This decline suggests a deterioration of culture stability and likely reflects suboptimal culture conditions rather than a controlled or stable system.

In contrast, I have seen recent unpublished results where human-derived microbiomes were passaged for 15 passages and the community structure was maintained with substantially greater stability.

The authors state that their objective is to investigate species-level dynamics within a controlled microbial community, where changes can be more precisely quantified. However, the manuscript repeatedly refers to the "gut" including in the title, which is misleading. The community used here is composed largely of strains obtained from culture collections (e.g. DSMZ), originally isolated from stool samples, often many years ago. Such a reconstructed community does not capture the ecological complexity, functional redundancy, or host-associated context of an actual gut microbiome.

This distinction should be made explicit throughout the manuscript. At present, the terminology risks overstating the physiological relevance of the model system and may lead readers to overinterpret the findings in the context of in vivo gut microbiome dynamics.

Reviewer #4:

The concerns have been adequately addressed

** As a service to authors, EMBO Press offers the possibility to directly transfer declined manuscripts to another EMBO Press title or to the open access journal Life Science Alliance launched in partnership between EMBO Press, Rockefeller University Press and Cold Spring Harbor Laboratory Press. The full manuscript and if applicable, reviewers' reports, are automatically sent

to the receiving journal to allow for fast handling and a prompt decision on your manuscript. For more details of this service, and to transfer your manuscript please click on Link Not Available. **

Reviewer #1:

I remain unconvinced by the conclusions, even when considering the FAVA-based analyses. In particular, the DMSO control shows a loss of fewer species between P0 and P1 than between P4 and P5, where three species are lost. This decline suggests a deterioration of culture stability and likely reflects suboptimal culture conditions rather than a controlled or stable system.
In contrast, I have seen recent unpublished results where human-derived microbiomes were passaged for 15 passages and the community structure was maintained with substantially greater stability.

→
We respectfully disagree that the DMSO time course data implies suboptimal culturing conditions for three key reasons:

1. Variability in low abundance species is expected, and it is a common feature of any microbial community study and is not unique to our data. See the detailed response including additional analysis below. For this we also present a detailed comparison with an independent dataset from another study using a community assembly assay.
2. Reference to an unpublished data is not a valid argument – we have not seen the data. Nevertheless, in all likelihood, the data would also show the variability with regards to low abundance species as this is a well-known technical reality in microbiome analysis as detailed below.
3. The FAVA framework represents a rigorous mathematical approach derived from population genetics used for assessing in an unbiased way community variation. The reviewer has not presented any argument why the mathematical formalism in the FAVA method is not sufficient. If a reader would like to use another method, our data is publicly available.

Overall, an argument about low abundant species that do not matter to the core point that our paper makes (which is supported by detailed molecular analyses) should not hold out the publication – by that argument no microbiome paper would ever be published (especially those involving ex vivo communities or cohort studies where low abundance species/strains can never be accurately quantified using realistic sequencing depth).

Proposed action: a) We will alter the terminology from species loss to species being below detection limit to avoid any potential unclarity. **b)** We will include some of the additional analyses detailed below in the supplementary material.

<u>Detecting low abundance species in microbial communities:</u>

Species detection in amplicon sequencing analysis is greatly affected by sequencing depth. Species present at low abundance are particularly affected as they can be "lost" by spurious variation in sequencing output (which is a function of multiple factors including abundance levels, lysis efficiency of the species in question, DNA stability, stochasticity in PCR amplification etc.). Increasing sequencing depth would often uncover more species. If species abundance is oscillating around 0.1-0.01%, i.e. around 50 to 5 read out of a nominal 50k reads amplicon depth, species loss can simply be attributed to the limit of detection.

Of the 6 species not detected in our 16S amplicon analysis by T5, *A. muciniphilia* was already below our limit of detection after 2 transfers and fluctuating around 0.01% in all the replicates,

B. longum subs. infantis was not detected after the first passage. *L. gasserii* was found at 10 reads in one sample and 0 in others at the 5th passage, *O. splanchnicus* was not detected in only 1 sample while had < 15 reads in the other three. *P. distasonis* was below our limit of detection already after the second passage. Only *S. salivarius* was found below our limit of detection after 5 passages but oscillating around 0.02-0.09% at the 4th passage. Therefore only *S. salivarius* and *L. gasserii* effectively fell below our limit of detection at the 5th transfer and both representing species oscillating at around 0.1-0.01% at transfer 3 already.

The above is a documented effect in community stability analysis also for those derived *ex-vivo*. For instance, in the work Diaz et al (2022) - "Establishment and characterization of stable, diverse, fecal-derived *in vitro* microbial communities that model the intestinal microbiota" https://doi.org/10.1016/j.chom.2021.12.008 - the authors reported a decrease of amplicon sequence variants (ASVs), a proxy of taxonomy units, during their transfer experiments of the derived community as observed in our cases. They state: "*Thus, even though family-level composition is preserved for families conducive to culturing in these conditions, passaging leads to deterministic decreases to levels below the limit of detection, biased toward species that start at lower abundance*."

Our experiments simply recapitulate a similar phenomenon observed in an *ex-vivo* derived gut community and in community analysis of non-gut models (e.g. Goldford et al., 2018) supporting our point that we are approaching the limit of detection of rare species rather than a biological instability of our system (see Figure 1 and 2 below). The formalism used in the FAVA analysis does not give exaggerated weight to spurious species variation when their starting level is already low when the two transfers are compared to compute the F_{st} statistic. And thus, it is a valid way to formally assess stability.

Furthermore, Diaz et al. also report that "*Three technical replicates of SICs derived from one mouse contained 50 ASVs at >0.1% (a conservative measure of detectability), and all ASVs present in only one replicate had relative abundance <1%; higher variation in low-abundance ASVs is expected due to counting noise*". In our study, species that fail to be detected are one order of magnitude lower that the limit of detectability used by the cited work. Using their limit of detection would correspond to a variable read range of 19 to 170 in our dataset. To use a conservative threshold of 50 to 200 reads, species falling below our limit of detection would stabilise at passage 3 and passage 4 already. This is in line with their abundance being already very low and affected by "counting noise" as reported by the authors.

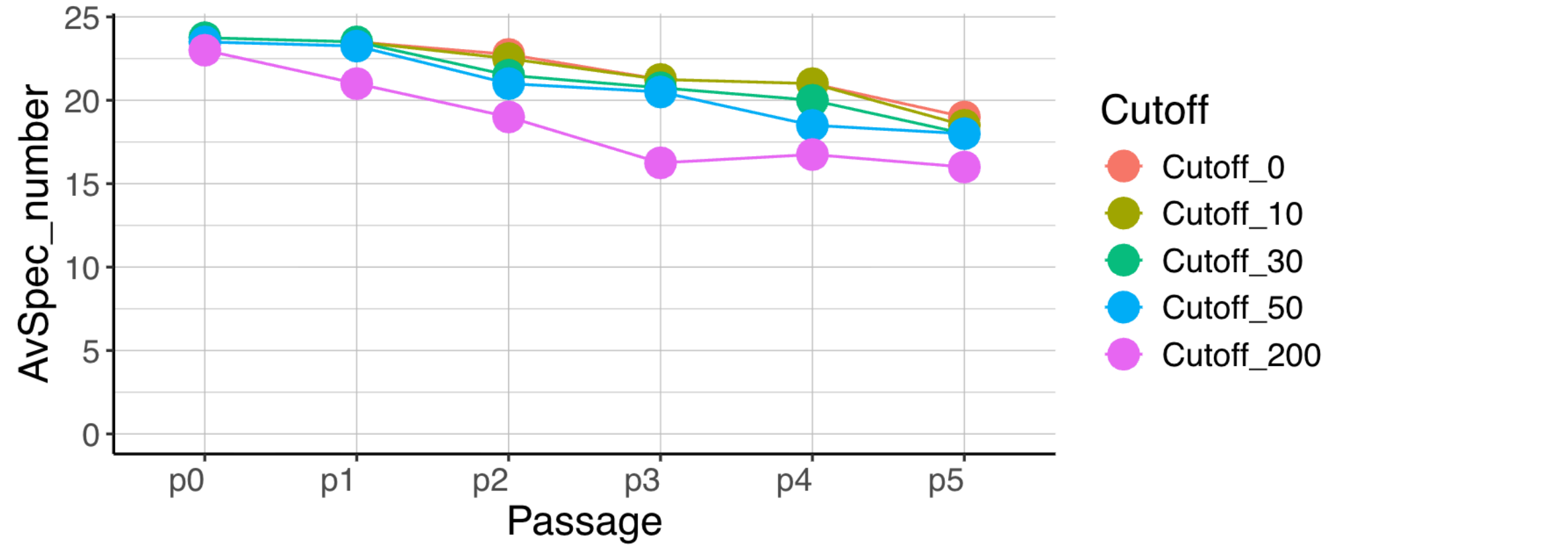

Figure 1. Impact of read count cut-off on species counts.

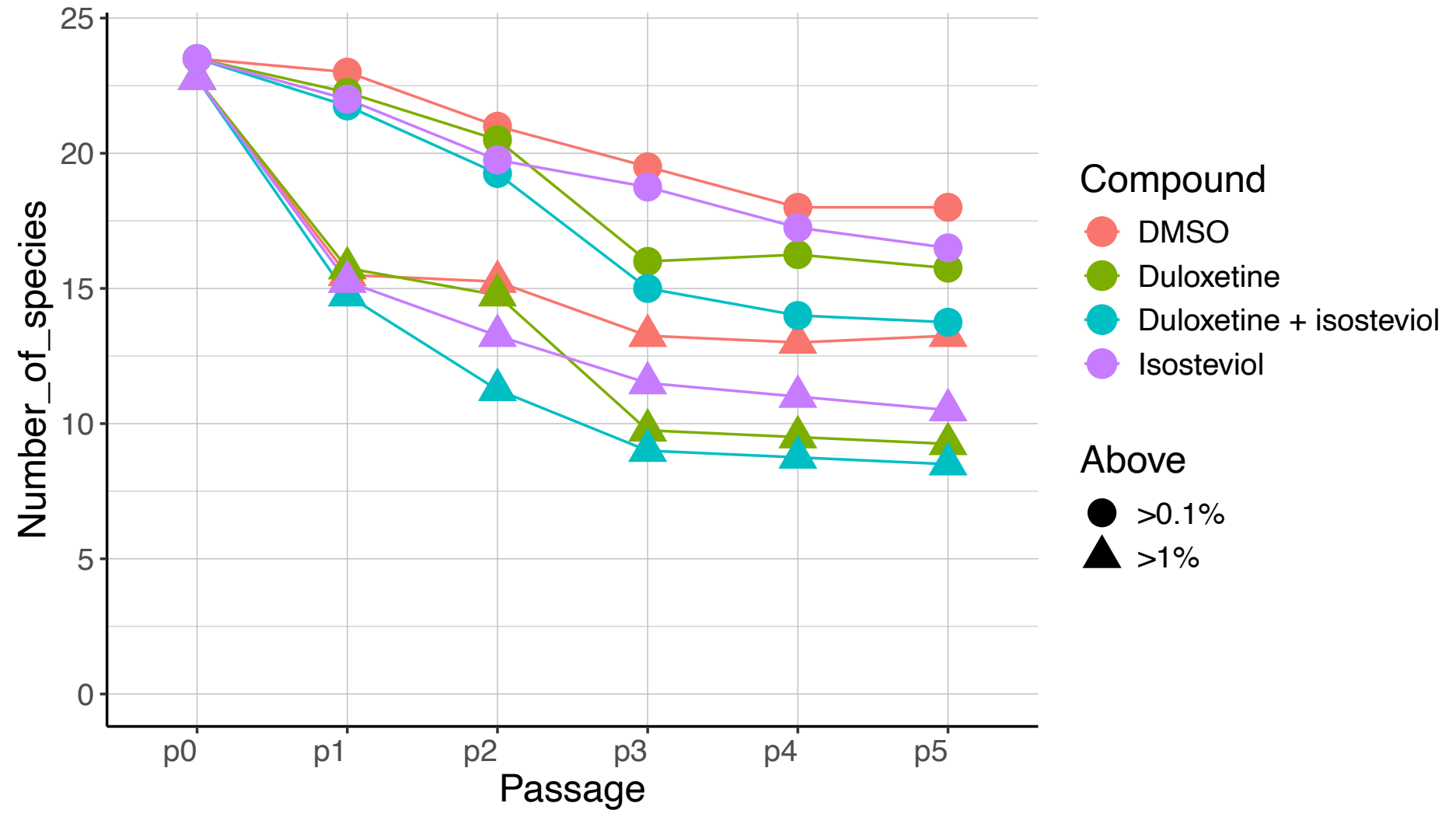

Figure 2. Number of species detected when discounting low abundant species.

Following the same rationale as Diaz et al. we also analysed our community structure at the family level (Figure 3) in addition to the higher resolution given by species level synthetic community assembly and identified the same stability observed by Diaz et al 2018.

This is clear both at a qualitative level by comparing the DMSO in our datasets (First plot left), with the community proportion plotted from Diaz et al 2022 (Fig 1b), second plot and Goldford et al. 2018, (Fig 1b), plot reproduced below in Figure 4) where no family lost is detected after the 4th transfer

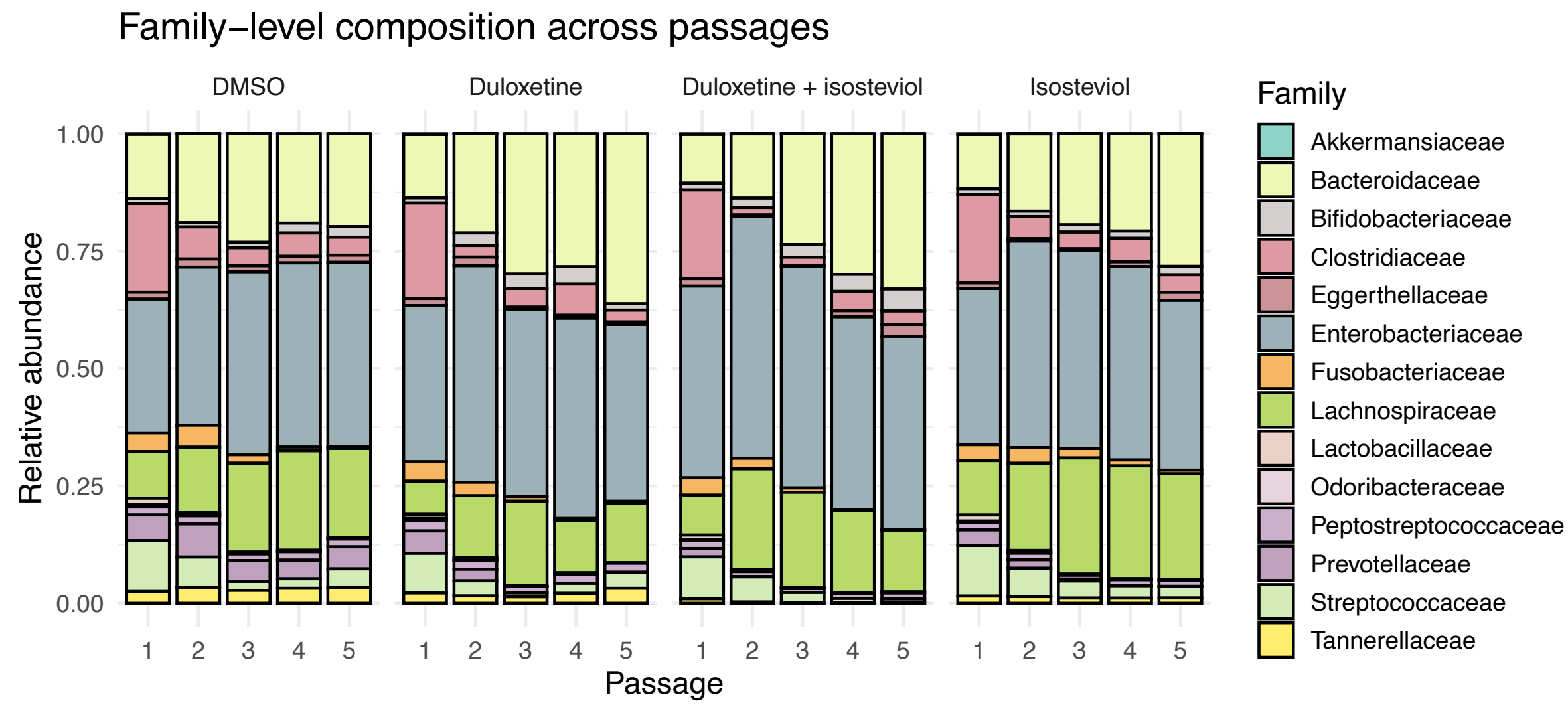

Figure 3. Bar plot representing family level proportions of the bacterial composition of the communities used in the experiment at different transfers (x-axis)

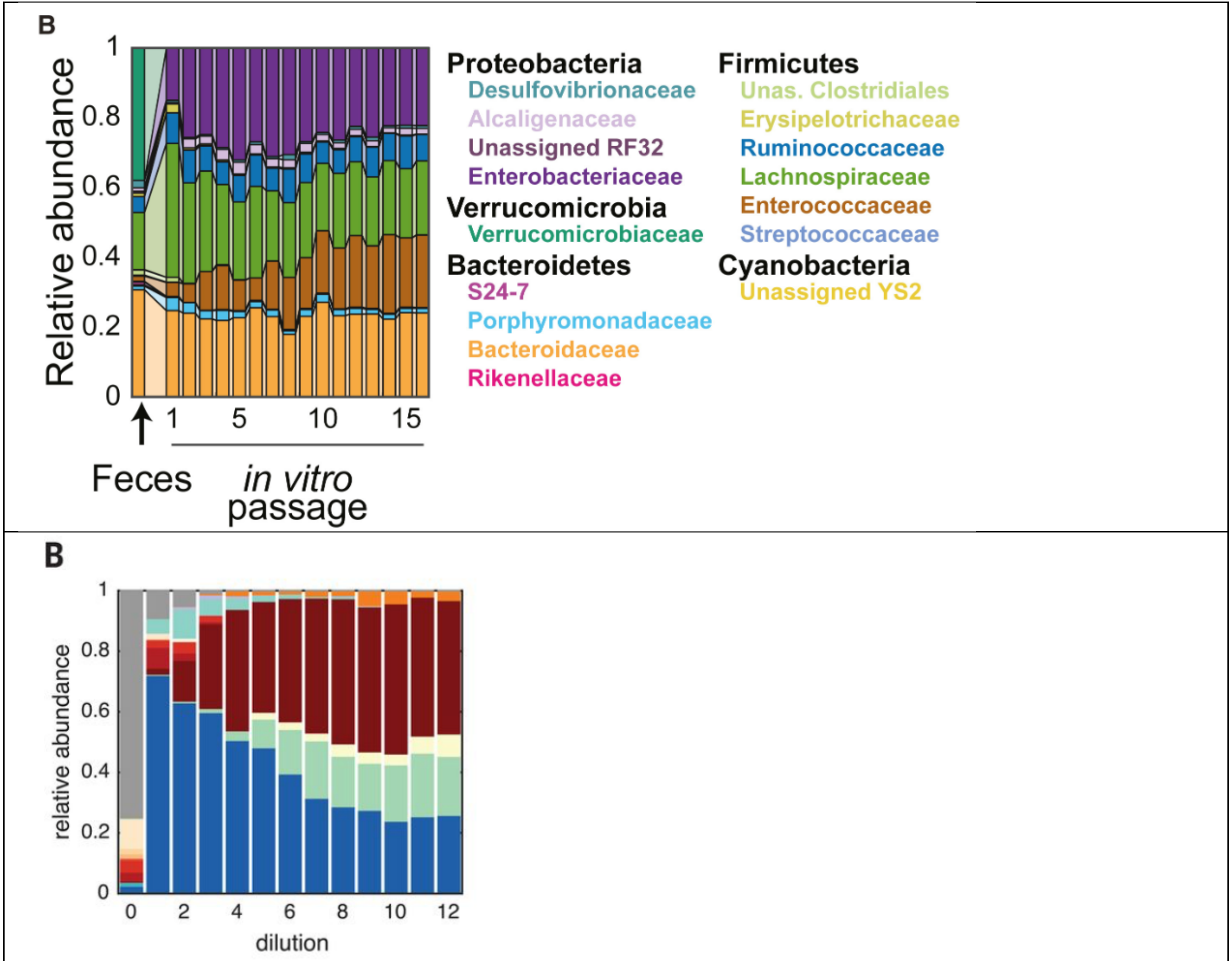

Figure 4. Family level proportions obtained from Diaz et al 2022 top plot (Figure 1b in the original paper) and Goldford et al 2018 bottom plot (Figure 1b in the original paper). The x-axis represents in both cases the transfers performed in a dilution-growth series, similar to our experiment.

In addition, we also calculated the F_{st} statistic at the family level, which confirmed the previous analysis showing overall stability (F_{st} value <0.1), with the F_{st} statistic being very low a stable already at between passage 3 and 4 (Passage 4 in the x-axis) with the biggest drop at the early stage of community assembly from. Passage 0 and 1 and passage 1 and 2 (1 and 2 labels respectively on the x axis).

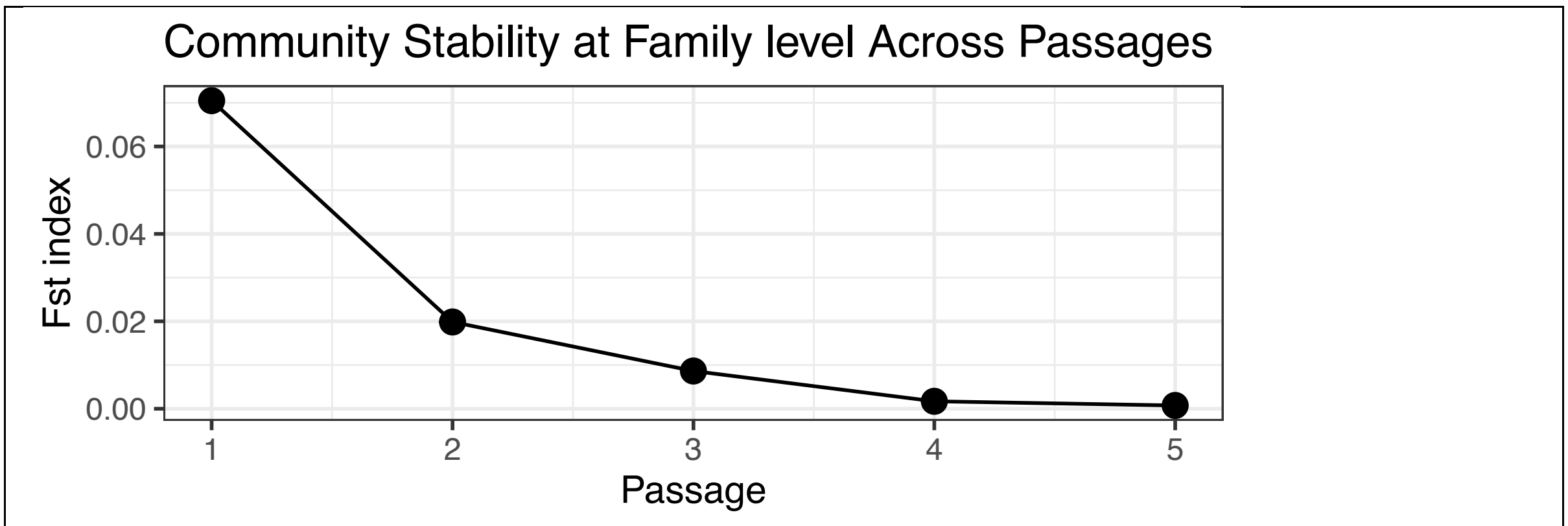

Figure 5. FAVA based analysis of the variation at the family level of the community grown in DMSO. The y-axis represents the Fst index, which represents the level of variation across transfers.

The authors state that their objective is to investigate species-level dynamics within a controlled microbial community, where changes can be more precisely quantified. However, the manuscript repeatedly refers to the "gut" including in the title, which is misleading. The community used here is composed largely of strains obtained from culture collections (e.g. DSMZ), originally isolated from stool samples, often many years ago. Such a reconstructed community does not capture the ecological complexity, functional redundancy, or host-associated context of an actual gut microbiome.
This distinction should be made explicit throughout the manuscript. At present, the terminology risks overstating the physiological relevance of the model system and may lead readers to overinterpret the findings in the context of in vivo gut microbiome dynamics.

→
We respectfully disagree with the reviewer that our manuscript is misleading in this regard. Use of “gut” in referring to gut bacteria is a common practice in microbiology literature. It is no different from referring to HepG2 cells as a “human cell line”. Further, nowhere we have claimed relevance to in vivo context based on our synthetic community.

Proposed action: We will be happy to add “*in vitro*” to the title to avoid any misinterpretation.

23rd Apr 2026

Manuscript Number: MSB-2025-13043RRR-Q
Title: Impact of low-calorie sweeteners on gut bacteria is modulated by common xenobiotics

Dear Prof Patil,

Thank you for your response to the editorial decision on your manuscript. I have now carefully examined the arguments provided in your letter and discussed them with the other members of our editorial team. I have also received arbitrating advice from an expert, who offered the advice below. I am pleased to inform you that we will be able to accept your manuscript pending the revisions planned in your rebuttal, the following final amendments, and appropriate response to the arbitrating reviewer:

1) In the main manuscript file, please include keywords to max. 5.
2) Please rename the 'Code and data availability' section to 'Data availability' (despite the fact that code information is also included). This section needs to be formatted according to the example below, with the specific URLs for PXD045140, MTBLS11480, PRJEB76553, PRJEB76554 datasets provided:
"The datasets and computer code produced in this study are available in the following databases:
- Chip-Seq data: Gene Expression Omnibus GSE46748 (https://www.ncbi.nlm.nih.gov/geo/query/acc.cgi?acc=GSE46748)
- Modeling computer scripts: GitHub (https://github.com/SysBioChalmers/GECKO/releases/tag/v1.0)
- [data type]: [full name of the resource] [accession number/identifier] ([doi or URL or identifiers.org/DATABASE:ACCESSION])"
3) Please ensure that the proteomics data are now publicly available.
4) Please include a README file on Github with practical use instructions for potential future users of your code.
5) Please rename "Conflict of Interest" to "Disclosure and competing interests statement". We updated our journal's competing interests policy in January 2022 and request authors to consider both actual and perceived competing interests. Please review the policy https://link.springer.com/partners/embo-press/editorial-policies#Competing%20interest%20disclosures and update your competing interests if necessary.
6) Please correct the reference citation in the reference list to be alphabetical (not numerical). Where there are more than 10 authors on a paper, only the first 10 should be listed, followed by "et al.". DOIs should only be used for preprints and datasets that have not been published yet. Please check "Author Guidelines" for more information:
https://link.springer.com/journal/44320/submission-guidelines#cms-Reference-guidelines
7) As per our guidelines, on "Unpublished Data" the journal does not permit citation of 'Voogdt et al., unpublished'. All data referred to in the paper should be displayed in the main or Expanded View figures. Please remove this reference and any statements based on this unpublished manuscript. In case there is a preprint available, you may reference this along with its DOI in the reference list and then would not need to remove any such statements depending on the reference.
8) In the Methods, please take care of the following:
- Please ensure that a statement on whether or not blinding was done is included in the Methods even if no blinding was done. Please also be sure to update the Author Checklist with this information and where it can be found in the manuscript.
9) For the figures and figure legends, please take care of the following:
- 'Figure captions' section to be labeled as 'Figure legends'
- Please define the annotated p values ****/***/**/* as well as provide the exact p-values for the same in the legend of figure 2C as appropriate.
- Please note that the exact p values are not provided in the legends of figures 2F, 3C, 4C, 5D, E; 6A-D.
- Please indicate the statistical test used for data analysis in the legends of figures 1B, D; 2C.
- Please note that the box plots need to be defined in terms of minima, maxima, centre, bounds of box and whiskers, and percentile in the legends of figures 1D, E; 2D, F; 5D, E; 6A, B
- Please note that information related to n is missing in the legends of figures 1C, 2D, 5D, E
- Please note that the error bars are not defined in the legends of figures 6C-E.
- Please note that the measure of center for the error bars needs to be defined in the legend of figure 1C
10) The source file names, titles, legends and manuscript callouts for Table EV1-EV32 all need to be updated to Dataset EV1-EV#. These files should be uploaded individually as Dataset files with legends in a separate tab/sheet in each Excel file. Please do not use the nomenclature with letters (Table EV3a, Table EV3b, but rather Dataset EV3, Dataset EV4...).
11) The Appendix file needs to be uploaded in PDF format. Please include on the title page "Appendix for + manuscript title" above the Table of Contents. Please also move the references after the Appendix Figure S11 legend to the end of the Appendix PDF, with the heading 'References'. In addition, please format these references the same as in the main manuscript, to be alphabetical (not numerical), and if there are more than 10 authors on a paper, only the first 10 should be listed, followed by "et al.".
12) Synopsis:
- Synopsis image: Please provide the synopsis image in a JPEG, TIFF, or PNG format and upload it as a high-resolution jpeg file 550 pixels wide x 300 pixels high.
- Please check your synopsis text and image before submission with your revised manuscript. Please be aware that in the proof stage minor corrections only are allowed (e.g., typos).

13) Source Data should be organized as a single source data file (zipped) per figure for main figures (all EV and/or Appendix figure Source Data can be included in a single folder), with the panels clearly visible in the folder structure instead of a single excel file for all Source Data. e.g. all the Source data files for figure 1 need to be saved in a single folder and this needs to be zipped and then uploaded as "SD figure 1.zip" file.
14) As part of the EMBO Publications transparent editorial process initiative (see our policy here: https://www.embopress.org/transparent-process#Review_Process), Molecular Systems Biology will publish online a Peer Review File (PRF) to accompany accepted manuscripts. This file will be published in conjunction with your paper and will include the anonymous referee reports, your point-by-point response and all pertinent correspondence relating to the manuscript. Let us know whether you agree with the publication of the PRF and as here, if you want to remove or not any figures from it prior to publication. Please note that the Authors checklist will be published at the end of the PRF.
15) After your paper is published, we may promote it on social media. If you have any handles or hashtags for Bluesky you would like included, please let us know.
16) Please provide a point-by-point letter INCLUDING my comments as well as the reviewer's report and your detailed responses (as Word file).

I look forward to reading a new revised version of your manuscript as soon as possible.

Yours sincerely,

Poonam Bheda, PhD
Scientific Editor
Molecular Systems Biology

Arbitrating advice:

"...while it is a serious confounder that the control is unstable, the dynamics in the community composition are very different in the active compound groups in Panel 2b. Accordingly, I feel confident that the differences observed are scientifically relevant. It would also be very difficult to redo the study and as long as the limitations are discussed in the paper I would find it worthwhile to publish (considering that aspect)."

Revision #4

Arbitrating advice:

"...while it is a serious confounder that the control is unstable, the dynamics in the community composition are very different in the active compound groups in Panel 2b. Accordingly, I feel confident that the differences observed are scientifically relevant. It would also be very difficult to redo the study and as long as the limitations are discussed in the paper I would find it worthwhile to publish (considering that aspect)."

→

We thank the expert for their constructive feedback. We have addressed all the proposed changes in our previous response letter, including addition of ‘in vitro’ to the title. A tracked-changes version of the manuscript is also included with the submission.

9th Jun 2026
Manuscript number: MSB-2025-13043RRRR
Title: Common xenobiotics modulate gut microbial responses to low-calorie sweeteners in vitro

Dear Prof Patil,

Thank you again for sending us your revised manuscript. We are now satisfied with the modifications made and I am pleased to inform you that your paper has been accepted for publication.

Your manuscript will be processed for publication by EMBO Press. It will be copy edited and you will receive page proofs prior to publication. Please note that you will be contacted by Springer Nature Author Services to complete licensing and payment information.

You may qualify for financial assistance for your publication charges - either via a Springer Nature fully open access agreement or an EMBO initiative. Check your eligibility: https://link.springer.com/journal/44320/how-to-publish-with-us

Should you be planning a Press Release on your article, please notify the production team via this form in order to coordinate publication and release dates: https://forms.cloud.microsoft/e/eH4A8JVP6g

If you have any questions, please do not hesitate to contact the Editorial Office. Thank you for your contribution to Molecular Systems Biology.

Yours sincerely,

Poonam Bheda, PhD
Scientific Editor
Molecular Systems Biology

>>> Please note that it is Molecular Systems Biology policy for the transcript of the editorial process (containing referee reports and your response letter) to be published as an online supplement to each paper. If you do NOT want this, you will need to inform the Editorial Office via email immediately. More information is available here: https://link.springer.com/partners/embo-press/editorial-policies#Peer%20review

EMBO Press Author Checklist

Corresponding Author Name: Kiran R. Patil	
Journal Submitted to: Molecular Systems Biology	
Manuscript Number: MSB-2025-13043	

USEFUL LINKS FOR COMPLETING THIS FORM
The EMBO Journal - Author Guidelines
EMBO Reports - Author Guidelines
Molecular Systems Biology - Author Guidelines
EMBO Molecular Medicine - Author Guidelines

Reporting Checklist for Life Science Articles (updated January
This checklist is adapted from Materials Design Analysis Reporting (MDAR) Checklist for Authors. MDAR establishes a minimum set of requirements in transparent reporting in the life sciences (see Statement of Task: 10.31222/osf.io/9sm4x). Please follow the journal's guidelines in preparing your manuscript.
Please note that a copy of this checklist will be published alongside your article.

Abridged guidelines for figures
1. Data
The data shown in figures should satisfy the following conditions:
- the data were obtained and processed according to the field's best practice and are presented to reflect the results of the experiments in an accurate and unbiased manner.
- ideally, figure panels should include only measurements that are directly comparable to each other and obtained with the same assay.
- plots include clearly labeled error bars for independent experiments and sample sizes. Unless justified, error bars should not be shown for technical
- if n<5, the individual data points from each experiment should be plotted. Any statistical test employed should be justified.
- Source Data should be included to report the data underlying figures according to the guidelines set out in the authorship guidelines on Data

2. Captions
Each figure caption should contain the following information, for each panel where they are relevant:
- a specification of the experimental system investigated (eg cell line, species name).
- the assay(s) and method(s) used to carry out the reported observations and measurements.
- an explicit mention of the biological and chemical entity(ies) that are being measured.
- an explicit mention of the biological and chemical entity(ies) that are altered/varied/perturbed in a controlled manner.
- the exact sample size (n) for each experimental group/condition, given as a number, not a range;
- a description of the sample collection allowing the reader to understand whether the samples represent technical or biological replicates (including how many animals, litters, cultures, etc.).
- a statement of how many times the experiment shown was independently replicated in the laboratory.
- definitions of statistical methods and measures:
  - common tests, such as t-test (please specify whether paired vs. unpaired), simple χ2 tests, Wilcoxon and Mann-Whitney tests, can be unambiguously identified by name only, but more complex techniques should be described in the methods section;
  - are tests one-sided or two-sided?
  - are there adjustments for multiple comparisons?
  - exact statistical test results, e.g., P values = x but not P values < x;
  - definition of 'center values' as median or average;
  - definition of error bars as s.d. or s.e.m.

Please complete ALL of the questions below.
Select "Not Applicable" only when the requested information is not relevant for your study.

Materials

Newly Created Materials	Information included in the manuscript?	In which section is the information available? (Reagents and Tools Table, Materials and Methods, Figures, Data Availability Section)
New materials and reagents need to be available; do any restrictions apply?	Not Applicable	

Antibodies	Information included in the manuscript?	In which section is the information available? (Reagents and Tools Table, Materials and Methods, Figures, Data Availability Section)
For **antibodies** provide the following information:<br>- Commercial antibodies: RRID (if possible) or supplier name, catalogue number and or/clone number<br>- Non-commercial: RRID or citation	Not Applicable	

DNA and RNA sequences	Information included in the manuscript?	In which section is the information available? (Reagents and Tools Table, Materials and Methods, Figures, Data Availability Section)
**Short novel DNA or RNA including primers, probes**: provide the sequences.	Not Applicable	

Cell materials	Information included in the manuscript?	In which section is the information available? (Reagents and Tools Table, Materials and Methods, Figures, Data Availability Section)
**Cell lines:** Provide species information, strain. Provide accession number in repository **OR** supplier name, catalog number, clone number, and/**OR** RRID.	Yes	Methods: Cytotoxicity assay
**Primary cultures:** Provide species, strain, sex of origin, genetic modification status.	Not Applicable	
Report if the cell lines were recently **authenticated** (e.g., by STR profiling) and tested for mycoplasma contamination.	Yes	Methods: Cytotoxicity assay

Experimental animals	Information included in the manuscript?	In which section is the information available? (Reagents and Tools Table, Materials and Methods, Figures, Data Availability Section)
**Laboratory animals or Model organisms:** Provide species, strain, sex, age, genetic modification status. Provide accession number in repository **OR** supplier name, catalog number, clone number, **OR** RRID.	Not Applicable	
**Animal observed in or captured from the field:** Provide species, sex, and age where possible.	Not Applicable	
Please detail **housing and husbandry conditions**.	Not Applicable	

Plants and microbes	Information included in the manuscript?	In which section is the information available? (Reagents and Tools Table, Materials and Methods, Figures, Data Availability Section)
**Plants:** provide species and strain, ecotype and cultivar where relevant, unique accession number if available, and source (including location for collected wild specimens).	Not Applicable	
**Microbes:** provide species and strain, unique accession number if available, and source.	Yes	Suppl. table2 contains a complete list of all strains used in this study.

Human research participants	Information included in the manuscript?	In which section is the information available? (Reagents and Tools Table, Materials and Methods, Figures, Data Availability Section)
If collected and within the bounds of privacy constraints report on age, sex and gender or ethnicity for all study participants.	Not Applicable	

Core facilities	Information included in the manuscript?	In which section is the information available? (Reagents and Tools Table, Materials and Methods, Figures, Data Availability Section)
If your work benefited from core facilities, was their service mentioned in the acknowledgments section?	Yes	Authors and author contributions: Hilal Ozgur and Vladimir Benes from EMBL genomics core facility and Bini Ramachandran from MRC toxicology proteomics core facility are co-authors.

Design

Study protocol	Information included in the manuscript?	In which section is the information available? (Reagents and Tools Table, Materials and Methods, Figures, Data Availability Section)
If study protocol has been **pre-registered, provide DOI in the manuscript**. For clinical trials, provide the trial registration number **OR** cite DOI.	Not Applicable	
Report the **clinical trial registration number** (at ClinicalTrials.gov or equivalent), where applicable.	Not Applicable	

Laboratory protocol	Information included in the manuscript?	In which section is the information available? (Reagents and Tools Table, Materials and Methods, Figures, Data Availability Section)
Provide DOI OR other citation details if **external detailed step-by-step protocols** are available.	Not Applicable	

Experimental study design and statistics	Information included in the manuscript?	In which section is the information available? (Reagents and Tools Table, Materials and Methods, Figures, Data Availability Section)
Include a statement about **sample size** estimate even if no statistical methods were used.	Yes	Sample sizes are noted
Were any steps taken to minimize the effects of subjective bias when allocating animals/samples to treatment (e.g. **randomization procedure**)? If yes, have they been described?	Not Applicable	
Include a statement about **blinding** even if no blinding was done.	Yes	A separate section was added to methods with the header "Blinding", stating "No blinding was done in experimental work or analysis."
Describe **inclusion/exclusion criteria** if samples or animals were excluded from the analysis. Were the criteria pre-established?<br><br>If sample or data points were omitted from analysis, report if this was due to attrition or intentional exclusion and provide justification.	Yes	To be found in Methods and Suppl. table4a: Out of 39,313 screened samples, 492 wells showing technical errors and 136 failed fits (<2% in total) were removed from further analysis, making a total of 38,685 samples entering further analysis (see supplementary table 4a for the complete dataset including the excluded wells)..
For every figure, are **statistical tests** justified as appropriate? Do the data meet the assumptions of the tests (e.g., normal distribution)? Describe any methods used to assess it. Is there an estimate of variation within each group of data? **Is** the variance similar between the groups that are being statistically compared?	Yes	See main text, figure legends, methods and supplementary tables for tests used.

Sample definition and in-laboratory replication	Information included in the manuscript?	In which section is the information available? (Reagents and Tools Table, Materials and Methods, Figures, Data Availability Section)
In the figure legends: state number of times the experiment was **replicated** in laboratory.	Yes	Figure 1,2,3,4,5 and 6
In the figure legends: define whether data describe **technical or biological replicates**.	Yes	Figure 1b, c. If not otherwise stated replicates are biological replicates.

Ethics

Ethics	Information included in the manuscript?	In which section is the information available? (Reagents and Tools Table, Materials and Methods, Figures, Data Availability Section)
Studies involving **human participants**: State details of **authority granting ethics approval** (IRB or equivalent committee(s), provide reference number for approval.	Not Applicable	
Studies involving **human participants**: Include a statement confirming that **informed consent** was obtained from all subjects and that the experiments conformed to the principles set out in the WMA Declaration of Helsinki and the Department of Health and Human Services Belmont Report.	Not Applicable	
Studies involving **human participants:** For publication of **patient photos**, include a statement confirming that consent to publish was obtained.	Not Applicable	
Studies involving experimental **animals**: State details of **authority granting ethics approval** (IRB or equivalent committee(s), provide reference number for approval. Include a statement of compliance with ethical regulations.	Not Applicable	
Studies involving **specimen and field samples:** State if relevant **permits** obtained, provide details of authority approving study; if none were required, explain why.	Not Applicable	

Dual Use Research of Concern (DURC)	Information included in the manuscript?	In which section is the information available? (Reagents and Tools Table, Materials and Methods, Figures, Data Availability Section)
Could your study fall under dual use research restrictions? Please check biosecurity documents and list of **select agents and toxins** (CDC): https://www.selectagents.gov/sat/list.htm	Not Applicable	
If you used a select agent, is the security level of the lab appropriate and reported in the manuscript?	Not Applicable	
If a study is subject to dual use research of concern regulations, is the name of the **authority granting approval and reference number** for the regulatory approval provided in the manuscript?	Not Applicable	

Reporting

The MDAR framework recommends adoption of discipline-specific guidelines, established and endorsed through community initiatives. Journals have their own policy about requiring specific guidelines and recommendations to complement MDAR.

Adherence to community standards	Information included in the manuscript?	In which section is the information available? (Reagents and Tools Table, Materials and Methods, Figures, Data Availability Section)
State if relevant guidelines or checklists (e.g., **ICMJE, MIBBI, ARRIVE, PRISMA**) have been followed or provided.	Not Applicable	
For **tumor marker prognostic studies,** we recommend that you follow the **REMARK** reporting guidelines (see link list at top right). See author guidelines, under 'Reporting Guidelines'. Please confirm you have followed these guidelines.	Not Applicable	
For **phase II and III randomized controlled trials**, please refer to the **CONSORT** flow diagram (see link list at top right) and submit the CONSORT checklist (see link list at top right) with your submission. See author guidelines, under 'Reporting Guidelines'. Please confirm you have submitted this list.	Not Applicable	

Data Availability

Data availability	Information included in the manuscript?	In which section is the information available? (Reagents and Tools Table, Materials and Methods, Figures, Data Availability Section)
Have **primary datasets** been deposited according to the journal's guidelines (see 'Data Deposition' section) and the respective accession numbers provided in the Data Availability Section?	Yes	See section "Data availability".
Were **human clinical and genomic datasets** deposited in a public access-controlled repository in accordance to ethical obligations to the patients and to the applicable consent agreement?	Not Applicable	
Are **computational models** that are central and integral to a study available without restrictions in a machine-readable form? Were the relevant accession numbers or links provided?	Not Applicable	
If publicly available data were reused, provide the respective **data citations in the reference list**.	Not Applicable	
