# Supplementary material for: Common xenobiotics modulate gut microbial responses to low‑calorie sweeteners in vitro
Source: Mol Syst Biol. 2026 Jun 25;22(9):1453–73. doi: 10.1038/s44320-026-00225-6 (PMC13538661; doi:10.1038/s44320-026-00225-6)
Supplement: Supplementary file 4 [file 44320_2026_225_MOESM49_ESM.zip › Fig2_source-data/Fig2e/20240811_Mix1_BC_beta.pdf]

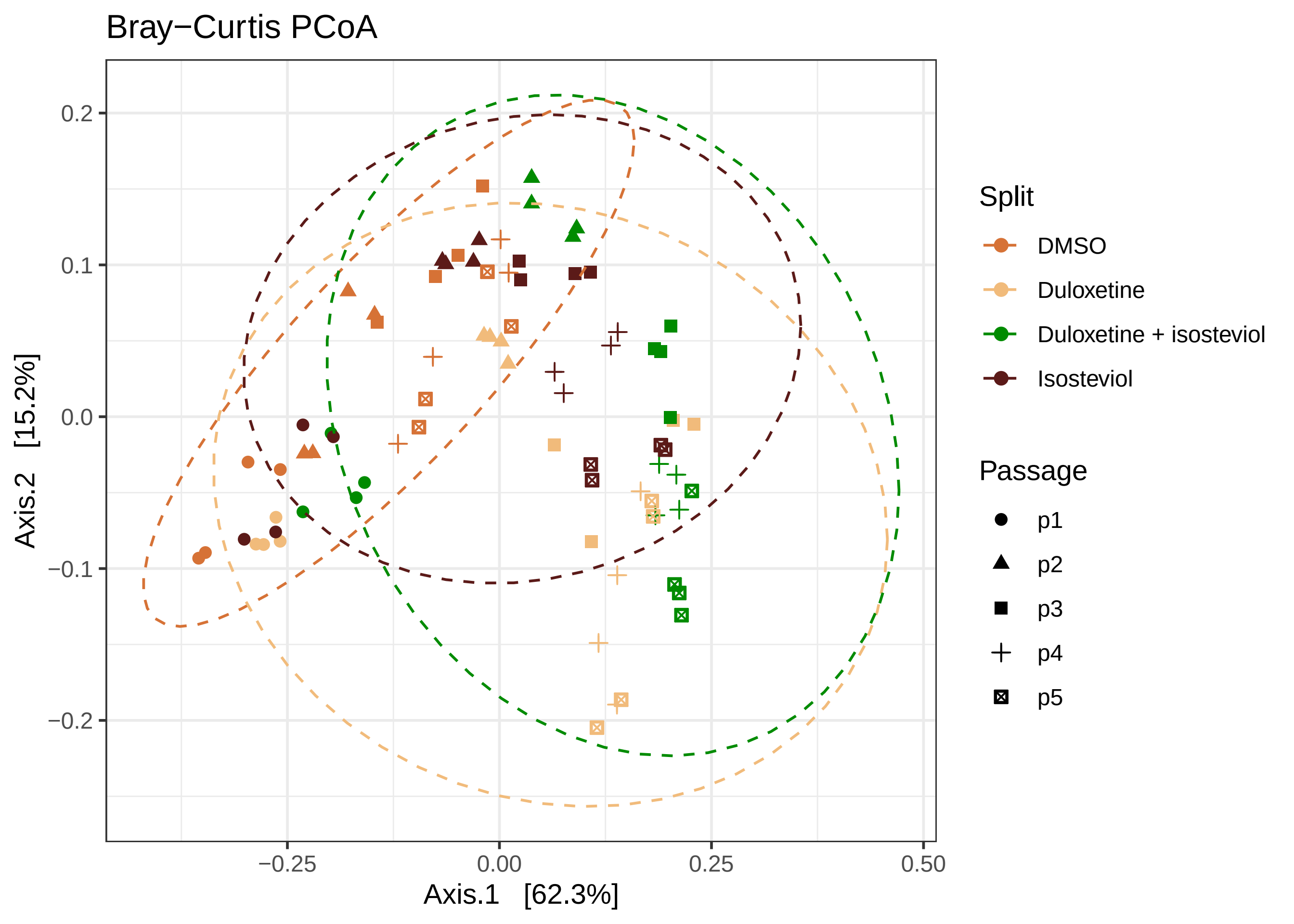
Bray−Curtis PCoA
0.2
0.1
0.0
−0.1
−0.2
Axis.2 [15.2%]
−0.25
0.00
0.25
0.50
Axis.1 [62.3%]
Split
DMSO
Duloxetine
Duloxetine + isosteviol
Isosteviol
Passage
p1
p2
p3
p4
p5
